# The contributions of active and passive smoking to COPD-related mortality and DALYs in the context of COVID-19: Global Burden 2019–2021

**DOI:** 10.18332/tid/221062

**Published:** 2026-06-10

**Authors:** Shiyu Hu, Xiaoli Tan, Danying Zhang, Ye Zhang, Xiaodong Lv, Wenyu Chen

**Affiliations:** 1 Department of Respiratory, The Affiliated Hospital of Jiaxing University, Jiaxing, China; 2 Department of Nursing, The Affiliated Hospital of Jiaxing University, Jiaxing, China

**Keywords:** chronic obstructive pulmonary disease, global disease burden, risk factor

## Abstract

**INTRODUCTION:**

Chronic obstructive pulmonary disease (COPD) is a major global health issue with high morbidity and mortality. While assessing the overall burden of COPD, it is essential to further quantify the contributions of active and passive smoking to COPD-related mortality and DALYs.

**METHODS:**

By utilizing COPD data from the 2021 Global Burden of Disease study, we analyzed the age-standardized incidence (ASIR), prevalence (ASPR), mortality (ASMR) and disability-adjusted life years (ASDR) rates (per 100000), and the risk factors associated with mortality due to COPD. Additionally, we calculated the estimated annual percentage changes (EAPCs) during the study period.

**RESULTS:**

In 2021, global COPD incidence, prevalence, deaths and disability-adjusted life years (DALYs), increased compared to those in 2019, reaching 16.9 million (95% UI: 15.5–18.3 million), 213 million (95% UI: 195.0–234.0 million), 3.7 million (95% UI: 3.3–4.1), and 79.8 million (95% UI: 74.0–86.0), respectively. The ASPR, ASMR and ASDR declined to 2512.9 (95% UI: 2293.9–2748.5), 45.2 (95% UI: 40.6–49.7), and 940.7 (95% UI: 871.5–1014.6), respectively, while the ASIR slightly increased compared to 2019. In 2021, tobacco use caused 1510389 deaths and 31613440 DALYS from COPD worldwide (ASMR: 18; ASDR: 370). Smoking accounted for 1334961 COPD deaths and 27794778 COPD DALYs (ASMR: 16; ASDR: 326), while secondhand smoke exposure resulted in 266125 COPD deaths and 5662905 COPD DALYs (ASMR: 3; ASDR: 67).

**CONCLUSIONS:**

COPD remains a global challenge. The outbreak of COVID-19 does not appear to have altered the trend of COPD disease burden changes but continued monitoring and adaptive management are essential. Meanwhile, in the context of the COVID-19 pandemic, smoking and tobacco exposure remain important risk factors contributing to the burden of COPD, underscoring the urgent need for heightened attention from the public and relevant authorities.

## INTRODUCTION

Chronic obstructive pulmonary disease (COPD) is a type of heterogeneous lung disease characterized by chronic respiratory symptoms (such as dyspnea, cough, and sputum production or exacerbation) that are caused by abnormalities in the airways and/or alveoli, leading to persistent airflow limitation^1^. The social burden of COPD is generally measured by incidence, mortality, and disability-adjusted life years (DALYs). The global prevalence of COPD in people aged > 40 years is over 9–10%, and these data are usually derived from statistics of various medical data, including doctor visits, emergency visits, and hospitalization records^2-4^. In 2019, a global report documented 212.3 million cases of COPD, with 3.3 million deaths, and a global ASMR of 42.5 (95% UI: 37.6–46.3)^5^, making COPD the third leading cause of death worldwide^6^. DALYs refer to the entire number of healthy life years lost due to the disease from the onset of the disease to the end of death (or survival at the time of evaluation) due to the disease^7^. From 1990 to 2019, COPD was a major contributor to the increase in global DALYs, with COPD-related DALYs increasing by 25.7%. In 2019, the global ASDR for COPD was 926.1 (95% UI: 848.8–997.7). The DALY rate and mortality rate of COPD patients vary by region, with Oceania having the highest ASMR (112.1) and ASDR (2309.9) from COPD^5^. From 1990 to 2019, Southeast Asia, India, Sub-Saharan Africa, and the Americas saw the largest increases in ASDR^5,8,9^. The high incidence, high mortality, and high DALY rates of COPD have resulted in a significant economic burden. Research on major world economies has revealed that COPD costs account for approximately 56% (386 billion euros) of respiratory disease costs in the European Union^1^. COPD costs in the United States are expected to continue to rise over the next 20 years, with estimated total costs of around $800.9 billion^1^. The median direct medical cost for Chinese COPD patients is $150 to $2014 per person per year^10^. Improving the prevention and treatment levels of COPD patients is an essential aspect of reducing the global disease burden. Shifting the diagnostic gateway of COPD is a key measure to improve the prevention and treatment level of COPD, which is of great significance in delaying the progression, improving prognosis, and reducing the disease burden of COPD^11,12^. Moreover, regularly assessing the global disease burden of COPD patients, timely understanding the dynamic changes of the global disease burden of COPD, and adjusting and improving existing prevention and treatment strategies promptly are also crucial for reducing the disease burden of COPD.

At the end of 2019, the outbreak of COVID-19 changed the medical practice of the entire world^13^. For COPD patients, the COVID-19 pandemic had greatly affected routine management, diagnosis, and treatment activities of COPD. Epidemiological surveys have revealed that COPD patients were a major risk group for infection with the SARS-CoV-2. Compared to non-COPD patients, COPD patients have a significantly increased risk of ICU admission and a roughly 2.5 times higher risk of death^14^. Although the COVID-19 epidemic has been effectively controlled, some countries and regions continue to report new cases and deaths of COVID-19^15^. This indicates that COPD patients may be affected by SARS-CoV-2 for a long time, so the analysis of the burden of COPD disease during the COVID-19 period is extremely valuable. Based on data from the GBD 2021 study, we comprehensively analyzed variations in the incidence, prevalence, deaths, and DALYs associated with COPD during the initial two years after the pandemic emerged.

Notably, among the various risk factors for COPD, tobacco exposure is closely associated with the onset, progression, and mortality risk of the disease and represents one of the most important and modifiable risk factors. In addition to active smoking, secondhand smoke exposure has also been linked to chronic respiratory inflammation, declines in lung function, and an increased risk of adverse COPD-related outcomes^16^.

Previous studies have shown that smoking increases the risk of developing COPD among middle-aged adults within five years, and that these individuals also face a higher risk of severe exacerbations and premature mortality over a 40-year period^17^. Moreover, evidence suggests that cigarette smoke may attenuate the anti-inflammatory effects of inhaled corticosteroids, thereby reducing treatment responsiveness in patients with COPD and leading to unfavorable outcomes^18^. On the other hand, a study by Osman et al.^19^ reported a significant association between secondhand smoke exposure and worsening health status in patients with COPD^19^, further highlighting the detrimental impact of cigarette smoke on COPD. Therefore, while assessing the overall burden of COPD, it is essential to further quantify the contributions of active and passive smoking to COPD-related mortality and DALYs, which is critical for interpreting changes in disease burden and for informing more targeted prevention and control strategies.

## METHODS

### Overview and data collection

This secondary dataset analysis was approved by the Ethics Committee of the First Hospital of Jiaxing (Ethics Number: 2024-KY-589; Date: 12 July 2024). The Ethics Committee of the First Hospital of Jiaxing agreed to waive informed consent as the study only involved data analysis without identifiable personal information.

Existing data on COPD, standardized disease definitions, and prevalence information were collected using the Global Burden of Disease Collaborator’s Global Health Data Exchange query tool. The most recent update of the GBD database was on 16 May 2024. The latest version of the GBD database assessed the morbidity, mortality, and DALYs of 371 diseases and injuries in 204 countries and territories from 1990 to 2021, providing corresponding estimates and uncertainty intervals^2,20^. In this study, we examined the latest updated data and collected data on COPD incidence, prevalence, and deaths and DALYs from COPD, and their respective age-standardized rate (ASR) globally, regionally, and nationally. We also collected data on global risk factors contributing to COPD-related mortality rates. This work followed the Strengthening the Reporting of Observational Studies in Epidemiology (STROBE) reporting guidelines^21^ (Supplementary file).

### Sociodemographic index

The SDI is a composite index mainly composed of three key elements: income level, education level, and fertility rate, aiming to provide a standardized way to compare the differences in social and demographic characteristics among different countries and regions and to measure the social and demographic development status of a country or region^22^. The range of SDI values is from 0 to 1, where 0 represents the lowest level of sociodemographic development and 1 represents the highest level of development^2^.

### Statistical analysis

The number of incident cases, deaths, DALYs, and their corresponding rates are the main indicators describing the burden of COPD. According to the GBD algorithm, each rate is reported per 100000 people, along with a 95% UI. In the GBD framework, uncertainty intervals (UIs) are reported to capture both sampling error and model-based uncertainty. For each quantity estimated, the GBD modeling process generates 1000 posterior draws. The 2.5th and 97.5th percentiles of the ordered draws are then taken as the lower and upper limits of the 95% UI^23^. Thus, all point estimates in our study, including incidence, prevalence, mortality and DALYs, are accompanied by their 95% UIs to ensure transparent presentation of uncertainty associated with data sparsity and modeling. The dynamics of COPD are analyzed by calculating estimated annual percentage changes (EAPCs) to determine the time trends of disease burden. The linear modeling is applied to determine the 95% confidence interval (95% CI) of EAPCs^24^. When both the EAPCs and their 95% CI upper limits are negative, the corresponding rates show a decreasing trend. Conversely, if both the EAPC and their 95% CI lower limits are positive, the corresponding rates show an increasing trend. In addition, if the 95% CI of the EAPC includes 0, it indicates that the change is not statistically significant^22^. We used Spearman rank correlation and linear regression analysis to explore the relationship of SDI with ASMR and ASDR in COPD patients^25^. In this study, the R software package (version4.2.3) and JD_GBDR (V2.22, Jingding Medical Technology Co., Ltd.) were used for the drawing of the figures.

## RESULTS

### Global trends

In 2021, the global number of COPD cases was 16.9 million (95% UI: 15.5–18.3 million), with the ASIR of 197.37 (95% UI: 181.65–213.42). Compared to 2019, there was no significant change in the global ASIR of COPD in 2021 [2019: 197.30 (95% UI: 181.85–213.09); EAPCs: 0.02 (95% CI: -1.08–1.14)] (Supplementary file Table S1). In 2021, the number of COPD cases reached 213 million (95% UI: 195–234 million), with an ASPR of 2512.86 (95% UI: 2293.93–2748.52). The ASPR slightly decreased compared to 2019 [2019: 2520.67 (95% UI: 2306.51–2756.83); EAPCs: -0.15 (95% CI: -1.54–1.25)] (Supplementary file Table S2).

In 2021, COPD resulted in 3.72 million deaths (95% UI: 3.35–4.08 million), with the corresponding ASMR decreasing from 46.09 in 2019 (95% UI: 41.81–49.66) to 45.22 in 2021 (95% UI: 40.61–49.70), with EAPCs of -0.95 (95% CI: -2.70–0.83) (Table 1).

**Table 1:** The deaths and ASMR from COPD in 2019 and 2021, at global and regional levels

*Location*	*2019*		*2021*		
	*Deaths* *(95% UI)*	*ASMR* *(95% UI)*	*Deaths* *(95% UI)*	*ASMR* *(95% UI)*	*EAPCs* *(95% CI)*
**Global**	3597741.71 (3274670.63–3877018.07)	46.09 (41.81–49.66)	3719936.55 (3347912.32–4084218.20)	45.22 (40.61–49.70)	-0.95 (-2.70–0.83)
**SDI regions**					
High	456659.86 (399828.77–486279.16)	19.78 (17.54–20.95)	469238.51 (410389.19–501052.68)	19.44 (17.26–20.66)	-0.86 (-7.78–6.58)
High-middle	657966.88 (575156.66–737293.28)	36.05 (31.45–40.37)	689449.00 (591990.84–781694.40)	35.91 (30.78–40.69)	-0.19 (-1.14–0.78)
Middle	1221461.55 (1088362.77–1374751.47)	58.09 (51.57–65.09)	1293660.10 (1122165.50–1476736.57)	57.45 (49.59–65.43)	-0.55 (-3.71–2.71)
Low-middle	994637.79 (899501.73–1094465.86)	88.35 (79.43–97.53)	999762.37 (898211.09–1104472.17)	84.76 (75.80–93.78)	-2.05 (-3.81 – -0.26)
Low	265223.65 (236581.90–295886.44)	73.86 (66.15–82.45)	266003.52 (237374.65–300954.63)	70.70 (63.35–79.76)	-2.16 (-5.15–0.93)
**Regions**					
Andean Latin America	8046.19 (6775.27–9603.78)	14.72 (12.41–17.57)	7390.43 (6022.51–8969.89)	13.40 (10.93–16.29)	-4.58 (-17.01–9.71)
Australasia	11137.77 (9716.97–12028.02)	19.63 (17.24–21.14)	11514.97 (10072.77–12490.73)	18.89 (16.66–20.42)	-1.88 (-31.17–39.87)
Caribbean	10585.88 (9563.85–11648.03)	20.10 (18.17–22.14)	10847.40 (9545.67–12273.91)	19.85 (17.47–22.49)	-0.63 (-2.66–1.45)
Central Asia	14292.31 (13236.46–15378.93)	21.92 (20.22–23.56)	14472.30 (12972.34–16004.45)	21.27 (19.12–23.46)	-1.49 (-3.53–0.59)
Central Europe	37924.72 (35490.11–39551.44)	16.32 (15.29–17.00)	37944.37 (34726.90–40751.70)	16.03 (14.70–17.22)	-0.90 (-4.21–2.53)
Central Latin America	59722.48 (54580.43–62496.55)	27.09 (24.74–28.34)	62380.92 (54936.77–68713.47)	26.92 (23.70–29.64)	-0.32 (-8.07–8.08)
Central Sub-Saharan Africa	15611.53 (10970.41–22988.63)	42.85 (30.02–66.39)	16404.54 (11384.81–24013.97)	42.82 (29.63–65.02)	-0.03 (-1.13–1.07)
East Asia	1237744.32 (1058470.90–1454859.95)	73.58 (63.05–86.21)	1323440.99 (1082507.99–1574211.39)	72.20 (59.32–85.26)	-0.95 (-1.82 – -0.07)
Eastern Europe	42847.84 (40624.80–44603.87)	12.09 (11.47–12.59)	42153.21 (38785.56–45390.03)	11.72 (10.79–12.62)	-1.51 (-1.86 – -1.16)
Eastern Sub-Saharan Africa	37328.34 (29973.51–43785.00)	30.31 (24.28–35.73)	38601.00 (31589.67–45499.16)	29.91 (24.41–35.14)	-0.66 (-3.16–1.90)
High-income Asia Pacific	41455.69 (34556.76–45878.09)	6.68 (5.71–7.34)	44250.88 (36742.22–49170.48)	6.68 (5.70–7.37)	-0.00 (-15.28–18.02)
High-income North America	205408.99 (180555.91–217935.69)	30.02 (26.57–31.73)	213210.91 (185696.61–226184.97)	29.89 (26.20–31.63)	-0.21 (-7.74–7.93)
North Africa and Middle East	90636.88 (82045.26–99097.57)	27.05 (24.20–29.53)	92679.97 (82194.30–103229.07)	26.37 (23.19–29.31)	-1.27 (-5.37–3.01)
Oceania	5936.70 (4826.32,7111.36)	120.30 (98.83–143.02)	6218.94 (4984.92–7671.74)	118.21 (96.32–144.59)	-0.87 (-5.08–3.52)
South Asia	1221077.06 (1104050.84–1355956.79)	106.87 (96.03–119.19)	1229609.51 (1104444.79–1381283.99)	101.63 (90.55–114.34)	-2.48 (-3.31 – -1.65)
Southeast Asia	216493.80 (193969.96–241253.16)	43.77 (39.23–48.60)	228487.39 (202410.20–257524.96)	43.14 (38.24–48.54)	-0.73 (-8.90–8.18)
Southern Latin America	21989.58 (20227.21–23289.23)	25.03 (23.07–26.50)	20618.94 (18764.22–22039.45)	22.53 (20.53–24.07)	-5.14 (-5.15 – -5.14)
Southern Sub-Saharan Africa	16369.14 (15250.85–17454.69)	35.60 (32.88–38.15)	16579.10 (15267.62–17780.86)	34.78 (31.93–37.34)	-1.16 (-5.35–3.21)
Tropical Latin America	63169.42 (56621.40–66584.85)	27.47 (24.52–29.00)	64662.04 (57768.74–68635.22)	26.32 (23.44–27.99)	-2.12 (-9.74–6.14)
Western Europe	207546.11 (180597.37–221635.02)	18.65 (16.51–19.77)	205393.48 (177939.48–219861.62)	17.82 (15.74–18.92)	-2.24 (-3.77 – -0.69)
Western Sub-Saharan Africa	32416.99 (28852.54–37471.33)	22.60 (20.24–25.69)	33075.26 (29016.90–37675.25)	22.07 (19.45–25.05)	-1.17 (-2.60–0.27)

ASMR: age-standardized mortality rate per 100000. COPD: chronic obstructive pulmonary disease. EAPCs: estimated annual percentage changes. SDI: sociodemographic index. UI: uncertainty interval. CI: confidence interval.

In 2021, the estimated number of DALYs due to COPD was 79.78 million (95% UI: 74.03–86.01), with an ASDR of 940.66 (95% UI: 871.48–1014.59), a decrease compared to 2019 [2019: 958.62 (95% UI: 889.80–1023.31); EAPCs: -0.94 (95% CI: -2.99–1.15)] (Supplementary file Table S3).

### SDI trends

**Figure 1:**
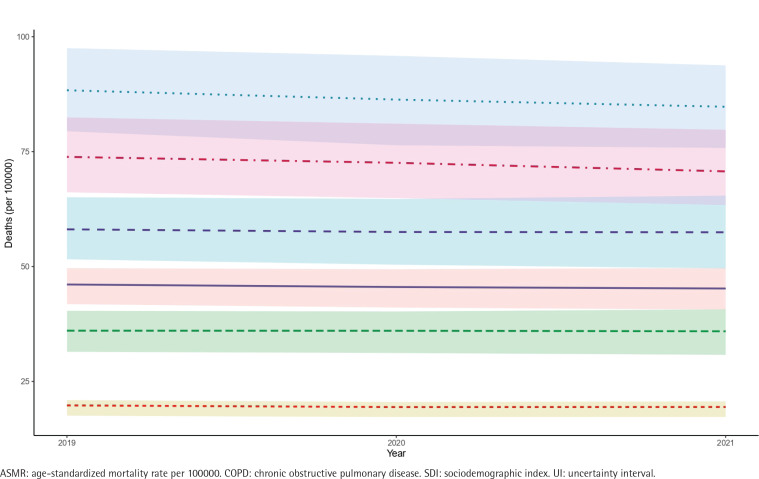
Trends of ASMR from COPD in global and five SDI regions from 2019 to 2021. Solid lines represent the estimated ASMRs, and shaded areas indicate the 95% UI

In 2021, the COPD disease burden exhibited significant regional differences. Among the five SDI regions, the low-middle SDI region had the highest ASIR, ASPR, ASMR, and ASDR, with rates of 227.24 (95% UI: 212.55–242.96), 2726.76 (95% UI: 2478.22–2,979.00), 84.76 (95% UI: 75.80–93.78), and 1707.90 (95% UI: 1558.88–1865.11), respectively. The highest number of COPD cases, deaths, and DALYs were observed in the middle SDI region, with 5.33 million cases (95% UI: 4.82–5.86 million), 63.93 million cases (95% UI: 57.32–71.32 million), 1.29 million deaths (95% UI: 1.12–1.48 million), and 26.68 million DALYs (95% UI, 23.96–29.74 million). The high-middle SDI region had the lowest ASIR, the low SDI region had the lowest ASPR, while the high SDI region had the lowest ASMR and ASDR. From 2019 to 2021, the ASMR and ASDR showed a slight downward trend across all five SDI regions, with EAPCs all being less than 0 (Table 1, Figure 1; and Supplementary file Figures S1–S3).

### Geographical regional trends

Among the 21 geographical regions, East Asia had the highest number of COPD cases, prevalence, and deaths, with 4.57 million cases (95% UI: 4.13–5.00 million), 52.09 million cases (95% UI: 46.32–58.80 million), and 1.32 million deaths (95% UI: 1.08–1.57 million), respectively. South Asia had the highest number of DALYs, with 28.01 million (95% UI: 25.40–30.94 million). In addition, South Asia also had the highest ASIR at 258.26 (95% UI: 242.49–273.76), and High-income North America had the highest ASPR, at 3298.88 (95% UI: 3132.14–3451.88). The three regions with the highest ASMR were Oceania [118.21 (95% UI: 96.32–144.59)], South Asia [101.63 (95% UI: 90.55–114.34)], and East Asia [72.20 (95% UI: 59.32–85.26)]. The regions with the highest ASDR were also the same three: [Oceania: 2351.49 (95% UI: 1931.26–2854.06), South Asia: 2049.22 (95% UI: 1862.71–2268.73), and East Asia: 1217.69 (95% UI: 1043.87–1422.79)].

**Figure 2:**
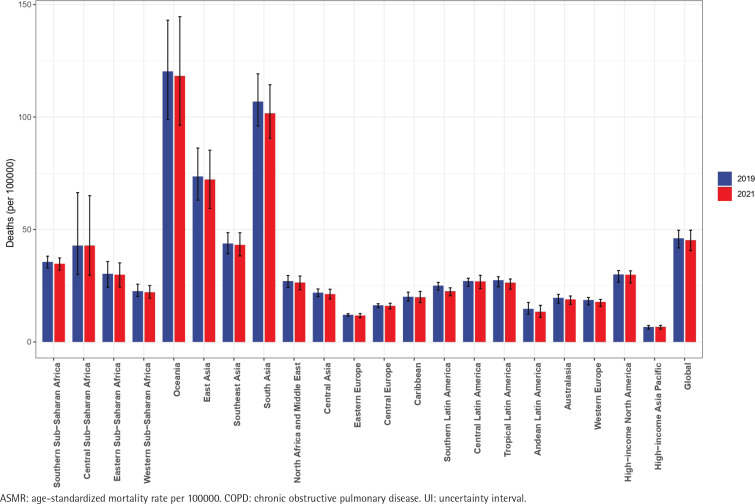
The deaths and ASMR from COPD in different geographical regions in 2019 and 2021. The error bars indicate the 95% UI around the estimated ASMR

From 2019 to 2021, the ASMR in all 21 geographical regions decreased compared to 2019, and the ASDR decreased in all regions except Central Latin America and Central Sub-Saharan Africa. In 2021, the ASIR and ASPR increased in Eastern Europe, Western SubSaharan Africa, Eastern Sub-Saharan Africa, Central Sub-Saharan Africa, North Africa and the Middle East, Tropical Latin America, Southeast Asia, Southern Sub-Saharan Africa, and Oceania compared to 2019 (Figure 2; and Supplementary file Figures S4–S6).

### National trends

**Figure 3:**
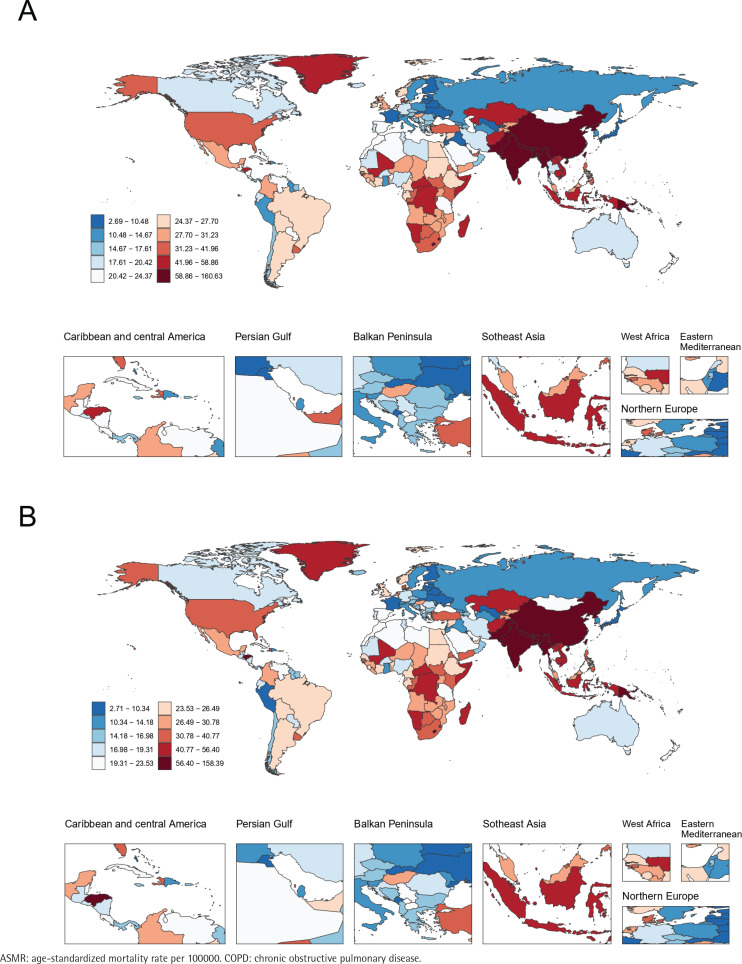
Global ASMR from COPD in 2019 (A) and 2021(B)

In 2021, among 204 countries and regions, China, India, and the United States had the highest numbers of cases, prevalence, deaths, and DALYs. The ASIR across countries ranged from 66.76 to 310.58. Nepal [310.58 (95% UI: 300.58–319.7)], Papua New Guinea [279.71 (95% UI: 263.93–294.94)], and India [262.45 (95% UI: 246.14–277.48)] had the highest ASIRs. The ASPR ranged from 922.56 to 3445.29, with the highest ASPRs in the United States [3445.29 (95% UI: 3263.49–3602.42)], the United Kingdom [3270.26 (95% UI: 2957.04–3570.38)], and Turkey [3146.68 (95% UI: 2861.31–3477.82)]. Papua New Guinea [ASMR: 156.82 (95% UI: 123.55–197.43), ASDR: 3004.36 (95% UI: 2404.29–3732.82)], Nepal [ASMR: 146.13 (95% UI: 116.66–182.46), ASDR: 2836.01 (95% UI: 2275.31–3485.04)], and India [ASMR: 108.39 (95% UI: 94.73–122.39), ASDR: 2171.16 (95% UI: 1953.69–2422.39)] had the highest ASMR and ASDR. The lowest ASMRs were in Kuwait [2.74 (95% UI: 2.24–3.28)], Japan [5.84 (95% UI: 4.93–6.33)], and Montenegro [5.87 (95% UI: 4.63–7.15)], while the lowest ASDRs were in Kuwait [160.46 (95% UI: 138.49–185.65)], Japan [155.76 (95% UI: 137.62–174.26)], and Singapore [146.48 (95% UI: 131.42–161.67)] (Tables 2 and 3, and Figure 3; and Supplementary file Tables S4–S7 and Figures S7–S9).

**Table 2:** The deaths and ASMR from COPD in the top five countries, in 2019 and 2021

*Countries*	*2019*		*2021*	
	*Deaths* *(95% UI)*	*ASMR* *(95% UI)*	*Deaths* *(95% UI)*	*ASMR* *(95 % UI)*
**China**	1200793.29 (1020270.99–1414895.54)	74.63 (63.54–87.69)	1285433.17 (1044727.8–1539819.91)	73.23 (59.73–86.85)
**India**	1062605.55 (946726.43–1183512.26)	114.37 (100.99–127.55)	1066181.25 (939670.33–1202912.45)	108.39 (94.73–122.39)
**United States**	189938.26 (166908.35–201549.07)	31.27 (27.67–33.06)	198036.53 (172625.06–210192.16)	31.32 (27.47–33.15)
**Indonesia**	80395.86 (64852.31–94214.58)	49.76 (40.55–57.34)	85259.45 (69822.72–101680.42)	49.53 (40.95–58.23)
**Bangladesh**	67429.49 (54053.92–86502.98)	61.07 (48.76–78.27)	71034.02 (54260.07–92451.09)	59.65 (46.23–77.22)

Data for all countries are available in Supplementary file Table S6. ASMR: age-standardized mortality rate per 100000. COPD: chronic obstructive pulmonary disease. UI: uncertainty interval.

**Table 3:** The number of DALYs and ASDR from COPD in the top five countries, in 2019 and 2021

*Countries*	*2019*		*2021*	
	*DALYS* *(95% UI)*	*ASDR* *(95% UI)*	*DALYS* *(95% UI)*	*ASDR* *(95 % UI)*
**India**	23842302.47 (21517259.42–26249174.72)	2274.65 (2050.21–2504.65)	24018380.31 (21633268.17–26798623.96)	2171.16 (1953.69–2422.39)
**China**	22337597.44 (19180507.73–26139106.45)	1251.83 (1077.58–1461.13)	23640320.96 (19998658.36–27921931.16)	1227.66 (1048.45–1442.54)
**United States**	4471175 (4161279.38–4726985.63)	780.32 (728.06–823.72)	4646118.12 (4302650.44–4904223.76)	777.93 (725.17–819.94)
**Indonesia**	2105801.28 (1761996.77–2440811.45)	1044.6 (871.88–1197.15)	2235813.86 (1869853.33–2637091.6)	1040.41 (874.72–1216)
**Bangladesh**	1628846.51 (1329412.28–1991745.59)	1327.58 (1086.93–1630.45)	1713712.75 (1376728.77–2133832.83)	1301.68 (1050.53–1612.22)

Data for all countries are available in Supplementary file Table S7. DALYs: disability-adjusted life years. ASDR: age-standardized DALYs rate per 100000. COPD: chronic obstructive pulmonary disease. UI: uncertainty interval.

### Age and sex pattern

**Figure 4:**
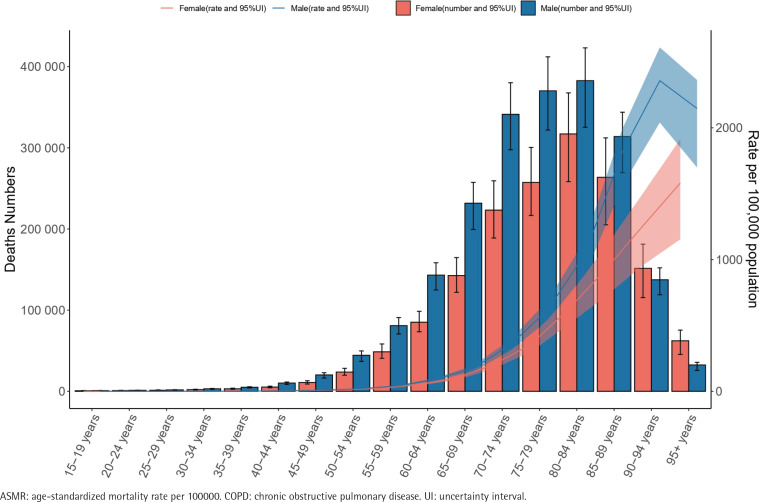
The deaths (bar plot) and ASMR (line plot) from COPD, stratified by age and sex, in 2021. The error bars indicate the deaths with 95% uncertainty intervals for men and women in different age groups

In 2021, the ASIR, ASPR, female ASMR, and female ASDR of COPD patients increased with age, reaching their highest levels in the age group >95 years. Specifically, the ASIR was 2078.94 (95% UI: 1345.73–2835.23), ASPR was 42366.01 (95% UI: 36676.77–49170.72), female ASMR was 1581.27 (95% UI: 1153.76–1914.99), and female ASDR was 14939.34 (95% UI: 11538.17–17794.61). In contrast, male ASMR [2357.79 (95% UI: 2039.83–2610.24)] and male ASDR [22345.83 (95% UI: 19515.18–24654.34)] peaked in the 90–94 years age group (Figure 4; and Supplementary file Figures S10–S12).

### Association with SDI

We found that in 2021, there was an inverted U-shaped association of SDI with ASMR and ASDR in COPD patients. As the SDI increased, the ASMR showed a trend of first increasing and then decreasing, reaching its peak at SDI of 0.45 (Supplementary file Figures S13 and S14).

### Risk factors for COPD patients

In 2021, the primary risk factors for COPD-related deaths globally were ambient ozone pollution (13.06%), high temperature (0.99%), low temperature (8.16%), occupational exposure to particulate matter, gases, and fumes (15.56%), particulate matter pollution (40.95%), secondhand smoke (7.16%), and smoking (35.65%). Among these, particulate matter pollution contributed to an ASMR of 18.51 (95% UI: 14.64–23.13). Additionally, we found that the attribution risk factors varied by region. In Oceania, Western Sub-Saharan Africa, Eastern Sub-Saharan Africa, and Central Sub-Saharan Africa, the main attribution risk factors for COPD-related deaths were occupational exposure to particulate matter, gases, and fumes, and particulate matter pollution, while in Central Europe and Eastern Europe, smoking was the primary risk factor (Supplementary file Figure S15).

In further analyses of tobacco, smoking, and secondhand smoke, we found that in 2021, tobacco exposure was responsible for 1510388.83 deaths (95% UI: 1139036.41–1872108.29) and 31613440.38 DALYs (95% UI: 23984861.88–38521071.25). The corresponding ASMR and ASDR were 18.26 (95% UI: 13.77–22.67) and 370.64 (95% UI: 280.85–452.10), respectively. Smoking accounted for 1334961.49 deaths (95% UI: 1053265.31–1596644.97) and 27794778.42 DALYs (95% UI: 22233658.14–32884383.79), with an ASMR of 16.12 (95% UI: 12.68–19.29) and an ASDR of 325.52 (95% UI: 260.32–385.52). In addition, secondhand smoke exposure resulted in 266124.70 deaths (95% UI: 106054.27–434215.02), corresponding to an ASMR of 3.24 (95% UI: 1.29–5.29), and 5662904.86 DALYs (95% UI: 2246472.20–9077004.16), with an ASDR of 66.78 (95% UI: 26.50–106.98). From 2019 to 2021, the absolute numbers of deaths and DALYs attributable to tobacco increased, whereas the corresponding ASR declined over the same period. Further stratified analyses by sex showed that in 2021, the mortality attributable to tobacco was 1161715.93 (95% UI: 904881.73–1406961.20) among males and 348672.90 (95% UI: 210507.13–496540.99) among females. The mortality attributable to smoking were 1102915.41 (95% UI: 871018.69–1325061.31) for males and 232046.09 (95% UI: 163632.30–316139.31) for females, while those attributable to secondhand smoke were 131347.84 (95% UI: 51635.78–217953.09) and 134776.87 (95% UI: 51788.39–221145.99), respectively. Notably, the sex difference in mortality attributable to secondhand smoke was substantially smaller than that observed for tobacco and smoking. The DALYs attributable to tobacco, smoking, and secondhand smoke among males were 24103410.31 (95% UI: 18915059.71–28682355.15), 22843000.96 (95% UI: 18383346.15–26947224.92), and 2724515.49 (95% UI: 1095246.44–4376779.77), respectively, whereas the corresponding DALYs among females were 7510030.07 (95% UI: 4643774.76–10407607.70), 4951777.44 (95% UI: 3630190.76–6490676.81), and 2938389.37 (95% UI: 1132101.32–4747521.66) (Tables 4 and 5; and Supplementary file Figures S16 and S17).

**Table 4:** The deaths and ASMR from COPD attributable to tobacco and smoking, in 2019 and 2021

*Location*	*2019*		*2021*	
	*Deaths* *(95% UI)*	*ASMR* *(95% UI)*	*Deaths* *(95% UI)*	*ASMR* *(95% UI)*
**Tobacco**				
**Global**	1467536.03 (1119678.7–1819765.98)	18.7 (14.24–23.24)	1510388.83 (1139036.41–1872108.29)	18.26 (13.77–22.67)
**SDI regions**				
Low	76519.01 (55430.51–97821.32)	21.43 (15.44–27.64)	75827.79 (54940.87–98238.33)	20.23 (14.67–26.35)
Low-middle	379351.2 (287989.08-477501.19)	33.35 (25.12–42.13)	379702.4 (287175.74–478140.24)	31.84 (23.85–40.08)
Middle	524933.57 (388631.74–667169.47)	24.64 (18.3–31.35)	551579.57 (404329.38–697346.1)	24.19 (17.67–30.52)
High-middle	306348.83 (231208.12–381052.53)	16.6 (12.49–20.68)	319955.83 (236239.13–400388.71)	16.51 (12.13–20.61)
High	179701.3 (134774.69–226276.05)	8.03 (6.09–10.01)	182635.69 (137069.51–230487.44)	7.81 (5.9–9.75)
**Smoking**				
**Global**	1298083.79 (1036986.29– 1556061.29)	16.52 (13.19–19.83)	1334961.49 (1053265.31–1596644.97)	16.12 (12.68–19.29)
**SDI regions**				
Low	64536.89 (49555.42–79619.17)	18.03 (13.81–22.44)	63862.83 (47922.39–80299.89)	16.97 (12.71–21.25)
Low-middle	328730.24 (262897.13–398839.09)	28.79 (22.95–35.01)	328951.08 (261545.1–400497.13)	27.47 (21.81–33.52)
Middle	458951.41 (356123.4–563266.68)	21.45 (16.5–26.33)	481590.8 (367864.93–592352)	21.02 (16.13–25.85)
High-middle	274285.24 (214741.9–333829.56)	14.84 (11.61–18.06)	286316.67 (218773.88–352191.57)	14.74 (11.25–18.09)
High	170975.56 (130463.42–213244.19)	7.65 (5.89–9.45)	173631.61 (132273.79–216561.71)	7.43 (5.73-9.18)
**Secondhand smoke**				
**Global**	257434.9 (101905.35–417082.18)	3.3 (1.31–5.36)	266124.7 (106054.27–434215.02)	3.24 (1.29–5.29)
**SDI regions**				
Low	16244.07 (6402.06–26623.58)	4.63 (1.85–7.58)	16190.95 (6161.47–26927.79)	4.41 (1.7–7.27)
Low-middle	74395.57 (28802.05–122120.21)	6.68 (2.63–10.97)	74546.14 (29434.4–123115.06)	6.39 (2.52–10.56)
Middle	99150.33 (39801.06–163048.18)	4.78 (1.92–7.87)	104890.54 (42778.94–172646.77)	4.72 (1.9–7.8)
High-middle	52895.02 (21210.74–88330.31)	2.9 (1.16–4.85)	55393.03 (22274.34–92140.47)	2.89 (1.16–4.8)
High	14632.79 (5778.76–24179.01)	0.65 (0.26–1.07)	14985.48 (5966.28–24623.12)	0.64 (0.25–1.05)

ASMR: age-standardized mortality rate per 100000. COPD: chronic obstructive pulmonary disease. SDI: sociodemographic index. UI: uncertainty interval.

**Table 5:** The number of DALYs and ASDR from COPD attributable to tobacco and smoking, in 2019 and 2021

*Location*	*2019*		*2021*	
	*DALYS* *(95% UI)*	*ASDR* *(95% UI)*	*DALYS* *(95% UI)*	*ASDR* *(95 % UI)*
**Tobacco**				
**Global**	30857419.99 (23488593.33–37912137.08)	380.83 (289.74–468.15)	31613440.38 (23984861.88–38521071.25)	370.64 (280.85–452.1)
**SDI regions**				
Low	1764071.07 (1285582.5–2249691.43)	420.87 (306.17–535.77)	1772356.75 (1275087.46–2277800.37)	400.87 (290.61–515.26)
Low-middle	8334530.98 (6336511.75–10413079.02)	660.07 (501.66–826.84)	8359965.98 (6345509.13–10448054.09)	630.34 (477.28–790.4)
Middle	10515304.11 (7962615.70–13221555.92)	449.51 (339.27–565.89)	10972299.59 (8162421.54–13642647.19)	439.52 (326.02–546.95)
High-middle	6018922.83 (4612045.22–7420888.75)	320.1 (244.84–395)	6242574.83 (4703376.44–7626774.25)	316.53 (237.88–386.51)
High	4207883.18 (3205174.37–5184504.12)	206.27 (157.95–253.36)	4249394.61 (3254455.01–5230156.48)	200.2 (154.03–245.28)
**Smoking**				
**Global**	27157475.34 (21773126.22–32465636.96)	334.93 (268.34–400.92)	27794778.42 (22233658.14–32884383.79)	325.52 (260.32–385.52)
**SDI regions**				
Low	1471405.92 (1149043.61–1808943.21)	352.67 (274.24–433.97)	1475151.82 (1136337.66–1841682.85)	335.21 (255.29–419.43)
Low-middle	7176799.88 (5793886.02–8661565.24)	568.36 (457.62–684.21)	7193010.61 (5755463.37–8715261.67)	542.25 (432.86–659.2)
Middle	9125291.71 (7220266.04–11120406.63)	389.25 (307.54–475.2)	9509875.21 (7476608.61–11518051.56)	379.97 (297.64–460.97)
High-middle	5365608.69 (4295471.36–6443048.84)	284.76 (228.11–342.31)	5561632.49 (4428561.9–6756886.42)	281.31 (223.53–341.61)
High	4003506.84 (3115800.73–4887467.44)	195.99 (153.52–238.66)	4040145.92 (3159836.97–4936268.74)	190.05 (149.49–230.91)
**Secondhand smoke**				
**Global**	5500514.47 (2141281.93–8841506.59)	68.16 (26.49–109.6)	5662904.86 (2246472.2–9077004.16)	66.78 (26.5–106.98)
Low	387908.25 (148907.27–635656.22)	91.39 (35.54–149.6)	392852.57 (148021.95–647490.07)	87.74 (33.27–144.52)
Low-middle	1667776.18 (632238.5–2719685.04)	132.55 (50.64–215.79)	1678458.37 (651581.88–2742202.54)	127.09 (49.53–208.12)
Middle	2032211.48 (814289.96–3281081.02)	88.02 (35.05–142.72)	2132844.46 (869137.14–3478022.09)	86.7 (35.29–142.01)
High-middle	1054731.98 (421657.34–1709526.4)	56.7 (22.69–92.03)	1096295.35 (438799.79–1760622.94)	56.3 (22.6–90.3)
High	355039.77 (141145.19–578838.1)	17.99 (7.18–29.32)	359565.29 (144011.77–585561.32)	17.55 (7.06–28.52)

DALYs: disability-adjusted life years. ASDR: age-standardized disability-adjusted life years rate per 100000. COPD: chronic obstructive pulmonary disease. SDI: sociodemographic index. UI: uncertainty interval.

## DISCUSSION

COPD is a major global public health problem, and the global investment in COPD prevention and treatment is increasing every year, resulting in a lot of economic and social burden to all countries^26^. The sudden outbreak and epidemic of COVID-19 at the end of 2019 has brought great challenges to the global management of chronic diseases, which is another global public health problem worthy of attention^13^. In this context, we aimed to ascertain whether the burden of COPD disease showed new trends. We analyzed the ASIR, ASPR, ASMR, ASDR, and mortality-related attribution risk factors for COPD across all GBD regions and countries during the period of COVID-19 emergence (2019–2021). However, the results were contrary to our expectations. Globally, there was no significant trend in the ASIR and ASDR of COPD from 2019 to 2021, and the ASMR and ASDR slightly decreased compared to 2019.

Previous studies have shown that COPD patients may face a higher risk of death after contracting COVID-19 due to disruptions in healthcare services and reduced routine management^27^. A meta-analysis by Reyes et al.^28^ found a significantly increased mortality rate in COPD patients infected with COVID-19, and a similar conclusion was reached by Ahn et al.^29^. However, our study found that the COPD disease burden did not show an upward trend from 2019 to 2021. We speculate that this may be related to several factors.

During the COVID-19 pandemic, widespread public health measures such as wearing masks, maintaining social distancing, frequent handwashing, and limiting large gatherings were implemented globally. These measures reduced the circulation of common respiratory viruses, which are important triggers for acute exacerbations of COPD^30^. The reduction in viral infections directly lowered the risk of exacerbations in COPD patients^30-32^. Secondly, statistical and reporting biases may have affected the accurate assessment of the disease burden. In the early stages of the pandemic, some COPD-related deaths were misclassified as COVID-19-related deaths^33^. Additionally, due to the reallocation of medical resources or the impact of public health measures, many COPD patients with mild symptoms did not seek timely medical attention, and some cases were not included in the COPD statistics, which may have led to an underestimation of the disease burden^34^ . On the other hand, although medical resources in some regions were redirected towards COVID-19 patients during the pandemic, the promotion of telemedicine and online consultation services provided new avenues for disease management for COPD patients, helping them continue to receive necessary medical support during the pandemic^35^. Additionally, factors such as the reduction in air pollution and the decline in smoking rates over the past two years may have contributed to a certain extent in reducing the COPD disease burden^36^.

This study further quantified the attributable burden of COPD from the perspective of tobacco-related exposures. Our findings indicate that tobacco exposure remains a major contributor to the global burden of COPD, with active smoking accounting for the dominant share; however, secondhand smoke also imposes a non-negligible burden, underscoring that protecting non-smokers and creating smoke-free environments have independent and essential roles in reducing the overall disease burden^37^. In terms of temporal trends, although the absolute numbers of deaths and DALYs attributable to tobacco increased between 2019 and 2021, the corresponding age-standardized rates declined. The increase in absolute burden may be attributable to global population growth and population ageing, whereas the decline in ASR likely reflects the impact of increasingly stringent tobacco control policies implemented in many countries and regions, which may have partially mitigated the COPD burden attributable to smoking^38^. Consistent with this interpretation, Tashkin et al.^39^ reported that smoking cessation can slow the decline in lung function and delay disease progression among patients with COPD^39^. Sex-stratified analyses further revealed that the burden attributable to active smoking was substantially higher among males, suggesting that smoking cessation interventions targeting high-risk male populations may yield greater marginal benefits. In contrast, the sex differences in ASMR and DALYs attributable to secondhand smoke were markedly smaller than those observed for tobacco and smoking, emphasizing that reducing secondhand smoke exposure relies primarily on institutional and environmental smoke-free policies rather than on individual smoking cessation alone. Moreover, previous studies have suggested that, compared with males, females may be more susceptible to the adverse effects of smoking on the development and prognosis of COPD^16^. Therefore, smoking and secondhand smoke exposure among women should also receive greater attention.

### Limitations

This study has several limitations. First, we analyzed COPD burden based on the GBD 2021 dataset for the first two years after the COVID-19 outbreak, which may not capture the latest trends or long-term consequences, potentially underestimating the pandemic’s impact. Second, the data in the GBD database come from various sources, including national and regional reports, hospital data, and mortality statistics. The quality and coverage of these data may vary across different regions due to differences in healthcare infrastructure, diagnostic standards, reporting systems, and other factors, despite quality control, sparse or poor reporting may affect accuracy and comparability. Third, the EAPC estimates assume linear trends, which may not fully reflect nonlinear changes. Fourth, misclassification between COPD and COVID-19 deaths during 2020–2021 may have biased mortality estimates. Fifth, as an ecological study, our findings cannot be extrapolated to individuals, and the lack of stratification by COPD severity limits the assessment of disease heterogeneity. These shortcomings may affect the comprehensiveness and accuracy of the study results.

## CONCLUSIONS

Our study found that the outbreak of COVID-19 does not appear to have altered the trend of COPD disease burden changes, to more accurately assess the specific effects of the pandemic on the COPD disease burden, more research should be conducted in the future. These studies should not only focus on the changes in the disease burden of COPD patients during the pandemic but also explore the long-term impact of COVID-19 on the disease burden in COPD patients. Only then can we truly understand the far-reaching effects of COVID-19 on the COPD disease burden and provide scientific evidence for the optimization of relevant prevention and control strategies. Meanwhile, in the context of the COVID-19 pandemic, smoking and tobacco exposure remain important risk factors contributing to the burden of COPD, underscoring the urgent need for heightened attention from the public and relevant authorities.

## Supplementary Material



## Data Availability

Data sharing is not applicable to this article as no new data were created.

## References

[1] Global Initiative for Chronic Obstructive Lung Disease. 2024 GOLD report: Global strategy for prevention, diagnosis and management of COPD: 2024 report. Accessed April 24, 2026. https://goldcopd.org/2024-gold-report/

[2] GBD 2021 Diseases and Injuries Collaborators. Global incidence, prevalence, years lived with disability (YLDs), disability-adjusted life-years (DALYs), and healthy life expectancy (HALE) for 371 diseases and injuries in 204 countries and territories and 811 subnational locations, 1990-2021: A systematic analysis for the Global Burden of Disease Study 2021. Lancet. 2024;403(10440):2133-2161. doi:10.1016/S0140-6736(24)00757-8 38642570 PMC11122111

[3] Li M, Hanxiang C, Na Z, et al. Burden of COPD in China and the global from 1990 to 2019: A systematic analysis for the Global Burden of Disease Study 2019. BMJ Open Respir Res. 2023;10(1):e001698. doi:10.1136/bmjresp-2023-001698 PMC10347506 37438048

[4] Richie RC. Morbidity and mortality associated with Chronic Obstructive Pulmonary Disease (COPD). J Insur Med. 2023;49(4):230-243. doi:10.17849/insm-49-04-230-243.1 37074878

[5] Safiri S, Carson-Chahhoud K, Noori M, et al. Burden of chronic obstructive pulmonary disease and its attributable risk factors in 204 countries and territories, 1990-2019: Results from the Global Burden of Disease Study 2019. BMJ. 2022;378:e069679. doi:10.1136/bmj-2021-069679 35896191 PMC9326843

[6] World Health Organization. The top 10 causes of death 2020. Accessed April 24, 2026. https://www.who.int/news-room/fact-sheets/detail/the-top-10-causes-of-death/

[7] Institute for Health Metrics and Evaluation. Global Burden of Disease Study 2021 (GBD 2021) Data Resources. Accessed April 24, 2026. https://ghdx.healthdata.org/gbd-2021

[8] Chen X, Zhou CW, Fu YY, et al. Global, regional, and national burden of chronic respiratory diseases and associated risk factors, 1990-2019: Results from the Global Burden of Disease Study 2019. Front Med (Lausanne). 2023;10:1066804. doi:10.3389/fmed.2023.1066804 37056726 PMC10088372

[9] Li H, Liang H, Wei L, et al. Health Inequality in the Global Burden of Chronic Obstructive Pulmonary Disease: Findings from the Global Burden of Disease Study 2019. Int J Chron Obstruct Pulmon Dis. 2022;17:1695-1702. doi:10.2147/COPD.S369120 35923358 PMC9342709

[10] Xu J, Ji Z, Zhang P, Chen T, Xie Y, Li J. Disease burden of COPD in the Chinese population: A systematic review. Ther Adv Respir Dis. 2023;17:17534666231218899. doi:10.1177/17534666231218899 38146618 PMC10752056

[11] Bridevaux PO, Gerbase MW, Probst-Hensch NM, Schindler C, Gaspoz JM, Rochat T. Long-term decline in lung function, utilisation of care and quality of life in modified GOLD stage 1 COPD. Thorax. 2008;63(9):768-774. doi:10.1136/thx.2007.093724 18505800

[12] Singh D, D’Urzo AD, Donohue JF, Kerwin EM. Weighing the evidence for pharmacological treatment interventions in mild COPD; A narrative perspective. Respir Res. 2019;20(1):141. doi:10.1186/s12931-019-1108-9 31286970 PMC6615221

[13] Calverley PMA. COPD in the time of COVID-19. EClinicalMedicine. 2021;34:100832. doi:10.1016/j.eclinm.2021.100832 33880440 PMC8049609

[14] Gerayeli FV, Milne S, Cheung C, et al. COPD and the risk of poor outcomes in COVID-19: A systematic review and meta-analysis. EClinicalMedicine. 2021;33:100789. doi:10.1016/j.eclinm.2021.100789 33758801 PMC7971471

[15] World Health Organization. Coronavirus disease (COVID-19) Epidemiological Updates and Monthly Operational Updates. Accessed April 24, 2026. https://www.who.int/emergencies/diseases/novel-coronavirus-2019/situation-reports

[16] Çolak Y, Nordestgaard BG, Lange P, Afzal S. Sex differences in COPD in relation to smoking exposure: A population-based cohort study. Thorax. 2025;80(8):512-519. doi:10.1136/thorax-2024-222682 40185636

[17] Çolak Y, Løkke A, Marott JL, et al. Low smoking exposure and development and prognosis of COPD over four decades: A population-based cohort study. Eur Respir J. 2024;64(3):2400314. doi:10.1183/13993003.00314-2024 38936967

[18] Tamimi A, Serdarevic D, Hanania NA. The effects of cigarette smoke on airway inflammation in asthma and COPD: Therapeutic implications. Respir Med. 2012;106(3):319-328. doi:10.1016/j.rmed.2011.11.003 22196881

[19] Osman LM, Douglas JG, Garden C, et al. Indoor air quality in homes of patients with chronic obstructive pulmonary disease. Am J Respir Crit Care Med. 2007;176(5):465-472. doi:10.1164/rccm.200605-589OC 17507547

[20] Institute for Health Metrics and Evaluation. Global Burden of Disease (GBD) data and tools guide. Accessed April 24, 2026. https://www.healthdata.org/research-analysis/about-gbd/gbd-data-and-tools-guide

[21] Vandenbroucke JP, von Elm E, Altman DG, et al. Strengthening the Reporting of Observational Studies in Epidemiology (STROBE): Explanation and elaboration. Int J Surg. 2014;12(12):1500-1524. doi:10.1016/j.ijsu.2014.07.014 25046751

[22] Zhang K, Kan C, Han F, et al. Global, regional, and national epidemiology of diabetes in children from 1990 to 2019. JAMA Pediatr. 2023;177(8):837-846. doi:10.1001/jamapediatrics.2023.2029 37399036 PMC10318549

[23] GBD 2017 Disease and Injury Incidence and Prevalence Collaborators. Global, regional, and national incidence, prevalence, and years lived with disability for 354 diseases and injuries for 195 countries and territories, 1990-2017: A systematic analysis for the Global Burden of Disease Study 2017. Lancet. 2018;392(10159):1789-1858. doi:10.1016/S0140-6736(18)32279-7 30496104 PMC6227754

[24] Wang LY, Wang WF, Hui SY, Yang L, Liu YX, Li HJ. Emerging epidemiological trends of multiple sclerosis among adults aged 20-54 years, 1990-2021, with projections to 2035: A systematic analysis for the global burden of disease study 2021. Front Neurol. 2025;16:1616245. doi:10.3389/fneur.2025.1616245 40708950 PMC12286822

[25] Wang Y, Wang K, Cheng W, Zhang Y. Global burden of chronic obstructive pulmonary disease attributable to ambient ozone in 204 countries and territories during 1990-2019. Environ Sci Pollut Res Int. 2022;29(6):9293-9305. doi:10.1007/s11356-021-16233-y 34505240

[26] Gutiérrez Villegas C, Paz-Zulueta M, Herrero-Montes M, Parás-Bravo P, Madrazo Pérez M. Cost analysis of chronic obstructive pulmonary disease (COPD): A systematic review. Health Econ Rev. 2021;11(1):31. doi:10.1186/s13561-021-00329-9 34403023 PMC8369716

[27] Lam GY, Wen C, Ronksley PE, et al. Impact of COVID-19 pandemic on chronic obstructive pulmonary disease healthcare use, exacerbations, and mortality: A population study. Ann Am Thorac Soc. 2024;21(9):1281-1288. doi:10.1513/AnnalsATS.202312-1078OC 38820253 PMC11376357

[28] Reyes FM, Hache-Marliere M, Karamanis D, et al. Assessment of the association of COPD and asthma with in-hospital mortality in patients with COVID-19. A systematic review, meta-analysis, and meta-regression analysis. J Clin Med. 2021;10(10):2087. doi:10.3390/jcm10102087 34068023 PMC8152460

[29] Ahn C, Park Y. Chronic obstructive pulmonary disease mortality and hospitalization during the COVID-19 pandemic compared with before the pandemic: A systematic review and meta-analysis. J Pers Med. 2024;14(3):296. doi:10.3390/jpm14030296 38541038 PMC10970825

[30] Tan JY, Conceicao EP, Wee LE, Sim XYJ, Venkatachalam I. COVID-19 public health measures: A reduction in hospital admissions for COPD exacerbations. Thorax. 2021;76(5):512-513. doi:10.1136/thoraxjnl-2020-216083 33273024

[31] Chan KPF, Ma TF, Kwok WC, et al. Significant reduction in hospital admissions for acute exacerbation of chronic obstructive pulmonary disease in Hong Kong during coronavirus disease 2019 pandemic. Respir Med. 2020;171:106085. doi:10.1016/j.rmed.2020.106085 32917356 PMC7354382

[32] So JY, O’Hara NN, Kenaa B, et al. Population decline in COPD admissions during the COVID-19 pandemic associated with lower burden of community respiratory viral infections. Am J Med. 2021;134(10):1252-1259.e3. doi:10.1016/j.amjmed.2021.05.008 34126098 PMC8196237

[33] Fedeli U, Casotto V, Barbiellini Amidei C, Vianello A, Guarnieri G. COPD-related mortality before and after mass COVID-19 vaccination in Northern Italy. Vaccines (Basel). 2023;11(8):1392. doi:10.3390/vaccines11081392 37631960 PMC10459975

[34] Toppen W, Yan P, Markovic D, et al. Chronic obstructive pulmonary disease is not associated with in-hospital mortality in COVID-19: An observational cohort analysis. Int J Chron Obstruct Pulmon Dis. 2022;17:3111-3121. doi:10.2147/COPD.S386463 36570857 PMC9788836

[35] Lin X, Duan G, Huang J, et al. Construction of a smart hospital innovation platform using the internet + technology. Altern Ther Health Med. 2024;30(12):495-505. Accessed April 24, 2026. https://pubmed.ncbi.nlm.nih.gov/38639608/38639608

[36] Ko FWS, Lau LHS, Ng SS, et al. Respiratory admissions before and during the COVID-19 pandemic with mediation analysis of air pollutants, mask-wearing and influenza rates. Respirology. 2023;28(1):47-55. doi:10.1111/resp.14345 36065624 PMC9538077

[37] Putcha N, Barr RG, Han MK, et al. Understanding the impact of second-hand smoke exposure on clinical outcomes in participants with COPD in the SPIROMICS cohort. Thorax. 2016;71:411-420. doi:10.1136/thoraxjnl-2015-207487 26962015 PMC5235992

[38] U.S. Department of Health and Human Services. Smoking Cessation: A Report of the Surgeon General. U.S. Department of Health and Human Services, Centers for Disease Control and Prevention, National Center for Chronic Disease Prevention and Health Promotion, Office on Smoking and Health; 2020. Accessed April 24, 2026. https://www.ncbi.nlm.nih.gov/books/NBK555591/pdf/Bookshelf_NBK555591.pdf

[39] Tashkin DP. Smoking cessation in chronic obstructive pulmonary disease. Semin Respir Crit Care Med. 2015;36(4):491-507. doi:10.1055/s-0035-1555610 26238637

