# Supplementary material for: The contributions of active and passive smoking to COPD-related mortality and DALYs in the context of COVID-19: Global Burden 2019–2021
Source: Tob Induc Dis. 2026 Jun 10;24:10.18332/tid/221062. doi: 10.18332/tid/221062 (PMC13256146; doi:10.18332/tid/221062)
Supplement: Supplementary file 1 [file TID-24-87-s1.pdf]

Supplementary Figures

Figure S1 Trends of ASIR in SDI regions from 2019 to 2021. ASIR, age-standardized incidence rate; SDI, Socio-demographic Index.

Figure S2 Trends of ASPR in SDI regions from 2019 to 2021. ASPR, age-standardized prevalence rate; SDI, Socio-demographic Index.

Figure S3 Trends of ASDR in SDI regions from 2019 to 2021. ASDR, age-standardized DALYs rate; SDI, Socio-demographic Index; DALYs, disability-adjusted life years.

Figure S4 The number of incident cases and ASIR in different geographic regions in 2019 and 2021. ASIR, age-standardized incidence rate.

Figure S5 The number of prevalence cases and ASPR in different geographic regions in 2019 and 2021. ASPR, age-standardized prevalence rate.

Figure S6 The number of DALYs cases and ASDR in different geographic regions in 2019 and 2021. DALYs, disability-adjusted life years; ASDR, age-standardized DALYs rate.

Figure S7 Global ASIR of COPD in 2019 (A) and 2021(B). ASIR, age-standardized incidence rate, COPD: chronic obstructive pulmonary disease.

Figure S8 Global ASPR of COPD in 2019 (A) and 2021(B). ASPR, age-standardized prevalence rate, COPD: chronic obstructive pulmonary disease.

Figure S9 Global ASDR of COPD in 2019 (A) and 2021(B). ASDR, age-standardized DALYs rate; DALYs, disability-adjusted life years; COPD: chronic obstructive pulmonary disease.

Figure S10 The number of incident cases (bar plot) and ASIR(line plot) of COPD, stratified by age and sex in 2021. The blank vertical lines indicate the prevalent case with 95% uncertainty intervals for men and women. ASIR, age-standardized incidence rate; COPD: chronic obstructive pulmonary disease.

Figure S11 The number of prevalence cases (bar plot) and ASPR(line plot) of COPD, stratified by age and sex in 2021. The blank vertical lines indicate the prevalent case with 95% uncertainty intervals for men and women. ASPR, age-standardized prevalence rate; COPD: chronic obstructive pulmonary disease.

Figure S12 The number of DALYs cases (bar plot) and ASDR(line plot) of COPD, stratified by age and sex in 2021. The blank vertical lines indicate the prevalent case with 95% uncertainty intervals for men and women. ASDR, age-standardized DALYs rate; DALYs, disability-adjusted life years; COPD: chronic obstructive pulmonary disease.

Figure S13 ASMR of COPD in 204 countries by SDI. ASMR, age-standardized mortality rate; COPD, Chronic Obstructive Pulmonary Disease; SDI, socio-demographic index.

Figure S14 ASDR of COPD in 204 countries by SDI. ASDR, age-standardized DALYs rate; COPD, Chronic Obstructive Pulmonary Disease; SDI, socio-demographic index.

Figure S15 Global risk factors contributing to COPD-related mortality rates.

Figure S16 Age-standardized rates (ASRs) attributable to tobacco, smoking, and secondhand smoke globally and across five SDI regions in 2019 and 2021: (A) ASMR in 2019; (B) ASMR in 2021; (C) ASDR in 2019; and (D) ASDR in 2021. SDI: Socio-demographic Index; ASMR: age-standardized mortality rate; ASDR: age-standardized disability-adjusted life years.

Figure S17 Deaths(A) and DALYs(B) number attributable to tobacco, smoking, and secondhand smoke by sex. DALYs: disability-adjusted life years.

Supplementary Tables

Table S1 The number of incident cases and ASIR of COPD between 2019 and 2021 at the global and regional levels. ASIR, age-standardized incidence rate, COPD: chronic obstructive pulmonary disease.

Table S2 The number of prevalence cases and ASPR of COPD between 2019 and 2021 at the global and regional levels. ASPR, age-standardized prevalence rate, COPD: chronic obstructive pulmonary disease.

Table S3 The number of DALYs cases and ASPR of COPD between 2019 and 2021 at the global and regional levels. DALYs, disability-adjusted life years; ASDR, age-standardized DALYs rate, COPD: chronic obstructive pulmonary disease.

Table S4 The number of incident cases and ASIR of COPD between 2019 and 2021 at the national level. ASIR, age-standardized incidence rate, COPD: chronic obstructive pulmonary disease.

Table S5 The number of prevalence cases and ASPR of COPD between 2019 and 2021 at the national level. ASPR, age-standardized prevalence rate, COPD: chronic obstructive pulmonary disease.

Table S6 The number of mortality cases and ASMR of COPD between 2019 and 2021 at the national level. ASMR, age-standardized mortality rate, COPD: chronic obstructive pulmonary disease.

Table S7 The number of DALYs cases and ASDR of COPD between 2019 and 2021 at the national level. DALYs, disability-adjusted life years; ASDR, age-standardized DALYs rate, COPD:

chronic obstructive pulmonary disease.

STROBE Checklist

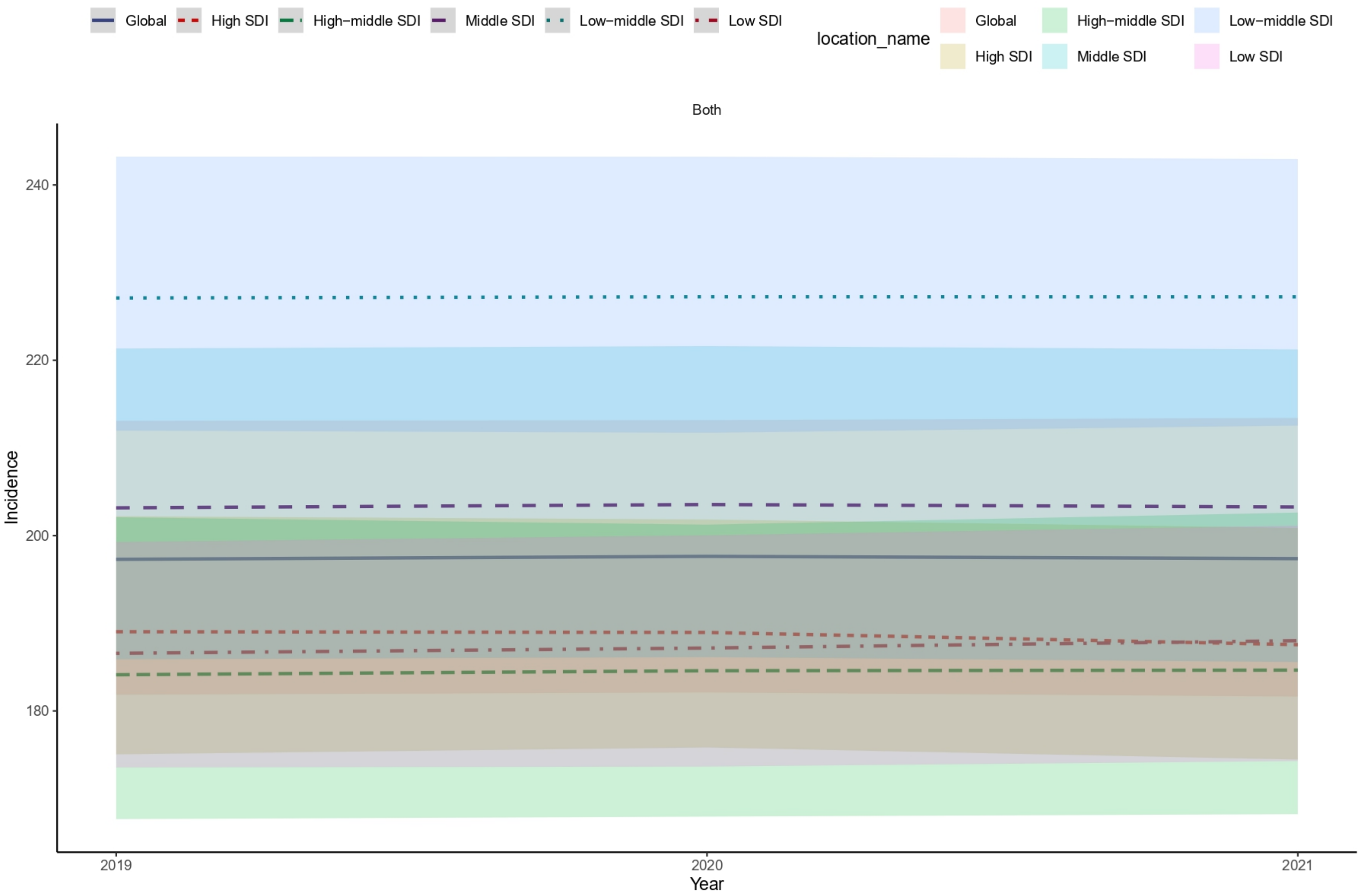

Figure S1 Trends of ASIR in SDI regions from 2019 to 2021. ASIR, age-standardized incidence rate; SDI, Socio-demographic Index.

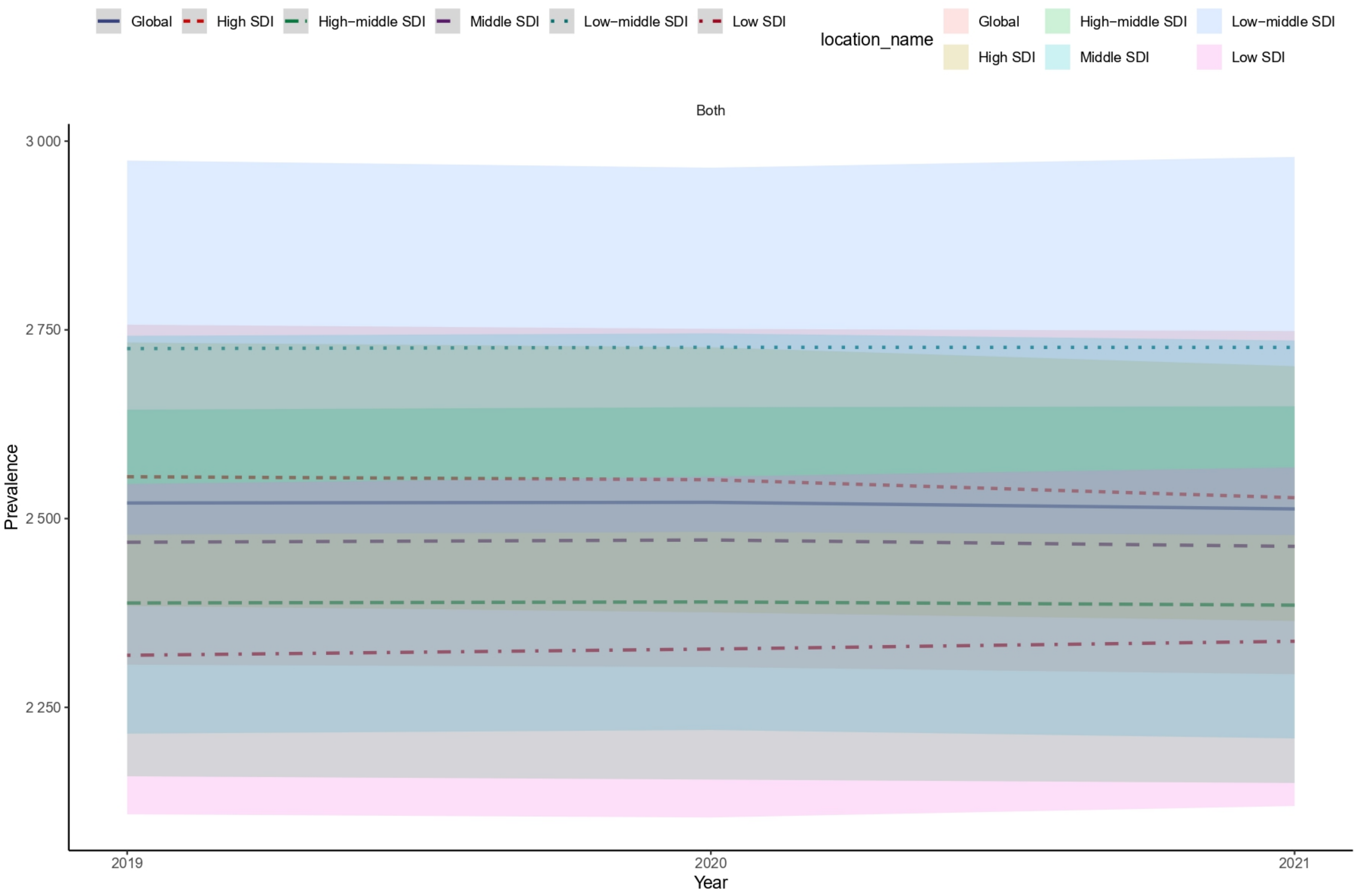

Figure S2 Trends of ASPR in SDI regions from 2019 to 2021. ASPR, age-standardized prevalence rate; SDI, Socio-demographic Index.

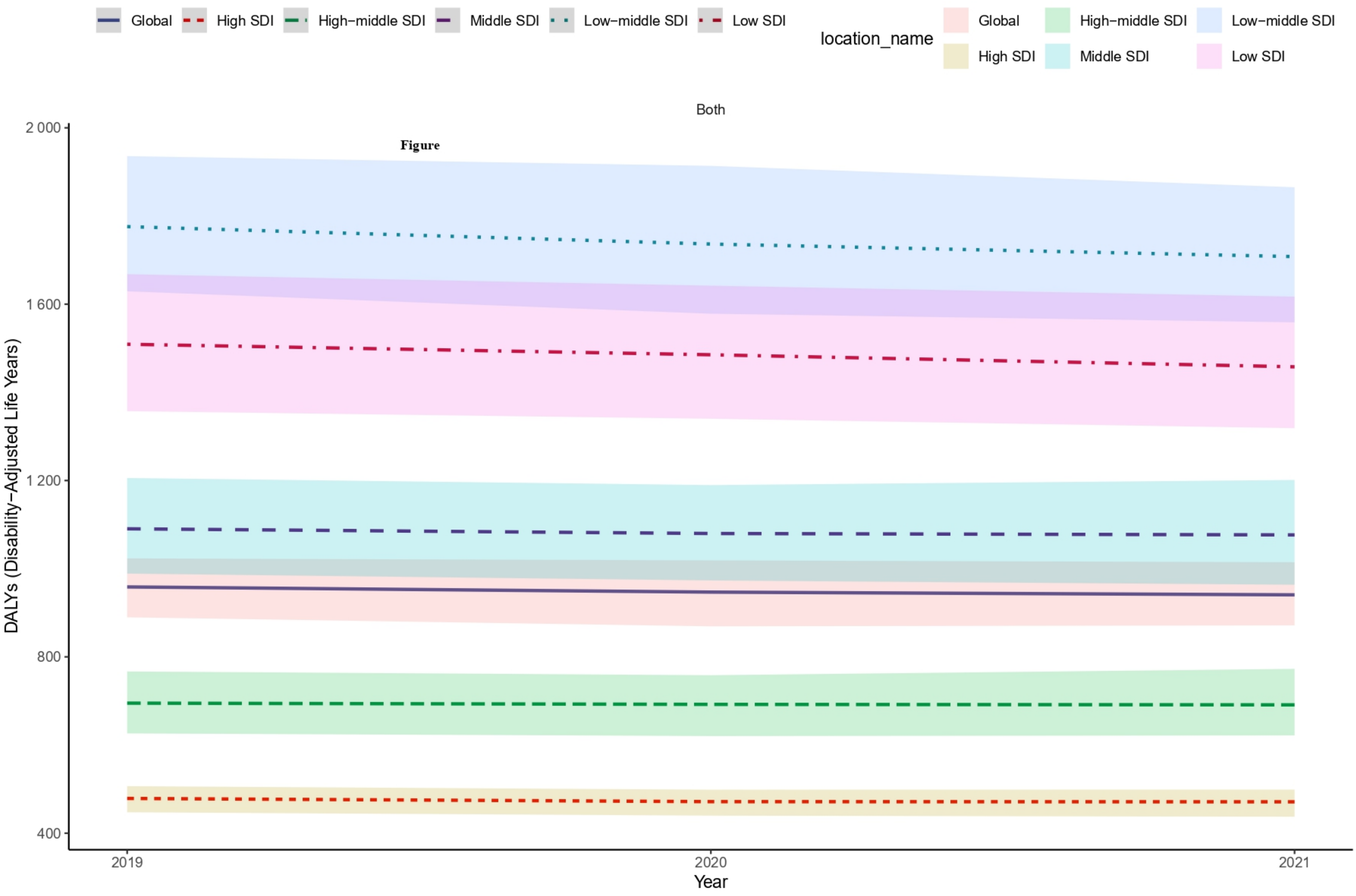

Figure S3 Trends of ASDR in SDI regions from 2019 to 2021. ASDR, age-standardized DALYs rate; SDI, Socio-demographic Index; DALYs, disability-adjusted life years.

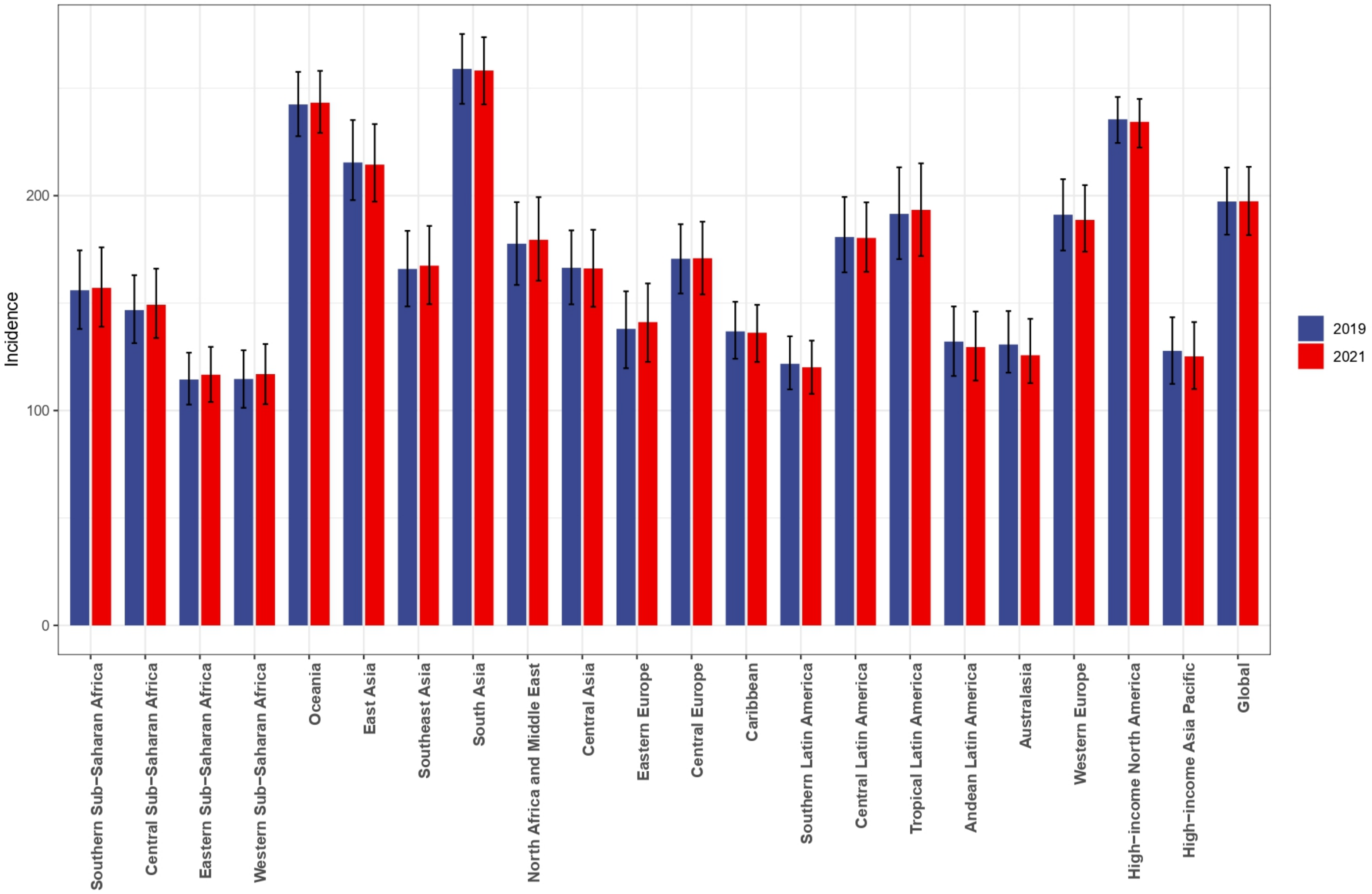

Figure S4 The number of incident cases and ASIR in different geographic regions in 2019 and 2021. ASIR, age-standardized incidence rate.

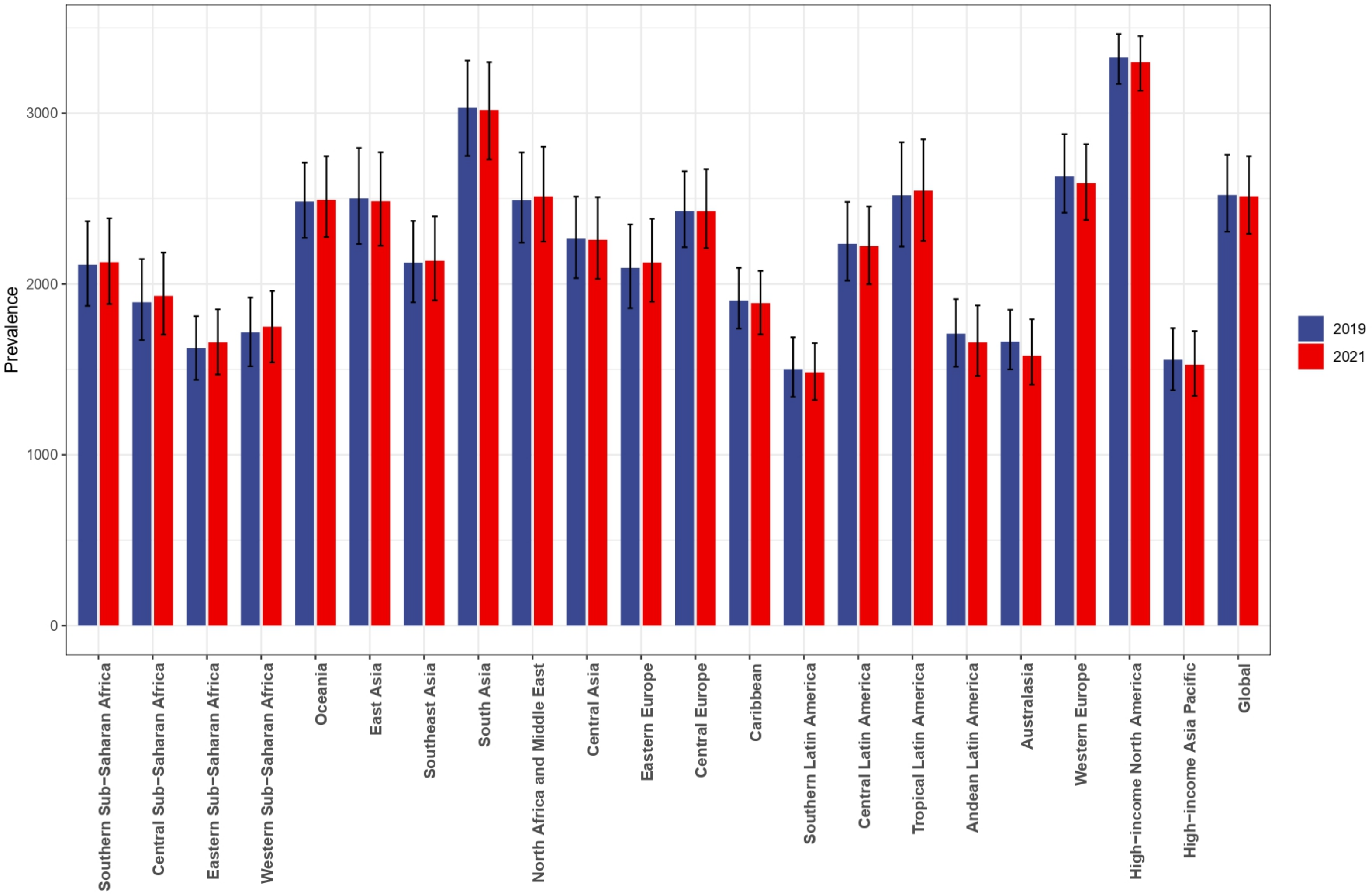

Figure S5 The number of prevalence cases and ASPR in different geographic regions in 2019 and 2021. ASPR, age-standardized prevalence rate.

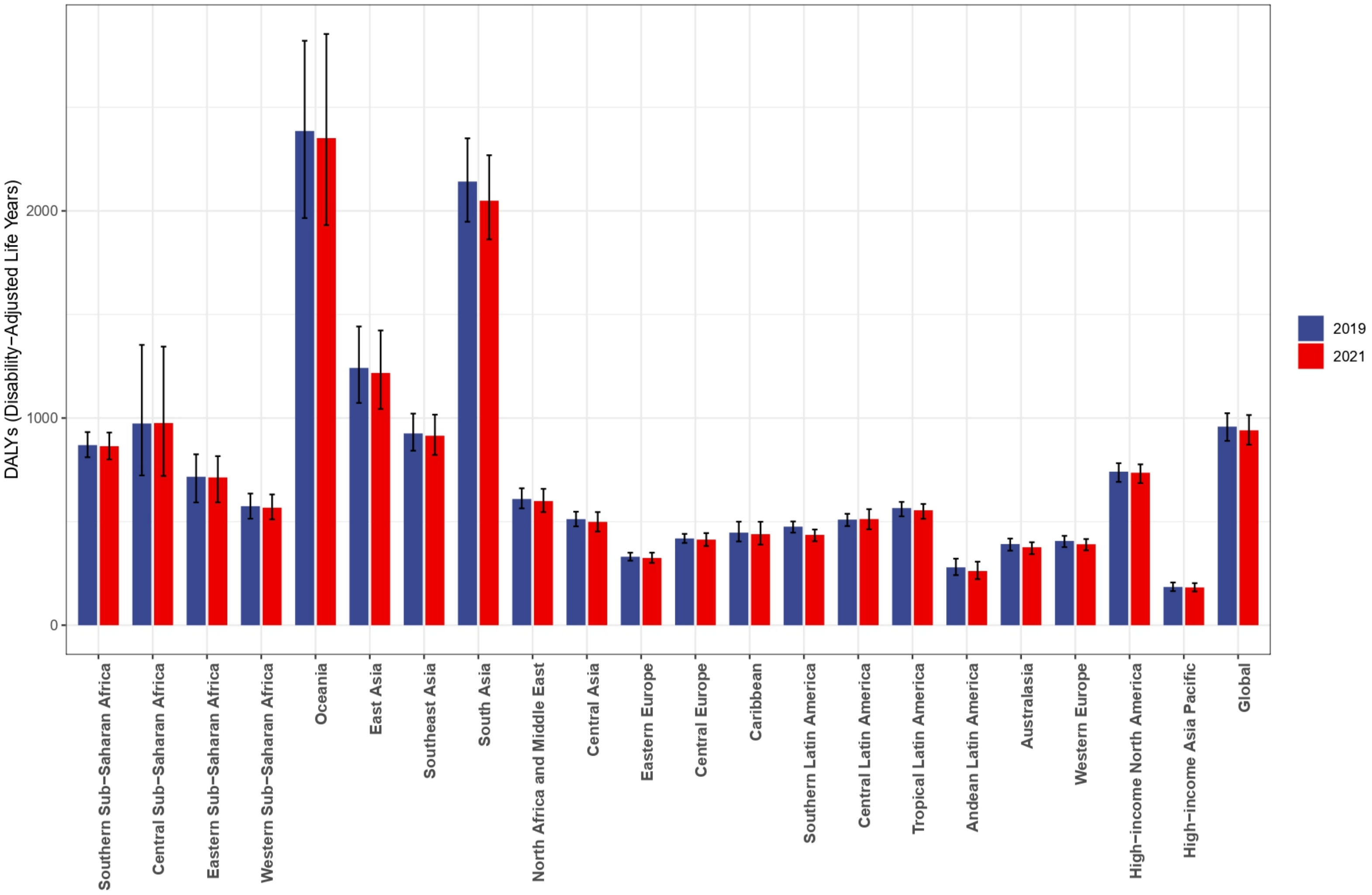

Figure S6 The number of DALYs cases and ASDR in different geographic regions in 2019 and 2021. DALYs, disability-adjusted life years; ASDR, age-standardized DALYs rate.

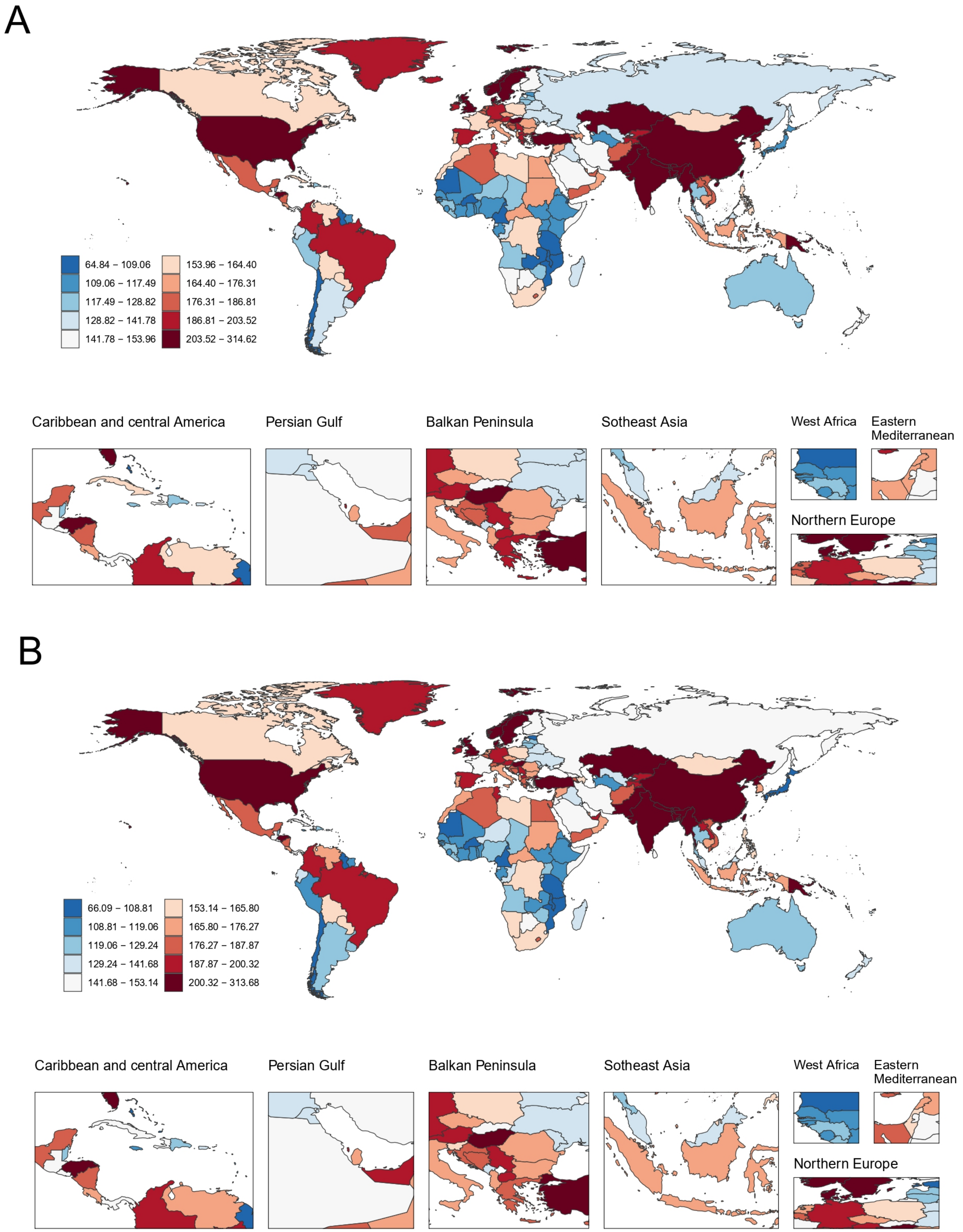

Figure S7 Global ASIR of COPD in 2019 (A) and 2021(B). ASIR, age-standardized incidence rate, COPD, chronic obstructive pulmonary disease.

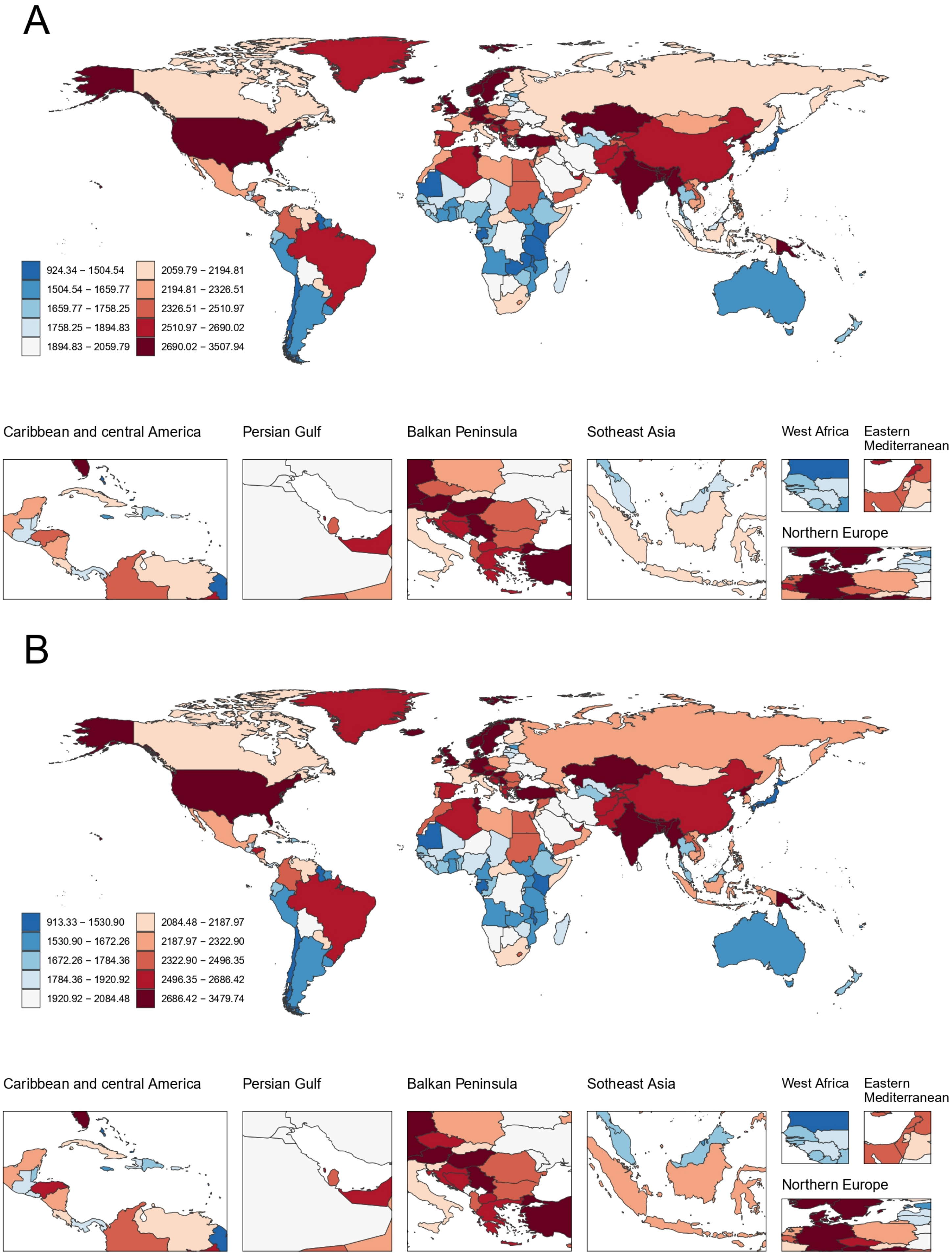

Figure S8 Global ASPR of COPD in 2019 (A) and 2021(B). ASPR, age-standardized prevalence rate, COPD, chronic obstructive pulmonary disease.

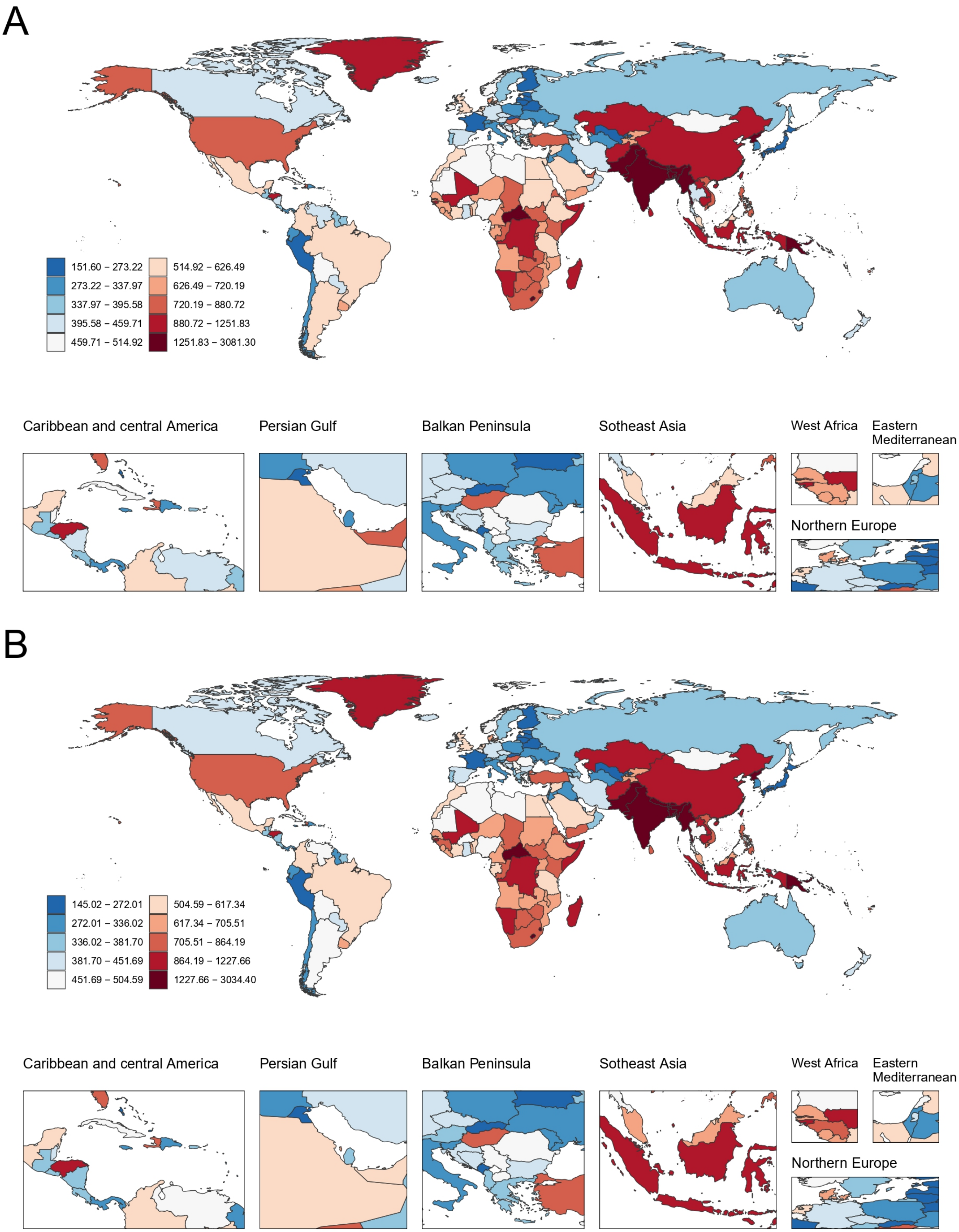

Figure S9 Global ASDR of COPD in 2019 (A) and 2021(B). ASDR, age-standardized DALYs rate; DALYs, disability-adjusted life years; COPD, chronic obstructive pulmonary disease.

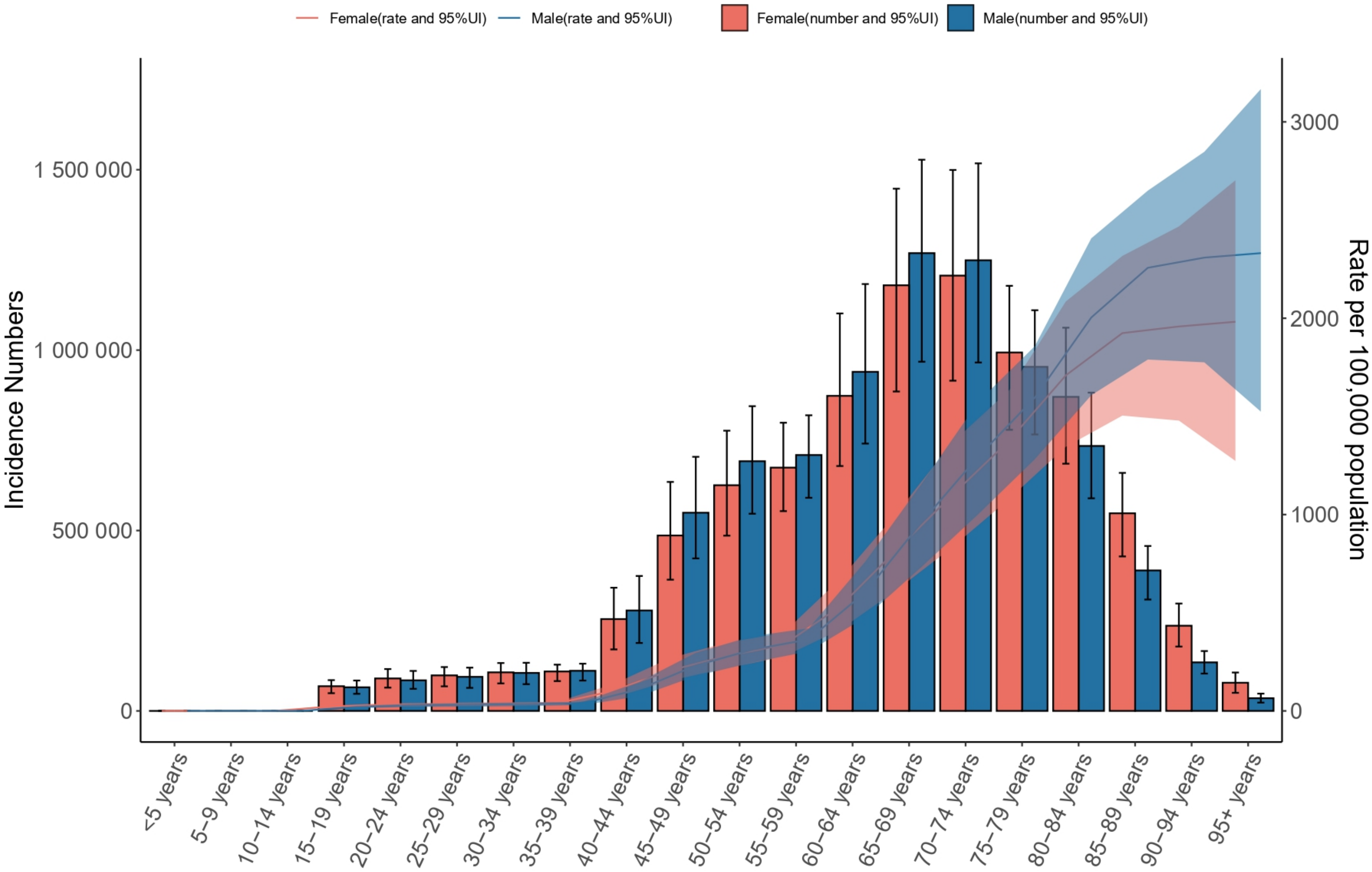

Figure S10 The number of incident cases (bar plot) and ASIR(line plot) of COPD, stratified by age and sex in 2021. The blank vertical lines indicate the prevalent case with 95% uncertainty intervals for men and women. ASIR, age-standardized incidence rate; COPD, Chronic Obstructive Pulmonary Disease.

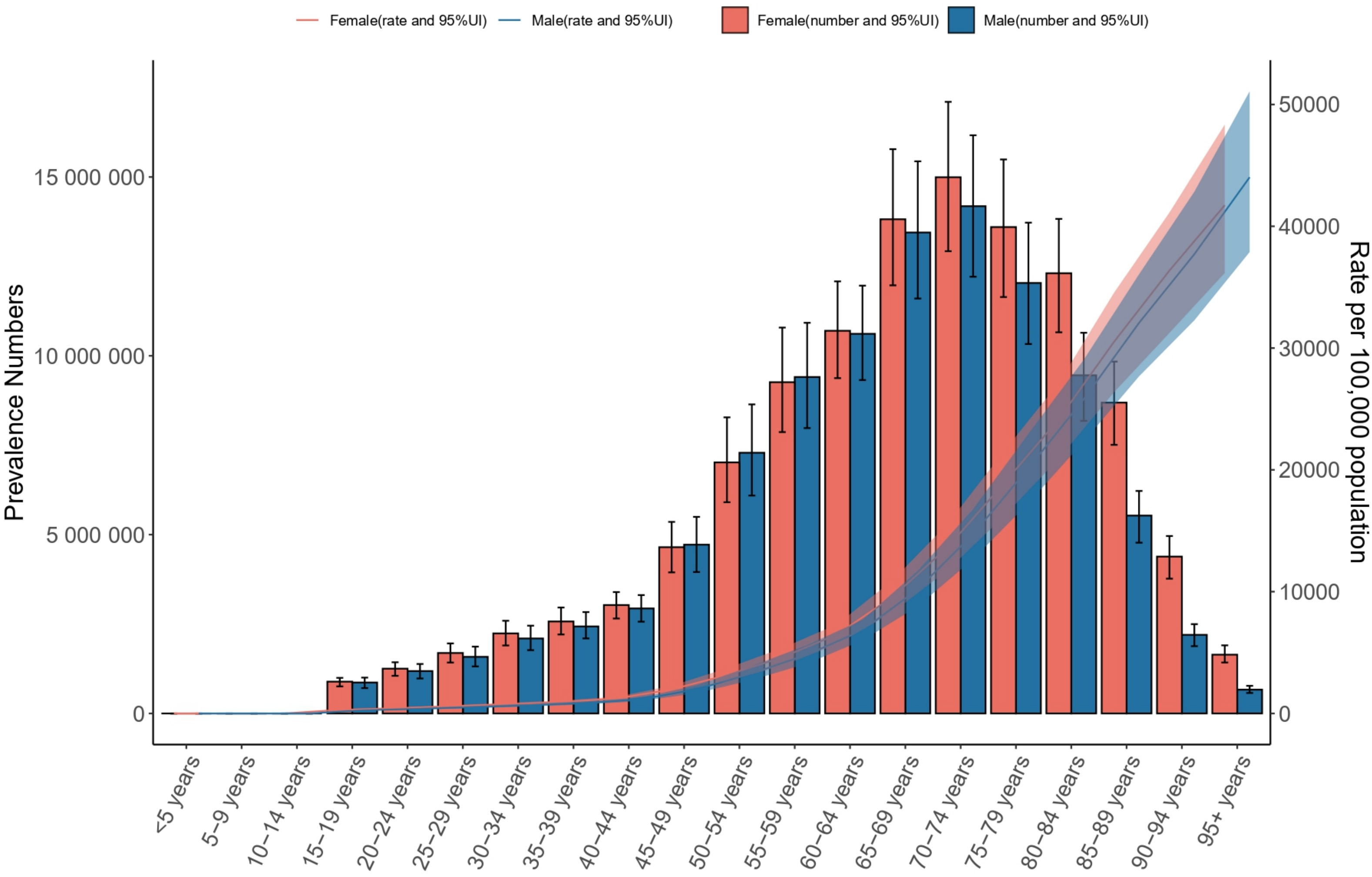

Figure S11 The number of prevalence cases (bar plot) and ASPR(line plot) of COPD, stratified by age and sex in 2021. The blank vertical lines indicate the prevalent case with 95% uncertainty intervals for men and women. ASPR, age-standardized prevalence rate; COPD, chronic obstructive pulmonary disease.

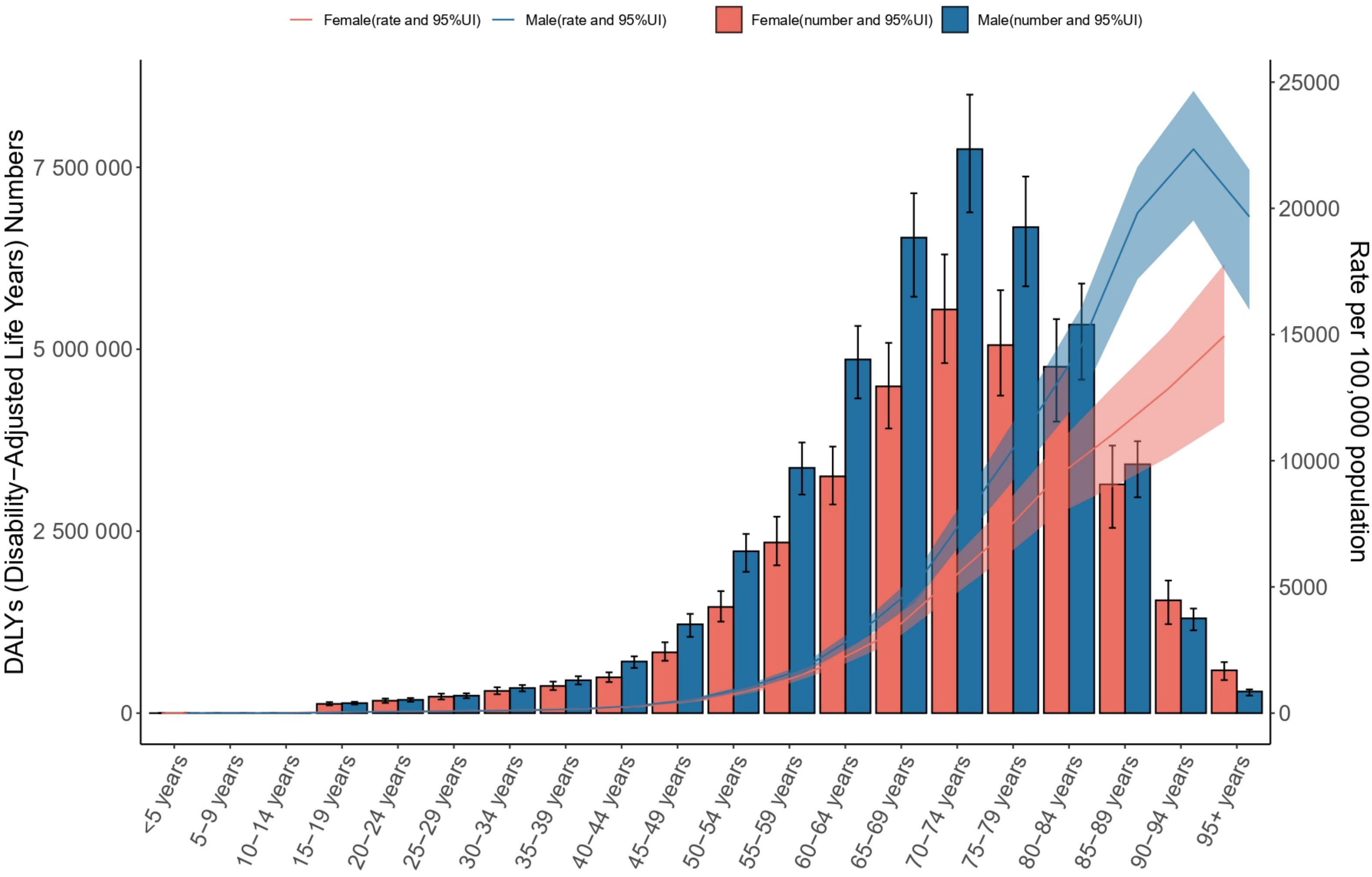

Figure S12 The number of DALYs cases (bar plot) and ASDR(line plot) of COPD, stratified by age and sex in 2021. The blank vertical lines indicate the prevalent case with 95% uncertainty intervals for men and women. ASDR, age-standardized DALYs rate; DALYs, disability-adjusted life years; COPD: chronic obstructive pulmonary disease.

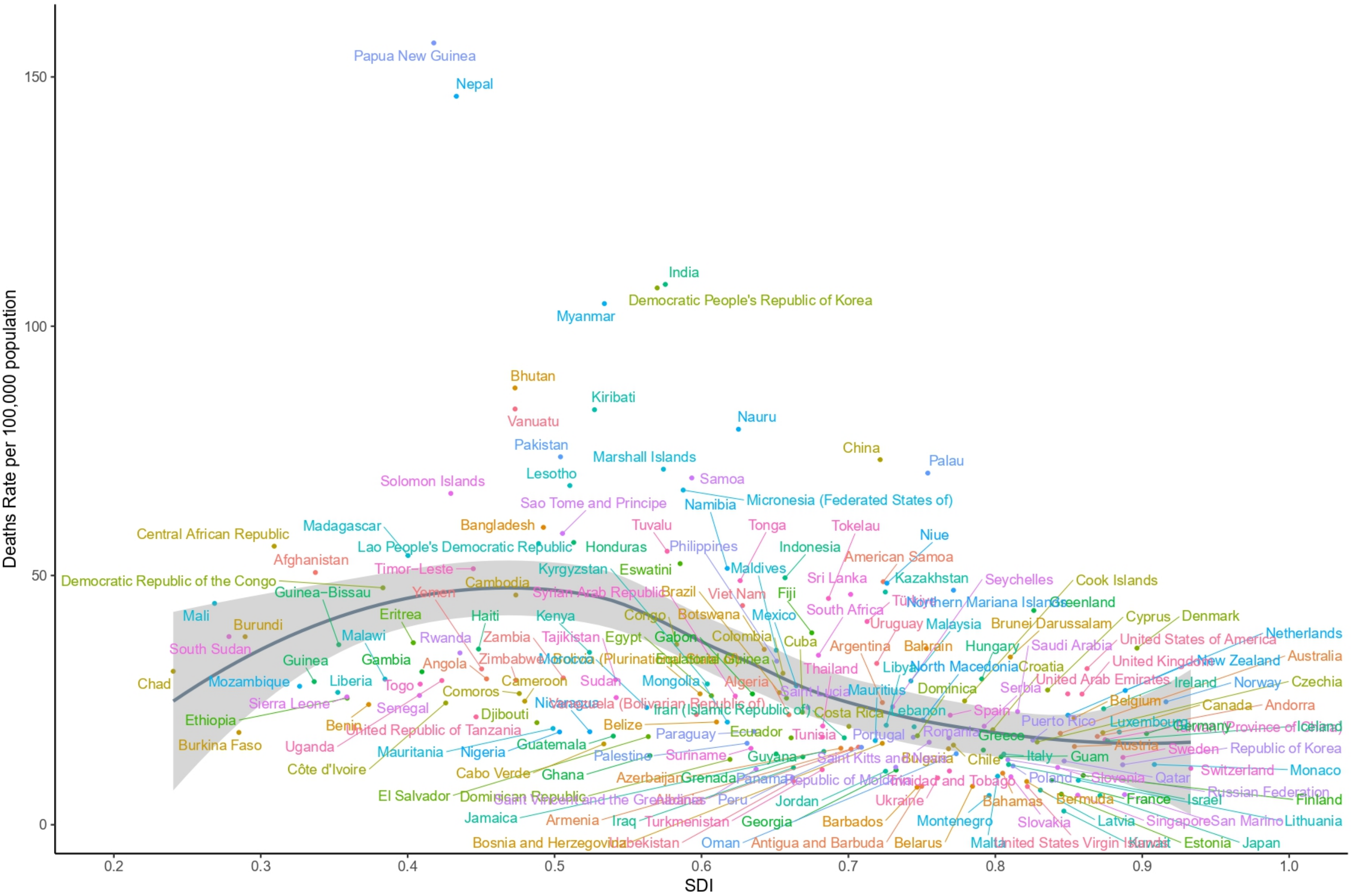

Figure S13 ASMR of COPD in 204 countries by SDI. ASMR, age-standardized mortality rate; COPD, Chronic Obstructive Pulmonary Disease; SDI, socio-demographic index.

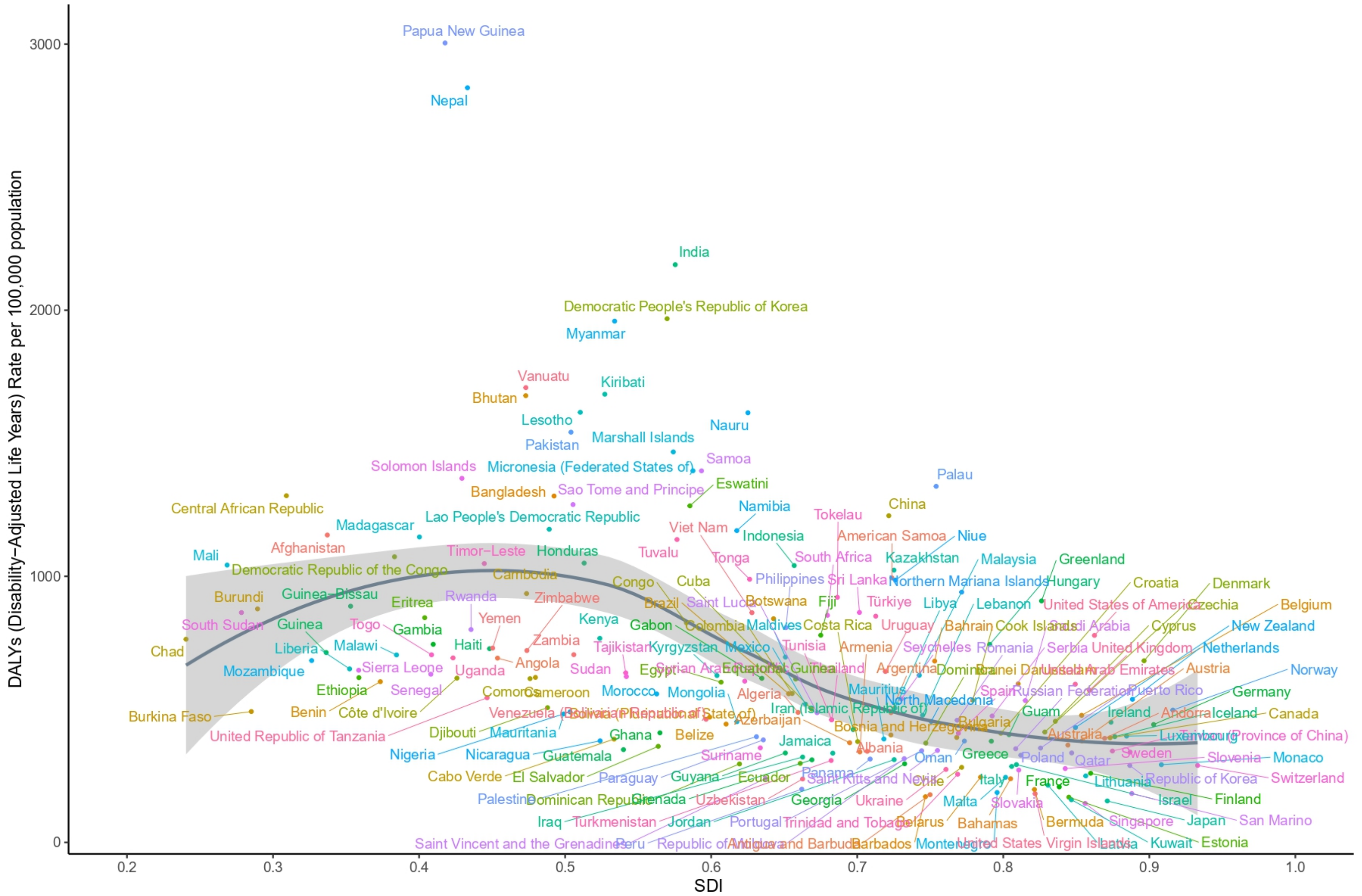

Figure S14 ASDR of COPD in 204 countries by SDI. ASDR, age-standardized DALYs rate; COPD, Chronic Obstructive Pulmonary Disease; SDI, socio-demographic index.

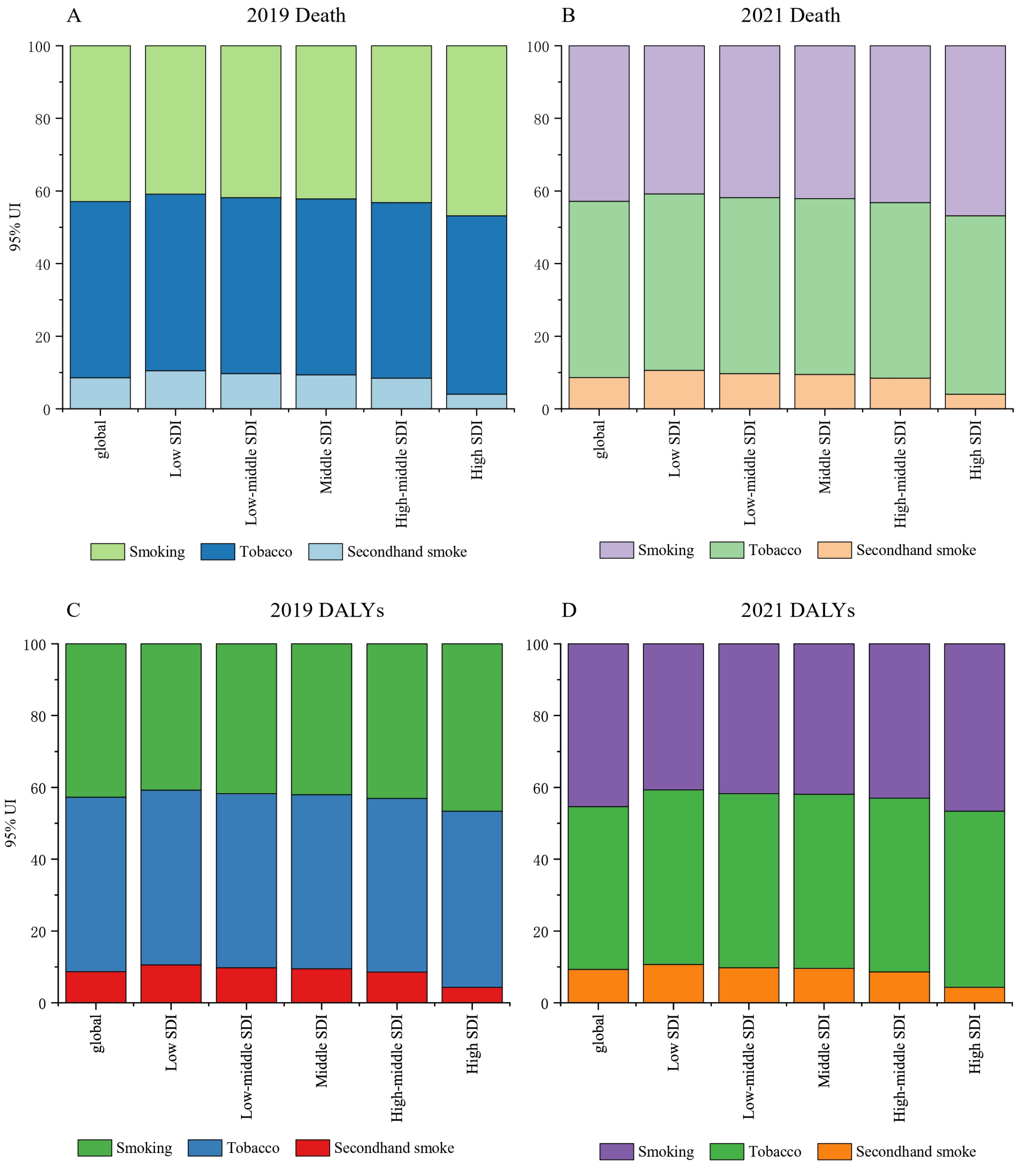
A
2019 Death
B
2021 Death
C
2019 DALYs
D
2021 DALYs
95% UI
0
20
40
60
80
100
global
Low SDI
Low-middle SDI
Middle SDI
High-middle SDI
High SDI
Smoking
Tobacco
Secondhand smoke

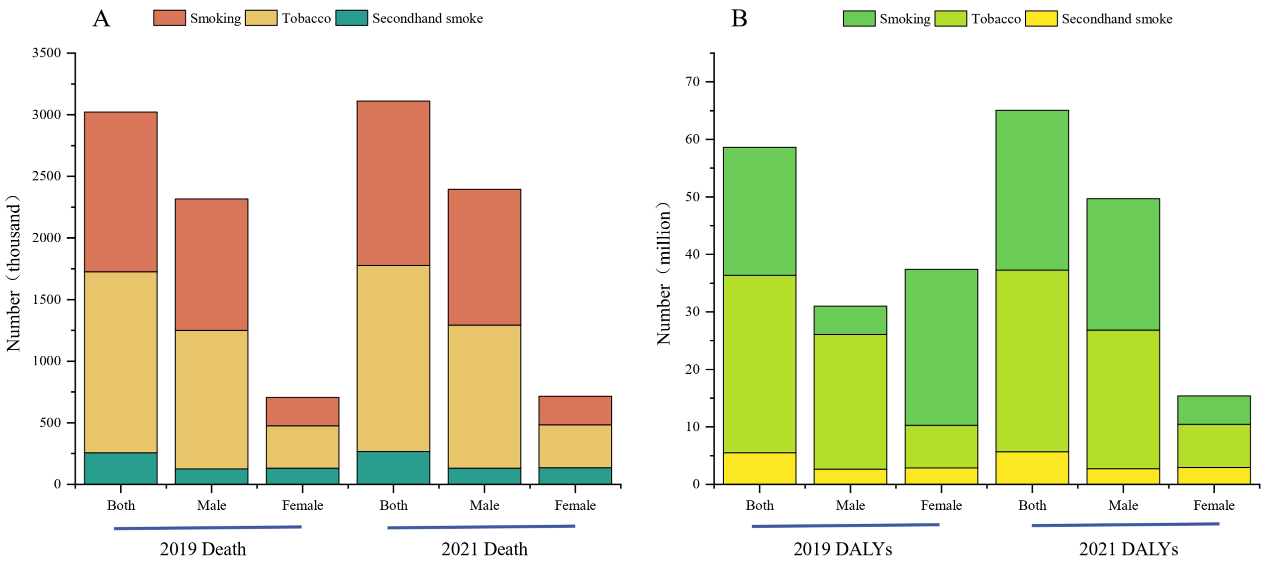
A
Smoking
Tobacco
Secondhand smoke
Number（thousand）
0
500
1000
1500
2000
2500
3000
3500
Both
Male
Female
Both
Male
Female
2019 Death
2021 Death
B
Smoking
Tobacco
Secondhand smoke
Number（million）
0
10
20
30
40
50
60
70
Both
Male
Female
Both
Male
Female
2019 DALYs
2021 DALYs

Table S1 The number of incident cases and ASIR of COPD between 2019 and 2021 at the global and regional levels

	2019		2021		
**Location**	**Number**	**ASIR**	**Number**	**ASIR**	**EAPCs**
Global	16126484.84(14787209.81,17514355.12)	197.30(181.85,213.09)	16895444.79(15471347.31,18335690.52)	197.37(181.65,213.42)	0.02(-1.08,1.14)
High SDI	3634851.46(3364345.89,3915144.55)	189.04(175.06,202.21)	3738604.77(3455740.61,4029190.31)	187.56(174.46,200.88)	-0.39(-2.91,2.19)
High-middle SDI	3416919.49(3102303.52,3746480.93)	184.13(167.67,202.10)	3572747.46(3232637.57,3922372.55)	184.66(168.23,202.63)	0.14(-0.63,0.92)
Middle SDI	5032306.30(4560445.49,5538716.71)	203.17(185.89,221.34)	5330087.72(4819505.41,5856107.18)	203.26(185.58,221.23)	0.02(-1.16,1.22)
Low-middle SDI	3086585.28(2860185.53,3316828.30)	227.11(211.98,243.23)	3238337.55(2999595.01,3483041.98)	227.24(212.55,242.96)	0.03(-0.21,0.27)
Low SDI	943663.66(874651.13,1015177.88)	186.57(173.55,199.28)	1003161.23(931213.77,1080774.13)	188.01(174.28,201.15)	0.38(-0.07,0.84)
Andean Latin America	75041.32(66133.53,84321.50)	132.09(116.08,148.44)	76153.32(67002.44,85720.13)	129.53(113.95,146.07)	-0.97(-8.08,6.68)
Australasia	63497.06(56861.48,71218.66)	130.73(117.63,146.30)	64995.32(57948.66,73958.30)	125.74(112.75,142.68)	-1.93(-12.58,10.02)
Caribbean	71214.58(64675.30,78454.17)	136.83(124.10,150.61)	73259.33(66093.32,80323.05)	136.21(122.61,149.19)	-0.23(-3.08,2.71)
Central Asia	127469.74(114806.08,140061.44)	166.43(149.42,183.84)	133302.01(118935.70,148130.94)	166.10(148.28,184.13)	-0.10(-1.57,1.40)
Central Europe	353383.11(317132.17,390725.90)	170.63(154.46,186.72)	358921.92(320213.90,398310.86)	170.85(154.02,187.89)	0.06(-0.88,1.02)
Central Latin America	424107.17(385126.13,468074.66)	180.75(164.31,199.40)	444187.15(404528.16,485439.78)	180.32(164.59,196.89)	-0.12(-2.50,2.32)
Central Sub-Saharan Africa	81567.99(73261.99,91269.16)	146.74(131.36,162.99)	88281.05(79298.34,98784.22)	149.24(133.76,166.02)	0.85(0.03,1.68)
East Asia	4319321.69(3908540.93,4756099.66)	215.45(197.92,235.20)	4567271.28(4133511.59,5000602.39)	214.45(197.24,233.32)	-0.23(-1.45,1.00)
Eastern Europe	450726.11(388715.74,510407.43)	138.01(119.69,155.50)	466293.04(401442.45,528517.51)	141.15(122.66,159.15)	1.13(-2.00,4.36)
Eastern Sub-Saharan Africa	212919.84(191415.25,235813.81)	114.43(102.77,126.92)	230277.76(206005.14,256110.16)	116.67(103.99,129.63)	0.97(0.61,1.34)
High-income Asia Pacific	576483.55(501383.06,655885.46)	127.75(112.39,143.40)	586742.66(507296.95,672276.90)	125.19(110.04,141.17)	-1.01(-7.52,5.96)
High-income North America	1406930.56(1331951.69,1473910.43)	235.55(224.53,245.94)	1457540.95(1375581.35,1529937.20)	234.36(222.40,245.02)	-0.25(-1.04,0.54)
North Africa and Middle East	773182.38(691476.92,856161.18)	177.64(158.45,196.99)	827234.92(739140.37,922749.89)	179.46(160.40,199.32)	0.51(-2.45,3.56)
Oceania	16932.03(15822.65,18140.89)	242.46(227.68,257.61)	18086.53(16743.47,19481.10)	243.27(229.23,258.09)	0.17(-0.28,0.62)
South Asia	3599303.05(3344944.61,3840722.71)	258.99(242.73,275.24)	3779866.09(3513161.50,4030885.29)	258.26(242.49,273.76)	-0.14(-0.63,0.35)
Southeast Asia	1000680.74(890945.11,1109261.16)	165.88(148.45,183.63)	1070750.09(953956.17,1190856.16)	167.41(149.52,185.92)	0.46(-2.47,3.48)
Southern Latin America	101537.36(91547.56,112472.81)	121.74(109.83,134.54)	104101.82(93191.08,115163.94)	120.12(107.77,132.55)	-0.67(-5.29,4.18)
Southern Sub-Saharan Africa	88211.08(78387.67,98482.34)	155.98(137.96,174.53)	91911.34(81635.06,102392.19)	157.09(138.99,175.94)	0.35(-0.45,1.17)
Tropical Latin America	460915.95(411266.70,514221.26)	191.52(170.46,213.19)	491865.96(437762.58,549168.56)	193.37(171.91,215.04)	0.48(-4.85,6.11)
Western Europe	1674695.29(1530909.77,1822457.80)	191.15(174.46,207.67)	1696122.21(1559293.18,1848141.84)	188.71(173.96,204.91)	-0.64(-2.59,1.35)
Western Sub-Saharan Africa	248364.25(221691.15,276568.91)	114.67(101.25,128.05)	268280.03(238670.69,297721.79)	116.95(102.97,130.95)	0.99(-0.75,2.76)

Abbreviations

ASIR, age-standardized incidence rate, COPD: chronic obstructive pulmonary disease, EAPCs: estimated annual percentage changes, SDI: Socio-demographic Index.

Table S2 The number of prevalence cases and ASPR of COPD between 2019 and 2021 at the global and regional levels.

	2019		2021		
**Location**	**Number**	**ASPR**	**Number**	**ASPR**	**EAPCs**
Global	204278956.72(186874138.01,223843973.05)	2520.67(2306.51,2756.83)	213387446.18(194868122.06,233975904.60)	2512.86(2293.93,2748.52)	-0.15(-1.54,1.25)
High SDI	50908956.35(47146953.71,54693542.01)	2555.49(2384.34,2733.19)	52256394.66(48386829.86,55979572.19)	2527.66(2364.40,2701.80)	-0.55(-3.35,2.34)
High-middle SDI	43846615.39(39451250.42,48860321.18)	2388.21(2158.68,2644.19)	45671522.59(40973796.19,50846209.89)	2385.34(2149.82,2648.74)	-0.06(-0.93,0.82)
Middle SDI	60509948.13(54202015.63,67479527.58)	2468.66(2215.14,2742.12)	63927638.63(57320694.57,71316409.59)	2463.19(2208.84,2735.96)	-0.11(-1.80,1.61)
Low-middle SDI	36917459.92(33519551.96,40385089.82)	2725.21(2478.78,2974.28)	38692639.02(35061911.35,42291464.22)	2726.76(2478.22,2979.00)	0.03(-0.21,0.27)
Low SDI	11927721.66(10807892.35,13060767.16)	2318.95(2108.33,2545.84)	12666829.95(11450844.58,13814461.88)	2337.52(2119.39,2567.85)	0.40(0.08,0.72)
Andean Latin America	974902.83(865306.34,1089717.23)	1709.02(1515.59,1911.55)	978554.64(865310.36,1103424.56)	1658.68(1461.64,1874.88)	-1.48(-11.63,9.83)
Australasia	837684.09(749353.33,931199.09)	1662.84(1499.67,1848.89)	847867.19(751920.12,969504.89)	1580.64(1411.47,1793.95)	-2.50(-16.58,13.95)
Caribbean	988734.35(903210.96,1089343.59)	1902.66(1738.85,2094.67)	1014978.75(916210.05,1117154.67)	1888.04(1704.73,2076.77)	-0.39(-4.49,3.90)
Central Asia	1710696.84(1550855.21,1895867.70)	2265.14(2034.31,2511.55)	1780821.26(1612497.32,1981648.18)	2258.77(2029.80,2508.18)	-0.14(-0.48,0.20)
Central Europe	5115224.41(4643816.85,5623310.18)	2428.07(2215.33,2660.05)	5181540.82(4678087.56,5712556.34)	2427.25(2209.94,2672.32)	-0.02(-0.90,0.87)
Central Latin America	5244810.73(4747732.89,5797115.40)	2235.65(2019.91,2480.29)	5463613.77(4930424.49,6048461.69)	2221.43(1999.23,2453.11)	-0.32(-2.89,2.32)
Central Sub-Saharan Africa	1067087.60(946433.15,1194579.29)	1893.45(1672.14,2146.25)	1153090.40(1027025.01,1292651.31)	1930.50(1703.40,2184.23)	0.97(-0.06,2.01)
East Asia	49311842.16(44016180.36,55551943.85)	2501.31(2234.04,2796.61)	52092964.77(46321786.60,58800865.30)	2484.43(2224.88,2771.41)	-0.34(-2.30,1.67)
Eastern Europe	6907143.49(6080525.58,7769305.58)	2095.20(1858.65,2348.81)	7080729.28(6224108.96,7978156.30)	2125.62(1896.41,2381.92)	0.72(-1.50,3.00)
Eastern Sub-Saharan Africa	3086049.40(2750587.18,3425421.95)	1625.43(1438.73,1811.48)	3325632.62(2964626.17,3692139.64)	1658.85(1469.54,1852.14)	1.02(0.98,1.06)
High-income Asia Pacific	7117978.63(6177934.70,8092145.29)	1556.54(1378.18,1741.40)	7287035.08(6342141.45,8310359.77)	1527.27(1344.70,1724.48)	-0.94(-4.65,2.90)
High-income North America	20803768.31(19783529.21,21683516.30)	3327.05(3171.51,3463.54)	21494712.24(20380509.31,22538270.18)	3298.88(3132.14,3451.88)	-0.42(-2.37,1.56)
North Africa and Middle East	10728600.66(9711570.76,11903162.35)	2491.33(2242.42,2770.57)	11422458.15(10303522.29,12685054.77)	2512.32(2248.38,2803.61)	0.42(-2.71,3.66)
Oceania	183265.79(168918.68,200177.06)	2482.57(2269.82,2710.23)	195494.88(180142.08,213656.56)	2492.77(2275.04,2748.51)	0.21(-0.43,0.84)
South Asia	41746451.37(37883019.99,45529353.85)	3031.47(2750.18,3307.72)	43766611.60(39580899.80,47755743.37)	3019.10(2729.72,3298.67)	-0.20(-0.85,0.44)
Southeast Asia	12832392.14(11499540.14,14271354.08)	2124.64(1893.06,2369.12)	13656278.72(12169347.18,15269436.20)	2136.77(1904.59,2396.24)	0.29(-3.34,4.05)
Southern Latin America	1254164.41(1115606.54,1412756.52)	1501.31(1338.97,1687.92)	1286931.68(1144984.83,1438038.07)	1482.66(1320.77,1654.03)	-0.62(-4.56,3.47)
Southern Sub-Saharan Africa	1193326.18(1052378.59,1326303.50)	2113.39(1871.98,2367.66)	1239234.11(1090868.86,1375534.28)	2127.98(1882.89,2384.97)	0.34(-1.70,2.44)
Tropical Latin America	6031430.66(5342741.63,6778491.91)	2519.40(2219.14,2830.09)	6440524.58(5703139.46,7215021.58)	2546.83(2252.36,2847.04)	0.54(-7.04,8.75)
Western Europe	23400021.43(21269747.93,25587537.03)	2630.47(2417.50,2876.99)	23652788.12(21532743.84,25858983.64)	2590.99(2375.15,2818.23)	-0.75(-2.81,1.35)
Western Sub-Saharan Africa	3743381.26(3329203.05,4165981.66)	1717.82(1517.82,1920.86)	4025583.55(3582881.21,4476126.95)	1749.91(1540.55,1959.17)	0.93(-0.43,2.31)

Abbreviations

ASPR, age-standardized prevalence rate, COPD: chronic obstructive pulmonary disease, EAPCs: estimated annual percentage changes, SDI: Socio-demographic Index.

Table S3 The number of DALYs cases and ASPR of COPD between 2019 and 2021 at the global and regional levels.

	2019		2021		
**Location**	**Number**	**ASDR**	**Number**	**ASDR**	**EAPCs**
Global	77327991.89(71819023.40,82519473.45)	958.62(889.80,1023.31)	79779695.05(74026373.40,86011405.86)	940.66(871.48,1014.59)	-0.94(-2.99,1.15)
High SDI	9955978.54(9256840.40,10546895.85)	478.86(447.48,506.70)	10197032.65(9399334.69,10811522.80)	471.22(437.45,498.84)	-0.80(-5.93,4.61)
High-middle SDI	12892913.81(11625010.13,14247468.32)	695.04(626.40,766.91)	13439404.01(12068466.43,15035539.57)	691.14(621.83,772.74)	-0.28(-1.21,0.65)
Middle SDI	25356633.05(23017096.86,28030540.97)	1090.51(988.89,1205.71)	26679162.31(23958688.03,29735634.14)	1076.67(963.62,1201.24)	-0.64(-3.14,1.93)
Low-middle SDI	22513818.04(20640642.40,24541834.53)	1775.92(1629.32,1935.79)	22727337.53(20885631.75,24793577.27)	1707.90(1558.88,1865.11)	-1.93(-4.04,0.22)
Low SDI	6566329.88(5895361.80,7272481.57)	1509.16(1357.39,1668.00)	6693611.03(6035071.61,7482882.34)	1457.94(1318.76,1617.05)	-1.71(-2.64,-0.78)
Andean Latin America	156099.59(135141.37,179477.44)	279.25(241.63,321.07)	149984.89(127860.72,176079.60)	261.42(222.30,306.62)	-3.24(-10.84,5.00)
Australasia	206680.90(188377.82,220837.58)	391.70(359.85,418.17)	212256.48(192365.38,226399.90)	376.20(343.09,400.39)	-2.00(-22.24,23.51)
Caribbean	232426.32(210131.34,259539.90)	446.85(404.16,499.76)	237302.18(209887.63,269405.10)	439.53(388.88,499.30)	-0.82(-1.09,-0.56)
Central Asia	376053.87(350240.18,403149.24)	511.84(477.14,547.88)	385341.46(348372.88,424298.17)	498.60(452.01,546.10)	-1.30(-5.09,2.64)
Central Europe	913464.25(864942.53,961490.38)	418.70(397.09,440.80)	915004.97(846947.45,983684.49)	413.12(382.55,444.56)	-0.67(-1.81,0.48)
Central Latin America	1165840.92(1096520.30,1230146.82)	509.70(478.48,537.72)	1233671.54(1115697.88,1349665.05)	512.35(462.65,560.08)	0.26(-1.54,2.09)
Central Sub-Saharan Africa	465993.74(344845.16,621512.46)	973.70(723.09,1353.16)	496005.87(367405.53,659994.87)	975.91(720.50,1344.93)	0.11(-0.07,0.29)
East Asia	23074183.49(19928181.74,26844041.74)	1241.97(1072.95,1442.05)	24391744.16(20763409.85,28628224.46)	1217.69(1043.87,1422.79)	-0.98(-1.78,-0.18)
Eastern Europe	1127031.94(1063229.36,1194014.08)	330.52(311.50,349.95)	1120457.46(1038544.13,1207861.85)	324.42(301.04,350.10)	-0.93(-3.64,1.87)
Eastern Sub-Saharan Africa	1131769.01(937527.09,1299937.01)	716.56(592.66,824.94)	1190523.44(990108.32,1364079.45)	713.24(593.00,816.07)	-0.23(-1.31,0.86)
High-income Asia Pacific	924487.18(815416.71,1028723.41)	184.52(164.55,206.12)	958532.44(841285.98,1065148.93)	182.14(162.64,202.95)	-0.65(-6.13,5.15)
High-income North America	4774829.90(4441822.68,5046039.71)	741.22(691.77,782.00)	4946483.09(4577692.79,5222006.58)	736.05(685.79,776.49)	-0.35(-5.17,4.72)
North Africa and Middle East	2436740.59(2276169.98,2641093.93)	609.26(563.96,660.88)	2534741.31(2307239.25,2795708.69)	599.18(546.07,658.22)	-0.83(-2.66,1.03)
Oceania	150604.81(123075.28,180049.90)	2385.68(1965.40,2821.28)	158392.83(127993.44,193144.75)	2351.49(1931.26,2854.06)	-0.72(-4.24,2.93)
South Asia	27704498.85(25129954.06,30269980.11)	2141.76(1947.67,2350.30)	28009599.09(25404007.40,30942200.96)	2049.22(1862.71,2268.73)	-2.18(-3.84,-0.50)
Southeast Asia	5270652.17(4791179.69,5815931.23)	925.82(842.48,1021.44)	5558590.11(4982450.35,6206668.07)	914.73(822.27,1016.35)	-0.60(-6.82,6.03)
Southern Latin America	405269.73(379673.52,426900.48)	475.75(446.77,500.75)	386636.80(359705.12,409515.84)	436.03(405.72,461.73)	-4.27(-7.53,-0.88)
Southern Sub-Saharan Africa	464350.78(433433.97,497009.82)	869.34(810.66,932.19)	480021.01(444989.93,518790.53)	864.13(800.37,929.92)	-0.30(-2.78,2.25)
Tropical Latin America	1344365.54(1254265.01,1412754.02)	565.44(525.60,595.01)	1402590.87(1301692.60,1479539.63)	554.73(513.83,585.11)	-0.95(-4.59,2.83)
Western Europe	3925961.03(3604971.44,4168336.40)	406.75(376.89,431.31)	3886178.55(3551748.75,4135317.27)	390.81(361.46,415.88)	-1.98(-6.01,2.23)
Western Sub-Saharan Africa	1076687.29(959480.00,1196055.52)	574.41(514.23,635.41)	1125636.51(1009836.73,1263285.93)	567.27(510.78,631.22)	-0.62(-2.43,1.21)

Abbreviations

DALYs, disability-adjusted life years; ASDR, age-standardized DALYs rate, COPD: chronic obstructive pulmonary disease, EAPCs: estimated annual percentage changes, SDI: Socio-demographic Index.

Table S4 The number of incident cases and ASIR of COPD between 2019 and 2021 at the national level.

	2019		2021	
Location	Number	ASIR	Number	ASIR
Afghanistan	21165.95(18744.1-23629.88)	183.57(163.12-205.24)	22594.76(20270.06-25158.52)	187.87(165.88-210.8)
Albania	7281.62(6234.06-8235.4)	173.72(149.82-196.53)	7453.06(6398.56-8429.8)	173.46(150.6-195.9)
Algeria	61716.95(55009.21-68870.98)	182.36(161.64-202.48)	66664.75(58482.31-74485.97)	186.54(164.23-207.98)
American Samoa	69.98(62.54-79.26)	159.47(142.77-178.88)	72.61(64.01-81.83)	160.53(142.19-179.82)
Andorra	269.84(242.21-300.31)	192.51(172.24-214.98)	288.6(257.9-318.98)	191.22(170.56-211.25)
Angola	14837.26(13174.19-16694.61)	125.11(110.83-139.06)	16304.94(14293.29-18338.89)	127.73(113.07-143.6)
Antigua and Barbuda	93.34(79.41-106.14)	94.53(81.66-107.74)	99.7(84.16-115.93)	96(81.19-111.48)
Argentina	69248.99(62487.72-77279.75)	128.87(116.29-144.17)	70795.95(63383.59-79256.1)	127.46(114.4-142.51)
Armenia	7668.28(6797.64-8530.91)	183.38(163.56-204)	7701.67(6787.61-8641.49)	181.25(159.71-201.42)
Australia	52301.67(46692.76-58401.73)	128.44(115.71-142.38)	53697.42(47442.78-61621.3)	123.61(110.77-140.86)
Austria	34329.16(30822.02-37529.01)	203.52(183.7-222.87)	34567.24(31162.39-37980.8)	200.32(180.88-219.15)
Azerbaijan	15479.64(13475.45-17345.48)	165.26(143.16-186.3)	16320.83(14021.08-18585.72)	165.76(143.97-188.06)
Bahamas	361.94(312.69-415.01)	93.73(81.58-107.14)	376.12(329.12-426.43)	93.68(82.29-106.05)
Bahrain	1662.06(1442.01-1894.1)	193.99(172.61-213.43)	1880.77(1622.05-2174.94)	196.15(175.39-218.15)
Bangladesh	307609.19(279016.26-333888.32)	238.11(217.09-256.9)	329819.64(302532.63-358417)	238.94(220.71-258.35)
Barbados	442.94(380.85-509.78)	93.52(81.07-107)	464.82(397.98-531.04)	93.81(81.16-106.47)
Belarus	21002.33(18377.1-23699.7)	138.12(122.04-155.27)	21170.36(18439.43-24094.84)	139.19(122.28-157.15)
Belgium	40451.66(36054.95-45081.15)	184.91(166.79-205.25)	40551.13(36350.85-45320.66)	181.99(164.66-201.42)
Belize	361.25(316.47-408.03)	124.92(108.59-141.88)	387.4(342.41-434.66)	124.11(110-139.84)
Benin	6424.62(5683.4-7210.55)	112.41(99.48-127.13)	6988.99(6171.55-7848.65)	114.45(101.01-128.86)
Bermuda	138.11(119.66-164.04)	110.07(95.88-128.96)	143.94(122.7-168.7)	109.87(94.58-127.2)
Bhutan	1446.16(1338.84-1560.13)	251.17(232.85-269.55)	1512.81(1399.93-1629.41)	250.12(231.4-268.98)
Bolivia (Plurinational State of)	13789.78(12034.06-15812.58)	158.79(139.12-182.33)	14220.68(12315.53-16162.03)	159.99(139.53-180.81)
Bosnia and Herzegovina	11046.41(9759.69-12401.45)	184.48(163.49-205.61)	11062.7(9745.96-12414.93)	184.82(164.34-206.54)
Botswana	2198.81(1964.96-2480.65)	148.23(131.67-167.21)	2371.3(2115.49-2661.09)	150.34(133.42-168.27)
Brazil	452060.08(403454.93-504557.24)	192.26(171.16-214.1)	482404.31(429151.21-538570.22)	194.1(172.57-215.91)
Brunei Darussalam	467.03(414.48-520.53)	162.49(146.25-178.77)	508.66(453.36-564.97)	161.15(144.39-177.41)
Bulgaria	22969.41(20000.94-26215.18)	168.96(148.4-190.51)	23066.39(19640.11-26631.56)	171.35(149.61-195.04)
Burkina Faso	10348.75(9164.48-11724.13)	102.48(90.27-116.32)	11099.8(9819.11-12653.27)	104.66(91.44-119.98)
Burundi	7137.44(6381.98-7970.92)	135.5(121.24-151.18)	7799.93(6923.57-8612.61)	137.37(122.66-151.79)
Cabo Verde	300.1(264-339.31)	65.49(57.6-74.17)	316.49(276.63-357.25)	66.76(58-75.43)
Cambodia	19157.99(17045.46-21320.72)	166.64(147.69-186.09)	20584.8(18068.48-23057.13)	169.07(149.91-190.25)
Cameroon	14309.77(12649.09-16164.05)	102.6(91.18-116.79)	15669.9(13851.47-17681.01)	105.19(91.87-119.63)
Canada	104127.54(91469.94-115119.36)	158.61(141.49-174.83)	107795.54(95822.82-119839.15)	157.07(141.01-173.42)
Central African Republic	3891.04(3492.52-4365.5)	167.55(149.86-184.62)	4126.59(3660.86-4621)	170.67(153.4-189.33)
Chad	8028.85(7166.15-9008.09)	122.34(108.75-138.38)	8685.16(7671.96-9784.31)	125.56(109.93-140.98)
Chile	25430.38(22332.05-29007.18)	105.01(92.34-119.71)	26488.45(23199.38-29550.39)	103.22(90.55-114.84)
China	4191542.01(3788515.9-4622461.54)	216.6(198.8-236.67)	4434437.77(4008615.31-4860417.98)	215.62(198-234.9)
Colombia	98802.59(90186.28-108899.38)	188.92(171.67-208.9)	104676.34(95070.68-113447.92)	189.37(172.45-205.66)
Comoros	515.68(457.94-574.1)	104.92(93.21-117.11)	552.73(488.95-621.3)	106.87(94.67-120.59)
Congo	3502.82(3117.69-3952.63)	130.35(115.72-145.92)	3816.49(3367.36-4328.45)	132.92(117.54-148.98)
Cook Islands	33.02(28.68-37.69)	138.27(121.28-156.74)	34.86(29.34-40.58)	139.18(118.73-159.75)
Costa Rica	8766.23(7903.49-9760.56)	170.94(153.64-191.32)	9434.83(8356.32-10516.76)	172.81(152.83-191.95)
Croatia	15610.85(13459.6-17459.56)	184.8(162.21-204.91)	15846.52(14025.22-17764.94)	185.7(166.44-204.25)
Cuba	28902.56(26113.75-31772.88)	155.06(140.02-170.41)	29061.32(25828.29-32648.16)	152.54(135.31-170.49)
Cyprus	3600.23(3204.26-4013.81)	188.84(168.91-208.43)	3827.04(3381.84-4248.81)	187.37(167.43-207.49)
Czechia	34706.42(30305.3-39190.46)	175.17(155.42-194.51)	35337.59(30951.09-39535.05)	174.64(155.63-192.73)
Cote d'Ivoire	14014.53(12407.06-15874.68)	110.95(97.2-125.49)	15180.39(13407.31-17239.03)	113.45(99.76-129.25)
Democratic People's Republic of Korea	78373.56(72836.79-84795.97)	256(239.13-274.9)	81048.89(75210.56-86977.24)	254.39(237.39-271.66)
Democratic Republic of the Congo	57450.19(51450.38-64553.42)	154.63(138.83-171.59)	62003.01(55414.93-69579.62)	157.23(140.79-175.45)
Denmark	22091.78(19655.83-24460.05)	204.39(183.93-224.25)	22797.68(20383.74-25125.27)	203.89(183.28-222.57)
Djibouti	657.76(577.14-740.33)	97.99(86.45-110.99)	729.25(633.75-827.5)	100.03(86.93-112.73)
Dominica	86.08(73.29-99.13)	108.04(92.26-124.21)	89.19(76.48-102.33)	108.99(94.57-125.13)
Dominican Republic	11569.84(10115.19-13461.29)	119.78(104.23-139.91)	12120.96(10548.93-13993.63)	120.04(104.18-138.84)
Ecuador	21891.82(19030.49-24882.16)	141.75(123.55-161.2)	22935.21(20104.38-26069.14)	141.42(123.96-160.28)
Egypt	105248.74(92698.69-119252.76)	174.05(153.06-195.69)	112961.42(98489.67-128296.89)	177.45(154.51-200.8)
El Salvador	9175.29(8050.36-10482.27)	147.43(128.63-168.5)	9535.36(8358.87-10967.85)	149.18(129.97-171.62)
Equatorial Guinea	702.7(620.63-784.53)	130.61(113.6-146.69)	766.59(683.59-854.89)	132.94(117.24-150.05)
Eritrea	3371.9(2969.21-3795.77)	109.95(98.06-122.3)	3671.37(3236.3-4174.57)	112.06(99.51-126.22)
Estonia	2829.07(2404.19-3201.33)	113.47(99.05-126.98)	2743.39(2382.09-3136.06)	107.42(94.89-120.93)
Eswatini	858.91(765.71-958.46)	145.02(129.35-161.21)	891.47(797.48-995.97)	146.46(131.16-164.16)
Ethiopia	56552.43(50326.86-62722.56)	116.04(103.53-130.16)	60842.18(54291.42-67459.46)	118.6(105.47-132.04)
Fiji	918.55(804.73-1044.09)	132.27(116.83-150.89)	947.18(826.98-1085.07)	132.13(116.15-151.81)
Finland	17390.8(15254.61-19807.13)	151.87(135.08-171.97)	18019.9(15787.21-21036.12)	152.07(134.25-173.93)
France	203398.32(177410.62-229440.83)	154.39(135.51-173.78)	207185.76(185820.91-232556.9)	152.92(137.9-171.76)
Gabon	1183.99(1038.96-1334.03)	115.29(100.47-130.04)	1263.43(1107.65-1453.94)	117.56(102.91-134.01)
Gambia	1288.91(1154.69-1434.33)	117.56(104.29-131.11)	1374.12(1224.01-1533.1)	119.82(105.79-134.96)
Georgia	8333.62(7191.17-9475.96)	143.4(124.74-163.35)	8313.55(7104.14-9499.03)	144.78(124.6-165.12)
Germany	363666.6(327145.86-400893.1)	200.17(180.22-221.49)	368040.88(330493.58-404304.29)	198.21(180.61-217.4)
Ghana	19820.15(17528.49-22278.36)	112.31(99.09-126.58)	21374.36(18907.74-23899.92)	114.47(100.93-129.86)
Greece	43568.24(39108.28-47834.66)	187.1(168.43-205.41)	43663.62(39198.6-48522.46)	185.55(168.03-206.36)
Greenland	138.24(125.7-151.92)	199.24(181.56-218.19)	142.52(130.81-155.51)	197.71(181.31-215.8)
Grenada	118.94(102.53-135.33)	108.34(94.47-123.09)	122.37(106.51-137.35)	108.81(94.64-121.62)
Guam	243.01(209.97-282.04)	121.47(104.7-140.6)	255.09(219.33-292.98)	121.88(104.92-138.52)
Guatemala	15595.7(13881.97-17319.26)	151.01(134.2-167.49)	16673.33(14637.18-18699)	153.01(133.38-171.53)
Guinea	7588.36(6719.34-8480.66)	122.02(107.37-136.93)	8003.51(7134.06-8889.58)	124.83(110.88-139.38)
Guinea-Bissau	1005.87(893.33-1122.3)	117.22(105.58-130.97)	1068.37(941.47-1202.36)	119.06(104.89-133.83)
Guyana	620.55(530.01-712.66)	98.93(85.49-113.79)	641.34(553.45-730.02)	100.3(86.72-114.21)
Haiti	10375.3(9234.86-11683.77)	145.68(129.46-164.22)	10919.67(9790.67-12448.4)	146.43(130.62-166.22)
Honduras	12331.7(11007.47-13804.65)	206.78(185.74-229.65)	13063.74(11702.23-14711.09)	207.62(186.1-232.15)
Hungary	37113.01(33134.47-40664.76)	206.73(186.02-224.73)	37252.1(33117.82-41576.14)	204.62(184.02-224.39)
Iceland	1022.41(917.11-1125.3)	194.03(174.58-213.14)	1075.9(958.68-1188.26)	193.85(173.47-214.02)
India	2955644.84(2752112.73-3145158.45)	263.25(247.04-278.64)	3097721.5(2885223.27-3289929.49)	262.45(246.14-277.48)
Indonesia	376045.37(331713.74-423914.51)	173.72(153.71-194.18)	405551.12(354711.28-457362.76)	176.01(155.46-196.12)
Iran (Islamic Republic of)	110007.6(95748.75-124005.03)	151.98(132.27-172.17)	115756.55(100692.46-131057.53)	152.05(132.29-172.9)
Iraq	31514.35(27569.7-36181.62)	133.62(115.97-156.23)	34172.55(29608.57-39120.92)	137.16(119.06-159.21)
Ireland	13806.87(12319.49-15349.48)	188.05(169.01-208.16)	14718.2(13329.13-16170.95)	189.62(172.35-208)
Israel	19032.1(16752.17-21049.57)	167.44(148.02-184.8)	19975.55(17381-22450.77)	165.8(144.88-184.58)
Italy	243145.44(214753.52-271246.34)	168.5(148.7-188.77)	243979.7(214064.17-272575.48)	166.01(145.95-185.7)
Jamaica	3614.8(3205.84-4075.27)	117.74(104.03-133.75)	3746.55(3346.55-4188.88)	118.73(105.78-133.06)
Japan	417184.64(353004.5-484672.45)	109.93(94.06-127.18)	422531.87(354734.52-494365.59)	108.24(92.19-126.11)
Jordan	10291.91(8947.63-11681.72)	148.44(129.73-169.29)	11822.57(10263.02-13566.24)	150.5(129.68-171.44)
Kazakhstan	36175.66(32635.77-40234.28)	212.91(192.48-235.94)	37344.51(33750.64-41199.91)	212.03(192.05-233.31)
Kenya	26591(23451.74-29661.29)	110.02(97.44-123.2)	28664.35(25173.53-32052.4)	111.83(98.84-125.45)
Kiribati	130.88(117.99-145.29)	193.06(172.08-214.2)	137.27(122.47-152.52)	193.34(172.53-215.66)
Kuwait	4063.74(3546-4666.22)	139.31(120-161.36)	4588.17(3996.22-5231.29)	141.09(121.99-162.59)
Kyrgyzstan	9090.16(8138.28-10084.28)	200.01(178.07-222.61)	9578.32(8461.26-10651.93)	199.5(176.65-221.23)
Lao People's Democratic Republic	8256.74(7403.34-9153.27)	186.81(166.78-207.91)	8882.2(8008.72-9847.61)	189.44(170.32-208.93)
Latvia	4409.45(3716.98-5099.11)	118.94(102.74-135.9)	4466.77(3828.92-5184.28)	120.67(105.33-137.15)
Lebanon	10294.1(8998.43-11625.25)	174.73(152.89-197.63)	10560.45(9170.45-11975.38)	172.93(149.4-196.33)
Lesotho	2053.03(1844.27-2286.53)	181.52(162.57-200.62)	2068.21(1862.2-2312.43)	183.59(165.04-204.04)
Liberia	2790.65(2480.15-3159.62)	122.38(107.54-137.1)	3016.77(2664.56-3396.3)	124.1(108.06-139.31)
Libya	8045.8(6895.13-9198.92)	153.99(131.77-176.56)	8553.09(7504.14-9752.83)	153.68(132.91-175.41)
Lithuania	6827.71(5929.88-7857.51)	126.35(111.72-141.87)	6889.37(6084.26-7850.42)	127.84(113.87-143.72)
Luxembourg	1828.62(1627.05-2020.46)	186.2(166.42-204.83)	1922.76(1716.63-2141.74)	185.26(165.98-205.46)
Madagascar	16570.83(14837.54-18505.91)	139.77(125.8-154.25)	17869.12(15960.1-20146.94)	140.83(125.79-156.11)
Malawi	8537.89(7534.67-9627.07)	103.28(92.23-116.56)	9266.68(8272.26-10375.77)	105.46(94.43-117.92)
Malaysia	35336.14(30833.09-39942.39)	135.33(117.79-153.6)	38204.11(33185.68-43222.26)	137.35(120.05-155.98)
Maldives	562.48(511.61-619.91)	176.47(158.2-195.02)	628.81(561.34-711.08)	178.89(159.64-199.56)
Mali	11713.41(10331.72-13041.68)	113.73(99.55-128.46)	12688.39(11237.4-14125.64)	116.95(102.35-130.97)
Malta	1350.29(1171.45-1562.55)	153.61(135.23-175.26)	1415.31(1211.92-1615.96)	153.13(133.62-172.82)
Marshall Islands	56.31(50.41-62.84)	178.62(159.06-197.35)	59.87(53.48-67.03)	179.46(161.25-198.46)
Mauritania	2182.16(1909.99-2460.44)	97.21(85.62-111.46)	2345.63(2066.34-2689.98)	99.72(86.46-115.01)
Mauritius	2004.3(1741.66-2265.89)	121.89(106.59-137.17)	2145.35(1899.59-2404.03)	123.86(109.97-138.12)
Mexico	218167.19(194233.72-244176.41)	185.51(165.46-206.44)	226608.47(201015.14-253434.22)	183.54(163.7-204.71)
Micronesia (Federated States of)	128.58(115.03-143.06)	187.55(167.77-208.18)	133.89(119.93-150.2)	188.65(169.28-209.41)
Monaco	154.17(134.82-176.46)	170.3(150.49-192.78)	155.57(135.71-179.26)	169.11(147.55-191.49)
Mongolia	3394.92(2945.97-3874.37)	159.85(139.07-182.35)	3624.49(3172.57-4138.45)	159.08(139.78-181.61)
Montenegro	1333.69(1126.38-1546.08)	141.78(121.06-163.59)	1339(1131.65-1528.25)	141.68(120.75-161.12)
Morocco	53448.34(46479.15-60865.49)	163.54(142.1-186)	56785.62(49207.2-64159.49)	166.73(144.54-187.69)
Mozambique	13179.71(11570.32-14715.48)	105.3(93.71-118.76)	14199.66(12480.14-15938.43)	107.81(94.62-120.48)
Myanmar	105898.6(96891.22-114988.27)	234.4(216.11-252.62)	111392.55(102391.31-121439.33)	234.81(216.93-253.66)
Namibia	2154.41(1927.96-2410.19)	153.96(137.14-171.5)	2266.24(2021.11-2538.39)	155.2(138.31-174.41)
Nauru	10.47(9.36-11.62)	187.8(166.46-207.84)	10.82(9.59-12.05)	188.34(167.47-210.63)
Nepal	68314.69(65553.43-70559.69)	311.5(301.16-319.87)	71018.83(68252.51-73545.02)	310.58(300.58-319.7)
Netherlands	56388.42(50477.92-61732.35)	176.91(160.41-192.71)	56014.29(49976.12-62810.24)	168.86(153.16-187.32)
New Zealand	11195.39(9502.76-12969.45)	143.04(122.91-164.03)	11297.9(9599.75-13153.89)	137.06(117.49-158.91)
Nicaragua	8491.77(7653.11-9314.53)	186.47(168.13-203.88)	9000.82(8143.69-9984.65)	187.67(171.01-207.41)
Niger	11530.34(10229.67-12903.78)	128.02(112.33-144.21)	12556.29(11122.11-14135.68)	130.48(115.16-147.62)
Nigeria	118152.66(103594.19-132143.17)	117.32(103.18-132.07)	127617.93(111389.28-142699.33)	119.4(104.78-134.34)
Niue	3.15(2.72-3.63)	152.65(132.36-174.77)	3.21(2.83-3.67)	154.17(136.89-175.28)
North Macedonia	6021.42(5275.09-6773.95)	189.47(169.01-211)	6186.75(5323.47-7050.55)	190.6(168.11-213.96)
Northern Mariana Islands	66.76(57.59-76.37)	147.87(128.64-169.43)	71.19(60.53-82.3)	148.66(129.17-170.77)
Norway	19045.05(17012.45-21099.22)	206.51(184.17-229)	19622.18(17409.91-21786.55)	203.98(181.29-226.56)
Oman	3520.11(3102.33-3987.1)	169.99(148.24-190.18)	4016.72(3532.67-4614.78)	171.74(150.36-196.07)
Pakistan	266288.17(242226.13-294220.9)	232.58(212.9-252.7)	279793.32(254186.73-308153.47)	231.91(212.01-251.5)
Palau	31.07(26.86-35.34)	162.22(141.74-182.52)	33.23(28.95-37.8)	162.97(143.92-184.32)
Palestine	3769.54(3277.25-4270.52)	149.85(128.6-171.05)	4123.03(3656.21-4666.72)	153.14(135.6-173.71)
Panama	6180.43(5587.4-6911.63)	147.52(133.01-165.41)	6721.53(6062.11-7401.36)	150.98(135.88-166.59)
Papua New Guinea	12989.07(12145.8-13867.02)	279.32(263.7-295.19)	13957.79(12953.11-14962.3)	279.71(263.93-294.94)
Paraguay	8855.87(7722.16-10047.46)	160.18(138.09-182.51)	9461.66(8320.11-10726.27)	162.66(142.95-184.27)
Peru	39359.72(34435.12-45119.85)	120.31(104.52-137.53)	38997.43(33788.37-44701.26)	115.43(99.5-132.57)
Philippines	128584.96(113864.37-143611.69)	163.03(143.59-182.15)	137373.4(121537.76-153817.85)	165.78(145.92-185.65)
Poland	103558.81(89569.09-117459.59)	156.18(135.89-176.17)	107057.57(92795.89-121341.93)	157.87(137.69-178.17)
Portugal	40985.49(36275.08-45315.21)	172.81(154.55-191.31)	42039.16(37939.23-46817.33)	172.46(156.78-191.77)
Puerto Rico	9022.96(8017.32-10121.94)	133.26(118.92-148.45)	9349.28(8190.21-10522.15)	133.52(116.97-148.22)
Qatar	2069.42(1770.06-2408.63)	174.25(152.84-195.68)	2269.89(1942.1-2637.57)	176.27(156.2-195.95)
Republic of Korea	152648.98(138257.86-166278.92)	176.31(160.69-191.95)	157017.24(142009.85-172923.91)	170.31(155.14-186.7)
Republic of Moldova	7831.87(6771.14-8917.26)	139.91(121.5-159.07)	7997.79(6832.76-9200.02)	140.73(121.65-161.31)
Romania	58972.97(51719.16-66438.4)	167.93(148.53-188.82)	58890.78(51806.32-66383.19)	166.88(148.83-186.47)
Russian Federation	310132.83(265949.17-351095.99)	140.09(121.42-158.17)	323927.31(277850.43-368283.14)	144.19(125.09-162.45)
Rwanda	8476.4(7517.65-9476.18)	128.12(114.54-143.29)	9223.23(8217.6-10244.34)	130.22(115.71-145.08)
Saint Kitts and Nevis	69.64(60.07-79.96)	109.06(95.36-124.06)	73.03(61.76-84.04)	107.99(93.72-123.31)
Saint Lucia	291.06(258.02-331.19)	128.82(114.32-146.93)	306.23(268.1-343.06)	129.24(113.76-144.91)
Saint Vincent and the Grenadines	126.53(108.33-143.61)	94.57(81.34-106.95)	131.63(114.14-148.85)	94.79(82.73-107.06)
Samoa	255.04(227.23-285.84)	185.2(164.07-207.09)	265.42(236.8-297.18)	186.29(166.16-208.54)
San Marino	109.66(95.2-124.23)	154.18(134.5-174.65)	112.72(97.46-128.54)	153.24(133.24-175.47)
Sao Tome and Principe	184.94(167.1-203.43)	159.47(144.04-175.19)	199.01(180.22-219.38)	160.74(146.19-176.27)
Saudi Arabia	29789.75(26084.5-34456.09)	145.05(126.33-165.14)	33609.84(29092.59-38905.64)	148.56(128.95-169.91)
Senegal	9235.63(8209.73-10461.86)	112.6(98.87-128.65)	9785.28(8649.16-11059.52)	113.91(100.85-129.42)
Serbia	30078.76(26396.41-33416.38)	192.44(169.23-213.13)	30207.19(26560.07-33790.09)	192.03(170.87-212.11)
Seychelles	145.23(125.54-165.3)	135.71(117.85-154.8)	156.33(136.01-177.38)	138.13(120.91-156.83)
Sierra Leone	4772.01(4183.32-5380.65)	114.29(100.27-129.4)	5207.66(4608.07-5817.92)	117.33(103.82-132.04)
Singapore	6182.91(5356.19-7236.96)	80.27(69.24-93.48)	6684.89(5780.66-7729.7)	79.2(68.44-91.95)
Slovakia	13037.28(11174.07-15036.4)	147.6(127.38-169.18)	13390.76(11652.32-15343.58)	147.25(129.25-167.64)
Slovenia	6475.34(5525.68-7430.06)	157.68(135.53-177.33)	6607.28(5823.34-7573.2)	156.74(138.16-177.37)
Solomon Islands	736.9(672.76-804.66)	222.8(202.81-242.1)	783.67(718.49-857.36)	223.4(205.51-244.62)
Somalia	10739.26(9574.84-11994.91)	148.74(132.56-164.27)	11752.18(10348.81-13233.68)	151.7(135.05-169.2)
South Africa	72039.24(63884.9-80754.41)	160.19(141.6-179.19)	74860.98(66359.84-83623.18)	160.94(142.36-180.59)
South Sudan	4698.35(4105.14-5358.73)	111.41(98.76-125.38)	5024.7(4456.25-5653.23)	113.57(101.13-126.1)
Spain	184620.46(167222.31-202818.93)	197.17(178.04-217.38)	186360.46(169640.5-203946.63)	195.06(177.86-215.14)
Sri Lanka	35149.02(30501.7-39944.9)	144.18(126.52-162.9)	38090.31(32969.5-43624.27)	146.56(127.48-167.26)
Sudan	34859.81(30827.39-39586.22)	170.33(149.08-195.16)	37141.59(32776.96-41730.44)	172.64(149.8-195.46)
Suriname	663.46(577.23-760.67)	110.23(96.06-127.04)	701.42(610.47-807.73)	111.51(97.23-128.06)
Sweden	43406.86(38531.02-47685.16)	219.52(196.55-242.95)	44498.04(39754.3-48673.58)	219.08(196.22-242.26)
Switzerland	30444.9(27438.01-33877.54)	181.68(165-200.71)	31132.24(27613.96-34754.54)	179.05(160.06-199.83)
Syrian Arab Republic	20944.67(18354.21-23846.68)	171.58(151.14-193.22)	22378.98(19565.28-25473.29)	173.58(153.48-195.22)
Taiwan (Province of China)	49406.12(43133.84-56030.1)	125.44(109.5-142.13)	51784.62(45643.69-59256.38)	123.69(110.02-140.57)
Tajikistan	10654.58(9569.41-11842.01)	190.12(167.64-212.61)	11442.9(10161.75-12784.23)	190.96(168.77-214.66)
Thailand	125684.67(109495.5-142453.61)	127.25(110.74-143.5)	131403.53(111977.5-151027.01)	125.47(107.46-143.17)
Timor-Leste	1483.03(1322.61-1657.17)	183.55(164.88-203.15)	1588.22(1413.62-1766.01)	185.17(166.53-204.55)
Togo	4669.47(4124.94-5308.21)	111.23(98.69-125.95)	5098.84(4470.54-5794.07)	113.47(99.59-128.38)
Tokelau	2.2(1.93-2.46)	151.79(133.91-170.05)	2.25(1.98-2.54)	152.41(134.09-171.51)
Tonga	124.16(110.09-139.76)	157.52(139.48-177.73)	127.36(111.88-143.8)	158.41(139.25-179.51)
Trinidad and Tobago	1756.14(1509.94-1996.48)	97.69(84.82-111.07)	1859.88(1602.61-2156.22)	99.59(86.65-114.72)
Tunisia	23605.48(20738.61-26551.65)	190.17(167.13-213.17)	24962.39(22040.86-28124.96)	191.45(169.11-214.26)
Turkey	199131.21(179662.95-216828.13)	225.35(203.29-244.57)	210198.95(190484.36-230494.14)	225.79(204.98-245.78)
Turkmenistan	4273.08(3721.37-4934.51)	110.84(94.07-128.78)	4502.61(3903.41-5157.22)	112.22(97.02-127.75)
Tuvalu	16.84(14.94-18.8)	174.43(155.39-193.09)	17.61(15.59-19.73)	175.64(156.53-195.99)
Uganda	19304.81(17191.72-21570.04)	117.49(105.35-131.46)	20910.91(18649.7-23279.03)	119.46(105.81-133.85)
Ukraine	97692.85(83653.24-113467.71)	133.25(114.82-152.19)	99098.05(84314.29-115428.02)	134.44(115.05-154.74)
United Arab Emirates	11840.08(10110.75-13950.67)	186.55(165.26-209.26)	13872.66(11722.52-16428.64)	188.42(167.22-211.98)
United Kingdom	289127.9(265182.71-309711.19)	241.74(221.87-260.09)	292664.93(266676.58-314235.93)	238.42(218.02-256.74)
United Republic of Tanzania	27998.01(24809.57-31190.2)	100.46(89.18-112.46)	30340.26(26891.32-34282.39)	102.74(91.12-115.88)
United States of America	1302642.37(1235219.79-1365157.92)	244.67(233.26-255.33)	1349580.03(1272709.81-1419061.43)	243.58(231.25-255.05)
United States Virgin Islands	181.54(153.6-217.93)	106.6(91.92-125.14)	185.31(155.51-215.85)	107.54(92.63-123.21)
Uruguay	6852.39(6133.53-7618.73)	129.75(117.14-141.72)	6811.67(6091.34-7628.32)	126.45(112.56-140.9)
Uzbekistan	32399.8(28824.07-36219.48)	133(118.27-149.98)	34473.13(30141.33-38904.84)	133.17(115.14-150.87)
Vanuatu	336.44(302.17-376.72)	209.79(190.26-233.07)	361.16(325.09-401.67)	210.33(191.02-232.6)
Venezuela (Bolivarian Republic of)	46596.27(41620.9-52400.74)	164.4(146.77-185.28)	48472.73(43473.77-54795.12)	166.17(148.31-188.37)
Viet Nam	160976.48(143367.44-178417.33)	176.83(157.45-195.54)	173255.91(154370.02-193339.76)	179.64(158.58-199.16)
Yemen	25478.79(22642.32-28738.28)	176.49(154.91-198.36)	27548.62(24254.72-30991.73)	179.17(155.11-203.53)
Zambia	8404.87(7491.57-9368.49)	107.37(95.58-119.73)	9230.73(8140.36-10380.87)	109.82(97.31-123.16)
Zimbabwe	8906.67(7765.84-10084.88)	122.63(106.96-139.77)	9453.15(8266.5-10830.64)	125.56(108.9-144.45)

Abbreviations
ASIR, age-standardized incidence rate, COPD: chronic obstructive pulmonary disease.

Table S5 The number of prevalence cases and ASPR of COPD between 2019 and 2021 at the national level.

	2019		2021	
Location	Number	ASPR	Number	ASPR
Afghanistan	276034.29(250203-304488.12)	2547.17(2262.27-2853.59)	293963.54(265452.59-325421.03)	2590.12(2315.15-2913.83)
Albania	98240.92(84978.57-112292.87)	2388.81(2090.96-2723.97)	100990.98(87982.82-115685.67)	2389.58(2092.08-2724.78)
Algeria	865355.99(772503.76-970821.75)	2597.01(2326.66-2906.09)	928242.61(819663.15-1038356.83)	2643.64(2331.69-2954.44)
American Samoa	867.8(772.35-984.25)	1967.1(1739.41-2228.66)	895.91(795.93-1007.4)	1975.32(1744.82-2226.52)
Andorra	3771.51(3352.87-4227.15)	2675.47(2386.42-2998.37)	4031.03(3596.29-4534.58)	2655.23(2387.68-2982.29)
Angola	197665.81(174796.33-224038.37)	1631.81(1438.56-1853.78)	216940(189977.89-243494.28)	1672.11(1444.04-1899.12)
Antigua and Barbuda	1275.8(1115.36-1452.41)	1312.73(1145.88-1491.03)	1356.46(1163.96-1556.63)	1325.13(1134.42-1515.79)
Argentina	872390.44(772974.48-987291.64)	1616.31(1431.24-1822.29)	892476.05(798704.41-999970.62)	1601.3(1436.49-1793.4)
Armenia	99968.37(88554.25-111658.1)	2399.89(2130.64-2679.55)	100139(89041.89-113173.59)	2370.28(2110.39-2680.12)
Australia	698280.53(626658.82-778917.27)	1646.75(1487.3-1840.94)	706604.79(624380.41-818768.81)	1562.13(1386.02-1789.86)
Austria	508410.17(457082.71-562954.03)	2939.5(2648.81-3231.73)	511218.58(457335.06-558680.5)	2891.94(2593.55-3153.57)
Azerbaijan	206182.59(179517.18-231259.03)	2245.92(1948.9-2539.89)	216263.68(190401.06-245357.58)	2246.65(1967.69-2555.5)
Bahamas	5062.98(4400.84-5770.75)	1333.27(1157.28-1521.36)	5236.36(4613.77-5896.74)	1320.88(1167.46-1488.52)
Bahrain	21522.3(18901.7-24182.77)	2522.48(2239.8-2830.71)	23865.36(21039.3-27392.51)	2547.63(2251.17-2882.69)
Bangladesh	3749658.67(3365831.49-4169157.54)	2944.08(2634.51-3280.83)	4020093.9(3629428.13-4477582.38)	2956.2(2669.12-3284.81)
Barbados	6127.97(5365.83-6999.77)	1302.08(1144.67-1482.31)	6436.92(5548.59-7310.6)	1305.44(1133.26-1483.27)
Belarus	312135.28(278495.4-349953.18)	2036.39(1822.32-2279.97)	312912.34(275571.48-354243.67)	2046.92(1814.29-2305.15)
Belgium	559796.19(501029.2-631744.19)	2515.63(2272.81-2816.29)	560791.94(504009.09-628348.68)	2469.8(2225.42-2749.37)
Belize	5036.98(4417.18-5675.51)	1760.34(1538.78-1995.87)	5399.03(4748.11-6049.33)	1746.82(1525.5-1967.8)
Benin	97219.46(86581.87-108451.55)	1675.24(1472.69-1903.28)	105518.5(93284.74-117104.54)	1705.2(1495.85-1929.75)
Bermuda	1921.88(1663.7-2226.1)	1529.27(1334.55-1753.08)	1999.87(1710.86-2289.64)	1521.79(1309.73-1727.1)
Bhutan	16187.5(14621.78-18098.52)	2784.82(2506.1-3120.8)	16900.7(15257.96-18836.41)	2772.6(2481.15-3092.09)
Bolivia (Plurinational State of)	169303.95(148035.08-193957.75)	1954.54(1717.18-2239.99)	173920.29(152345.67-197808.88)	1967.35(1724.58-2233.81)
Bosnia and Herzegovina	155091.91(137338.44-173225.19)	2583.95(2311.88-2876.16)	154732.46(137494-174491.95)	2584.7(2311.27-2896.99)
Botswana	29381.6(25994.1-33307.07)	1982.68(1749.26-2272.3)	31547.64(27926-35450.47)	2016.93(1774.24-2287.47)
Brazil	5913132.39(5231518.62-6644166.94)	2528.62(2224.36-2841.69)	6314694.44(5587560.53-7077063.89)	2555.99(2259.52-2860.37)
Brunei Darussalam	4771.3(4161.73-5399.23)	1676.82(1455.29-1894.47)	5191.61(4569.51-5809.96)	1662.05(1457.5-1879.28)
Bulgaria	335453.88(294057.77-378947.44)	2431.95(2152.5-2747.08)	334019.46(289512.28-380136.61)	2453.21(2164.24-2774.09)
Burkina Faso	161982.4(144184.62-183640.17)	1579.59(1397.05-1806.44)	172987.34(153466.19-193704.52)	1608.13(1425.7-1828.58)
Burundi	100528.84(89982.25-112428.63)	1849.79(1645.99-2096.32)	109735.86(97673.97-121235.79)	1878.33(1651.01-2110.58)
Cabo Verde	4537.62(3937.91-5140.87)	983.81(853.27-1117.69)	4804.87(4201.15-5403.46)	1001.29(872.55-1138.04)
Cambodia	254754.61(226199.15-286070.14)	2205.51(1944.47-2481.92)	272673.51(243081.35-308825.27)	2230.67(1969.93-2529.52)
Cameroon	220183.93(195758.7-246448.22)	1539.18(1362.6-1746.29)	239814.74(213803.75-269208.97)	1574.02(1383.41-1800.29)
Canada	1464107.56(1306537.55-1634055.83)	2119.08(1906.88-2359.26)	1522991.43(1358067.57-1700360.2)	2098.38(1880.9-2351.54)
Central African Republic	49216.01(43632.85-54917.72)	2103.49(1864.05-2370.48)	52024.05(45816.86-57955.14)	2145.27(1877.74-2435.77)
Chad	120066.2(106621.31-133466.66)	1817.26(1606.68-2068.5)	129102.43(114268.56-144157.95)	1857.59(1625-2113.48)
Chile	296789.38(259240.97-339361.98)	1231.77(1075.21-1407.57)	310100.26(269434.15-353782.85)	1213.7(1060.34-1388.75)
China	47863720.01(42654843.09-53892395.07)	2516.07(2246.43-2815.83)	50588429.3(44975892.81-57116835.17)	2499.35(2236.21-2793.29)
Colombia	1227786.08(1101397.95-1377969.75)	2328.86(2087.29-2615.93)	1306282.89(1176060.55-1451413.03)	2340.91(2106.1-2604.23)
Comoros	7281.73(6372.3-8241.76)	1462.62(1280.94-1653.85)	7791.46(6800.09-8782.79)	1492.5(1299.98-1696.45)
Congo	45487.7(40162.13-51032.8)	1678.24(1484.38-1893.19)	49376.33(43371.41-55552.48)	1716.96(1501.89-1954.86)
Cook Islands	424.31(363.97-485.67)	1809.95(1563.74-2068.9)	447.73(378.99-512.68)	1821.43(1557.93-2077.04)
Costa Rica	107415.38(95152.42-121245.99)	2073.2(1830.27-2344.36)	115582.72(100152.8-129868.6)	2098.78(1814.84-2368.32)
Croatia	219418.35(194504.88-243389.73)	2540.94(2282.25-2814.82)	222941.35(199359.8-246696.37)	2553.27(2289.68-2833.69)
Cuba	408011.06(370645.64-448396.24)	2182.36(1987.41-2394.46)	409597.99(365089.04-455411.12)	2139.36(1919.69-2374.51)
Cyprus	47151.09(42026.11-52643.87)	2520.33(2260.59-2801.76)	50059.59(44353.03-56084.21)	2496.35(2221.78-2781.09)
Czechia	506272.17(453542.64-561781.82)	2503.16(2242.81-2758.57)	516786.48(462606.72-566688.1)	2498.69(2264.56-2734.86)
Cote d'Ivoire	214092.48(191472.15-238054.49)	1659.77(1463.77-1884.07)	230232.07(204519.02-259208.93)	1691.67(1489.89-1934.8)
Democratic People's Republic of Korea	849876(760087.23-949426.4)	2782.36(2500.32-3106.96)	879586.61(791149.15-981320.44)	2757.5(2487.07-3066.78)
Democratic Republic of the Congo	749874.46(658516.66-843309.81)	1992.46(1752.41-2267.33)	807959.01(717254.36-913626.8)	2029.81(1800.47-2308.63)
Denmark	300867.52(269307.25-336368.69)	2743.5(2484.59-3044.55)	310720.04(275454.26-344044.28)	2732.96(2448.7-3009.8)
Djibouti	9499.67(8187.78-10699.22)	1376.33(1195.75-1556.81)	10503.92(9075.49-11843.85)	1412.18(1230.01-1602.95)
Dominica	1134.5(970.02-1295.98)	1439.55(1232.36-1640.07)	1176.68(1004.95-1352.34)	1451.22(1241.72-1668.9)
Dominican Republic	162133.08(142298.94-185727.12)	1678.41(1471.1-1926.77)	169699.04(147743.97-193588.21)	1680.84(1452.7-1916.87)
Ecuador	268271.3(232023.6-305864.36)	1736.36(1499.26-1985.14)	281618.66(246354.96-320213.21)	1737.48(1521.87-1977.78)
Egypt	1458107.47(1294063.1-1641165.33)	2434.2(2154.91-2736.51)	1555374.12(1364099.22-1775225.21)	2472.66(2161.71-2806.85)
El Salvador	116408.77(102658.17-132418)	1840.2(1617.91-2087.61)	120349.21(104289.62-137641.89)	1855.16(1599.69-2125.36)
Equatorial Guinea	9421.07(8233.55-10544)	1694.58(1474.95-1917.8)	10267.14(8986.2-11530.81)	1732.08(1522.72-1979.46)
Eritrea	47649.27(41842.01-53607.2)	1510.97(1323.69-1722.41)	51716.32(45557.79-58726.04)	1543.62(1344.22-1764.93)
Estonia	41617.68(36738.24-46463.16)	1630.21(1451.11-1834.1)	39751.07(35068.21-44930.31)	1532.4(1367.82-1728.88)
Eswatini	11330.84(10049.65-12647.92)	1894.83(1662.25-2142.59)	11741.73(10401.91-13112.68)	1921.59(1707.84-2173.55)
Ethiopia	832613.62(735263.78-925084.7)	1682.21(1469.04-1880.21)	890979.26(793351.82-987246.84)	1715.27(1509.18-1918.44)
Fiji	11658.15(10292.29-13201.82)	1657.08(1462.2-1876.36)	12013.69(10576.51-13794.98)	1655.84(1446.42-1890.83)
Finland	246976.03(218662.5-278403.73)	2128.54(1894.6-2384.16)	256967.66(224690.91-292252.34)	2133.03(1886.93-2393.42)
France	2919807.01(2574554.17-3321647.75)	2208.57(1980.36-2474.69)	2969242.85(2646069.68-3337983.2)	2182.53(1950.2-2438.77)
Gabon	15422.56(13541.81-17556.88)	1489.75(1292.57-1697.03)	16523.85(14379.92-18762.24)	1530.9(1330.61-1767.76)
Gambia	19400.21(17311.91-21619.37)	1746.53(1546.38-1974.68)	20616.13(18392.7-22929.13)	1778.31(1574.41-2005.21)
Georgia	122747.74(107210.49-138501.27)	2084.92(1842.71-2340.27)	121326.48(105831.53-137326.08)	2091.88(1844.96-2352.67)
Germany	5150554.48(4628959.05-5770618.5)	2789.77(2523.58-3088.06)	5218529.28(4717214.53-5793017.89)	2759.62(2508.82-3043.39)
Ghana	297943.76(261709.75-333646.11)	1639.86(1438.25-1850.37)	320571.81(285498.19-355226.69)	1672.26(1471.4-1876.37)
Greece	606221.23(537641.39-673660.47)	2598.12(2329.57-2878.34)	609189.7(546308.29-683100.14)	2575.73(2325.26-2864.41)
Greenland	1613.54(1443.16-1812.62)	2566.19(2311.72-2869.89)	1677.21(1516.25-1867.77)	2542.79(2306.06-2822.86)
Grenada	1589.8(1388.78-1805.63)	1482.85(1301.81-1686.15)	1630.18(1424.57-1831.3)	1482.2(1299.19-1658.69)
Guam	3236.01(2803.95-3742.33)	1620.33(1405.11-1860.74)	3391.82(2875.42-3841.49)	1623.56(1387.66-1836.26)
Guatemala	196355.06(174978.47-221268.42)	1882.74(1669.42-2123.58)	209544.52(186412.16-236785.77)	1906.12(1694.12-2164.38)
Guinea	113712.27(101140.82-127113.45)	1803.56(1586.72-2036.44)	119666.59(106166.63-132601.46)	1842.2(1625.56-2067.62)
Guinea-Bissau	15186.91(13623.28-16812.42)	1732.33(1534.37-1954.99)	16064.59(14307.97-17918.27)	1757.8(1541.23-2001.48)
Guyana	8646.63(7438.42-9967.42)	1397.96(1194.45-1606.5)	8911.75(7696.87-10104.92)	1411.33(1215.33-1605.97)
Haiti	135512.81(118531.8-151655.33)	1903.6(1661.88-2157.01)	142144.53(125213.22-159936.36)	1909.4(1687.77-2174.25)
Honduras	149030.58(131755.03-168711.89)	2510.97(2213.6-2851.27)	157513.4(139565.15-179516.68)	2521.81(2240.28-2870.89)
Hungary	547843.65(489541.33-601460.5)	2968.32(2678.82-3232.04)	548104.39(491546.81-598032.18)	2933.74(2673.56-3185.9)
Iceland	14963.7(13507.33-16476.54)	2781.42(2520.71-3056.89)	15732(14101.88-17342.04)	2769.55(2492.45-3053.54)
India	34243097.67(30972817.93-37309811.45)	3082.87(2806.36-3363.27)	35819854.91(32396904.15-39076078.78)	3067.86(2782.53-3347.91)
Indonesia	4748055.66(4167603.96-5319633.07)	2180.32(1905.27-2443.9)	5080725.22(4434330.15-5713205.36)	2204.76(1930.54-2481.68)
Iran (Islamic Republic of)	1510138.52(1328745.48-1691382.79)	2050.68(1791.09-2310.33)	1586139.27(1396410.6-1781229.38)	2054.63(1798.37-2321.91)
Iraq	459898.98(405128.93-518472.61)	1961.81(1727.51-2236.5)	494469.97(435909.92-559676.31)	2003.35(1756.16-2283.54)
Ireland	179695.29(158103.86-201153.48)	2450.79(2167.28-2741.2)	191319.43(172627.27-213047.63)	2466.78(2233.2-2735.65)
Israel	270624.59(237035.85-299257.24)	2361.54(2067.06-2601.55)	283970.33(250022.58-317251.13)	2340.1(2084.01-2602.78)
Italy	3161097.45(2749746.2-3586092.5)	2188.85(1921.53-2472.69)	3174977.89(2745407.6-3585532.42)	2159.42(1891.01-2433.84)
Jamaica	50841.6(45156.39-56986.89)	1643.71(1458.27-1848.04)	52551.82(47134.44-58583.53)	1653.88(1478.44-1848.05)
Japan	4992033.45(4217012.66-5860620.18)	1300.1(1111.96-1506.23)	5114116.64(4309205.53-6010382.11)	1285.53(1092.96-1492.23)
Jordan	145110.14(129010.88-163858.27)	2130.01(1876.57-2416.64)	165854.29(145994.23-186678.37)	2153.03(1877.41-2442.68)
Kazakhstan	461484.86(416745.42-512110.53)	2783.39(2497.9-3112.66)	474247.13(429214.18-532108.57)	2778.16(2508.07-3109.01)
Kenya	375941.82(329607.37-421415.24)	1502.71(1309.67-1685.64)	403346.96(352451.97-452161.1)	1528.53(1330.32-1716.06)
Kiribati	1593.98(1428.81-1772.2)	2278.15(2014.13-2542.54)	1668.5(1493.97-1876.6)	2277.89(1999.04-2574.47)
Kuwait	59199.71(52044.96-66988.76)	1985.45(1725.73-2281.45)	66467.33(58682.88-74860.72)	2006.85(1773.08-2282.36)
Kyrgyzstan	119377.33(107527.49-132639.8)	2657.75(2374.8-2965.35)	125202.34(111803.3-140538.4)	2653.88(2332.34-2976.49)
Lao People's Democratic Republic	106156.55(95170.72-118235.01)	2366.32(2090.82-2649.02)	113895.91(102552.49-127023.55)	2401.41(2141.97-2705.24)
Latvia	67186.47(58606.85-76598.38)	1782.31(1574.29-2006.21)	67917.24(59522.12-76700.02)	1801.32(1589.74-2021.76)
Lebanon	150894.33(132775.58-171097.27)	2538.57(2235.17-2878.53)	153048.2(132931.66-174115.17)	2481.23(2159.62-2823.22)
Lesotho	26206.66(23335.62-29373.54)	2299.69(2035.36-2593.07)	26389.76(23470.8-29591.7)	2334.74(2076.67-2646.7)
Liberia	40549.21(35847.61-45374.33)	1690.76(1487.6-1932.07)	43723.86(38564.58-49060.76)	1718.18(1498.14-1946.85)
Libya	114761.1(100798.7-128781.98)	2233.52(1942.45-2541.72)	121288.92(107865.79-136197.33)	2224.56(1971.25-2518.3)
Lithuania	101618.91(89613.47-113836.89)	1852.47(1646.98-2068.55)	103040.88(91249.89-116814.19)	1875.04(1673.21-2112.08)
Luxembourg	25543.24(22769.21-28314.89)	2588.38(2317.93-2860.5)	26887.99(24038.89-29849.95)	2570.61(2309.6-2854.1)
Madagascar	230037.36(206550.38-254972.34)	1834.63(1627.03-2072.61)	248160.41(221119.58-277578.19)	1857.98(1648.41-2120.94)
Malawi	126455.87(111092.24-142632.91)	1496.66(1312.04-1708.4)	136848.27(120542.72-154114.19)	1530.87(1352.11-1728.65)
Malaysia	463074.16(406574.12-521276.22)	1759.93(1537.33-1990.03)	499394.92(433574.26-562409.68)	1784.36(1540.75-2022.61)
Maldives	7384.56(6664.85-8174.78)	2233.88(2006.73-2502.14)	8257.03(7362.13-9221.5)	2268.24(1999.51-2573.46)
Mali	181149.74(161954.99-201687.52)	1806.37(1578.53-2040.85)	194654.71(172830.39-214824.16)	1845.71(1626.52-2078.3)
Malta	18804.94(16521.27-21415.87)	2142.26(1904.71-2431.28)	19680.65(17070.5-22217.94)	2133.04(1879.62-2381.1)
Marshall Islands	694.63(626.54-768.78)	2013.5(1753.96-2272.05)	733.33(661.35-814.42)	2024.13(1797.43-2296.13)
Mauritania	33447.31(29386.3-37961.68)	1464.31(1288.62-1671.15)	35872.07(31656.02-40877.54)	1500.83(1316.64-1725.33)
Mauritius	27531.55(23732.75-31236.75)	1668.03(1449.28-1887.33)	29226.67(25865.58-33113.12)	1687.13(1499.78-1901.07)
Mexico	2672932.5(2351652.84-3018493.54)	2283.51(2011.24-2572.48)	2747439.16(2421692.74-3093515.19)	2238.95(1970.83-2525.77)
Micronesia (Federated States of)	1563.09(1400.56-1760.64)	2204.9(1935.86-2498.64)	1616.67(1462.14-1822.76)	2210.98(1956.06-2493.15)
Monaco	2196.08(1920.05-2481.3)	2399.64(2127.67-2687.17)	2220.05(1929.09-2511.77)	2386.91(2120.48-2666.93)
Mongolia	45789.45(40201.12-51627.93)	2198.82(1908.42-2511.3)	48865.48(43338.29-54857.35)	2187.97(1928.74-2468.99)
Montenegro	18937.95(16197.2-21663.32)	2059.79(1777.93-2342.26)	18890.01(16209.13-21406.07)	2053.55(1782.44-2305.68)
Morocco	751766.88(655718.87-859813.38)	2321.28(2032.93-2653.67)	796614.09(701585.19-903040.63)	2361.44(2081.75-2665.44)
Mozambique	195658.59(172002.88-218134.39)	1534.94(1347.15-1730.9)	210038.26(184700.06-234148.98)	1571.19(1375.56-1771.66)
Myanmar	1231951.07(1111128.26-1371186.87)	2708.63(2438.96-2997.58)	1295568.44(1176296.9-1449782.55)	2719.81(2465.81-3044.72)
Namibia	28214.1(25109.91-31607.24)	2025.8(1785.15-2288.04)	29661.18(26106.51-33277)	2047.18(1801.24-2327.96)
Nauru	126.45(113.5-140.16)	2128.22(1886.95-2397.54)	130.48(117.04-145.81)	2138.45(1864.22-2421)
Nepal	683962.74(636462.47-730641.33)	3067.75(2866.16-3275.41)	710534.53(659222.25-765341.11)	3051.13(2835.24-3271.74)
Netherlands	787200.16(708514.91-864335.56)	2423.81(2224.83-2657.78)	759256.66(682277.2-845740.34)	2263.34(2048.89-2513.4)
New Zealand	139403.56(118440.06-161646.01)	1750.05(1496.61-2020.58)	141262.39(118993.61-166031.43)	1678.32(1429.32-1958.56)
Nicaragua	100750.68(89142.92-112406.6)	2206.79(1947.81-2465.66)	106608.73(93933.97-118461.8)	2223.48(1965.05-2482.65)
Niger	173127.09(154028.69-192091.45)	1911.65(1694.87-2158.75)	187856.39(166784.92-210573.07)	1945.97(1710.19-2209.98)
Nigeria	1765112.26(1556232.22-1962638.94)	1757.68(1534.85-1978.31)	1897949.28(1672847.11-2120293.1)	1788.63(1562.86-2005.8)
Niue	38.73(33.28-44.57)	1904.7(1645.34-2181.69)	39.45(34.34-45.34)	1920.92(1678.86-2190.63)
North Macedonia	79506.63(70403.3-90436.69)	2611.91(2332.11-2934.83)	81263.02(70963.47-94240.92)	2624.09(2302.9-3004.59)
Northern Mariana Islands	835.38(729.76-953.74)	1859.05(1615.69-2138.49)	884.67(766.56-1006.27)	1867(1609.47-2151.45)
Norway	263823.62(233990.59-296854.02)	2803.25(2493.06-3145.43)	271735.88(240590.4-307387.89)	2768.83(2464.33-3111.06)
Oman	47615.94(41907.01-53259.64)	2296.71(1997.15-2595.4)	53844.05(47442.87-61113)	2314.58(2036.61-2644.12)
Pakistan	3053544.79(2717597.24-3408917.87)	2619.46(2325.33-2938.37)	3199227.56(2839704.75-3571679.34)	2606.29(2311.9-2925.99)
Palau	388.05(343.16-440.84)	1984.02(1737.04-2233.19)	411.63(361.76-464.09)	1988.29(1735.17-2245.21)
Palestine	52643.3(46249.24-59206.28)	2107.47(1842.9-2395.05)	57385.19(51328.76-64186.67)	2147.67(1912.25-2436.2)
Panama	76855.34(68751.38-85807.51)	1823.04(1627.07-2035.65)	83479.59(75166.92-92863.52)	1863.21(1677.28-2075.51)
Papua New Guinea	136042.25(125435.23-148294.67)	2705.8(2481.61-2947.44)	146155.33(134848.96-158586.85)	2711.59(2480.62-2980.45)
Paraguay	118298.27(103698.23-133756.03)	2125.24(1853.11-2405.53)	125830.14(110754.62-142204.95)	2153.44(1889.7-2446.94)
Peru	537327.58(469079.04-609328.09)	1631.25(1418.35-1857.18)	523015.69(451412.92-597482.97)	1538.03(1327.25-1755.86)
Philippines	1738026.72(1540169.48-1933145.41)	2198.98(1950.36-2460.32)	1835887.89(1631451.22-2046786.63)	2213.41(1960.93-2482.71)
Poland	1517097.97(1318762.39-1715639.21)	2246.24(1981.57-2527.52)	1561336.31(1357982.65-1758296.56)	2262.26(1997.88-2539.22)
Portugal	543606.56(478746.65-610552.89)	2326.51(2080.01-2600.6)	557534.33(501098.26-625041.88)	2318.03(2088.23-2583.42)
Puerto Rico	124168.12(109969.6-139096.7)	1804.04(1605.63-2004.3)	128481.4(111721.45-143979.47)	1802.96(1581.24-2026.47)
Qatar	29397.85(25615.6-33050.01)	2357.65(2077.98-2664.52)	32092.35(28315.43-36059)	2384.48(2104.19-2686.75)
Republic of Korea	2049836.6(1865870.32-2249329.44)	2388.87(2180.73-2616.69)	2090259.56(1904590.58-2305737.55)	2287.85(2091.34-2510.81)
Republic of Moldova	115436.47(100755.14-131824.13)	2048.88(1805.22-2332.67)	117569.83(102730.12-134548.38)	2054.31(1805.95-2331.16)
Romania	853379.87(756974.06-955235.39)	2385.06(2128.72-2648.04)	849850.37(757217.45-951582.57)	2367.71(2125.87-2635.41)
Russian Federation	4826018.78(4235590.83-5441866.3)	2154.42(1906.47-2421.69)	4982424.29(4358539.7-5631745.75)	2196.02(1945.53-2463.55)
Rwanda	121569.83(107903.57-135496.44)	1797.63(1592.54-2030.7)	131916.63(116357.76-146789.63)	1830.31(1621.65-2058.32)
Saint Kitts and Nevis	914.58(801.58-1037.93)	1477.49(1293.06-1678.12)	960.61(824.14-1111.01)	1457.74(1255.85-1677.64)
Saint Lucia	3925.96(3465.62-4430.32)	1753.48(1552.55-1971.17)	4138.28(3601.03-4647.2)	1761.21(1536.13-1974.36)
Saint Vincent and the Grenadines	1746.76(1532-2007.55)	1317.02(1156.09-1506.8)	1811.44(1582.84-2061)	1314.21(1156.11-1497.43)
Samoa	3174.87(2828.95-3570.64)	2281.38(2003.01-2561.46)	3302.83(2952.38-3714.28)	2293.9(2022.62-2584.23)
San Marino	1562.56(1368.32-1774.25)	2168.36(1895.13-2482.9)	1604.16(1389.86-1828.37)	2154.35(1897.78-2430.94)
Sao Tome and Principe	2459.28(2228.74-2718.06)	2059.23(1831.98-2316.92)	2647.29(2401.77-2927.19)	2084.48(1858.31-2339.77)
Saudi Arabia	400884.67(354873.86-448672.92)	2002.58(1763.91-2288.14)	448933.39(391042.3-508906.17)	2050.86(1796.44-2325.25)
Senegal	138794.88(122937.86-155818.95)	1661.03(1459.39-1900.7)	146764.79(129857.53-165174.18)	1680.25(1479.89-1901.31)
Serbia	426871.25(375503.12-475030.23)	2695.63(2376.95-2981.77)	429424.64(384715.39-479782.11)	2692.46(2420.28-2989.34)
Seychelles	1936.67(1695.72-2207.54)	1811.55(1587.2-2060.38)	2072.12(1811.66-2356.39)	1835.81(1598.8-2078.76)
Sierra Leone	73061.58(64839.1-81320.84)	1730.6(1516.41-1973.52)	79279.2(70634.84-88256.43)	1770.38(1550.59-1987.35)
Singapore	71337.29(61980.21-83484.76)	933.68(810.51-1091.11)	77467.27(67346.39-89381.77)	922.56(805.95-1060.47)
Slovakia	188999.31(163813.27-215174.64)	2132.09(1862.03-2417.09)	192936.57(169685.02-218388.72)	2118.52(1878.43-2394.73)
Slovenia	93171.67(80333.72-105411.71)	2194.81(1904.23-2480.92)	94845.66(83121.86-106777.14)	2177.73(1923.82-2436.97)
Solomon Islands	8515.38(7773.64-9405.83)	2485.67(2213.36-2771.99)	9027.23(8290-9978.01)	2490.64(2242.06-2783.82)
Somalia	151002.05(134588.97-166985.48)	2070.91(1836.45-2333.72)	163129.7(146092.73-181015.91)	2116.82(1858.64-2404.17)
South Africa	969925.23(854779.88-1078224.9)	2161.59(1910.54-2416.18)	1004761.53(886683.61-1119026.86)	2170.53(1917.47-2437.23)
South Sudan	67129.07(58398.01-75388.1)	1566.48(1361.13-1786.05)	71608.43(63234.73-80531.09)	1601(1404.86-1818.43)
Spain	2546291.65(2270741.84-2834371.5)	2690.02(2413.25-2973.84)	2570070.73(2308173.71-2861069.55)	2658.24(2409.07-2950.54)
Sri Lanka	457969.68(403247.51-521845.46)	1891.13(1663.99-2151.32)	495569.1(428210.91-568689.16)	1923.09(1670.3-2193.97)
Sudan	487323.28(432485.48-547183.11)	2384.45(2079.32-2713.81)	518223.11(462333.66-578783.86)	2416.13(2114.44-2730.3)
Suriname	9305.51(8136.85-10547.34)	1570.31(1372.62-1774.28)	9822.21(8646.97-11101.15)	1581.99(1391.61-1787.28)
Sweden	639557.24(563965.86-716549.64)	3104.28(2768.73-3469.9)	656865.55(580693.46-731419.81)	3097.64(2760.32-3448.67)
Switzerland	443687.76(402938.88-490288.83)	2591.72(2369.29-2847.87)	451983.3(408103.36-501311.76)	2548.98(2306.28-2820.47)
Syrian Arab Republic	289084.93(256969.24-327520.07)	2419.16(2146.49-2733.19)	307654.03(271843.5-349197.82)	2446.57(2166.79-2749.06)
Taiwan (Province of China)	598246.15(523638.45-678147.98)	1534.03(1343.35-1740.75)	624948.86(546926.12-716439.67)	1512.21(1331.26-1725.43)
Tajikistan	139801.46(125693.64-155806.44)	2508.99(2214.42-2825.84)	149563.12(134319.29-167205.84)	2519.36(2214.07-2838.91)
Thailand	1714844.51(1489483.73-1932539.35)	1746.17(1525.55-1960.97)	1781016.61(1544547.25-2028248.09)	1711(1490.46-1934.21)
Timor-Leste	18444.71(16460.24-20821.52)	2273.1(2011.12-2567.68)	19723.11(17602.39-22257.87)	2295.43(2041.89-2596.01)
Togo	71308.32(63605.12-80083.05)	1695.16(1495.7-1925.7)	77409.93(67732.92-86985.9)	1725.8(1505.49-1960.26)
Tokelau	26.96(23.35-30.79)	1880.31(1637.55-2137.05)	27.66(24.06-31.46)	1887.77(1641.64-2144.29)
Tonga	1538.93(1368.95-1740.45)	1939.39(1715.84-2190.52)	1578(1379.99-1791.24)	1951.31(1690.26-2217.16)
Trinidad and Tobago	25307.52(22051.6-28693.75)	1413.48(1238.9-1592.77)	26719(23035.9-30689.73)	1434.75(1240.91-1640.26)
Tunisia	343594.88(307154.43-387353.43)	2793.79(2494.5-3141.07)	362516.48(323629.14-405804.54)	2808.2(2494.08-3138.15)
Turkey	2743429.81(2483773.92-3012000.64)	3142.81(2850.02-3453.22)	2885480.52(2627311.65-3185404.7)	3146.68(2861.31-3477.82)
Turkmenistan	63839.08(56502.42-72641.53)	1664.96(1461.72-1910.86)	66967.53(59373.45-75767.23)	1680.97(1478.82-1909.51)
Tuvalu	204.34(182.57-230.02)	2078.96(1838.72-2331.15)	213.45(189.13-242.69)	2094.63(1836.1-2383.5)
Uganda	275220.12(241757.72-306244.53)	1630.78(1428.77-1840.62)	297985.24(265625.65-332702.05)	1663.08(1467.43-1879.94)
Ukraine	1443129.89(1249057.19-1648601.24)	1969.86(1717.05-2233.54)	1457113.63(1254134.46-1665203.79)	1978.64(1720.66-2252.75)
United Arab Emirates	157333.52(143103.04-173349.04)	2659.02(2361.12-2986.92)	180019.79(161070.89-203394.62)	2686.42(2401.97-3008.25)
United Kingdom	4137410.92(3722360.13-4528789.23)	3341.32(3031.82-3637.21)	4157373.18(3745187.24-4554835.6)	3270.26(2957.04-3570.38)
United Republic of Tanzania	421686.86(373611.19-471026.8)	1498.9(1314.69-1698.12)	456371.55(401902.84-513570.63)	1538.19(1356.18-1744.38)
United States of America	19337715.72(18382746.31-20190736.41)	3473.21(3303.64-3622.49)	19969706.53(18951147.4-20892543.95)	3445.29(3263.49-3602.42)
United States Virgin Islands	2505.22(2166.82-2941.35)	1469.96(1281.14-1689.3)	2557.19(2201.79-2931.81)	1478.42(1288.27-1679.19)
Uruguay	84915.36(75515.69-94326.67)	1593.42(1431.9-1769.84)	84284.35(73376.3-95642.79)	1553.79(1365.19-1751.77)
Uzbekistan	451505.96(403024.62-504651.53)	1885.29(1679.28-2106.98)	478246.5(425669.45-536721.47)	1880.94(1663.83-2124.79)
Vanuatu	3898.39(3495.56-4339.01)	2312.93(2037.47-2618.97)	4179.33(3771.12-4619.88)	2322.9(2051.12-2603.16)
Venezuela (Bolivarian Republic of)	597276.34(536741.04-674482.76)	2102.05(1884.56-2373.86)	616813.53(554663.96-695928.12)	2120.81(1898.04-2390.93)
Viet Nam	2044363.37(1820207.11-2285256.41)	2249.39(1994.92-2515.98)	2203220.56(1961863.92-2483948.25)	2291.31(2035.09-2592.2)
Yemen	354595.55(316958.49-400408.05)	2448.38(2167.03-2800.6)	380327.76(339852.74-423976.21)	2478.52(2169.71-2808.67)
Zambia	121114.94(107513.76-135220.73)	1504.54(1326.93-1705.55)	132605.08(116576.45-148088.04)	1547.52(1346.26-1774.17)
Zimbabwe	128267.75(112394.24-144073.55)	1758.25(1532.39-1996.61)	135132.28(118220.28-153081.68)	1798.65(1573.57-2076.37)

Abbreviations

ASPR, age-standardized prevalence rate, COPD: chronic obstructive pulmonary disease.

Table S6 The number of mortality cases and ASMR of COPD between 2019 and 2021 at the national level.

	2019		2021	
Location	Number	ASMR	Number	ASMR
Afghanistan	3843.63(2767.97-4977.96)	51.5(36.87-66.03)	3804.55(2729.01-4947.8)	50.6(36.13-65.6)
Albania	642.04(501.49-858.92)	15.94(12.49-21.17)	650.27(503.04-880.3)	15.57(11.92-21.04)
Algeria	5140.84(4136.32-6345.43)	22.63(18.18-27.64)	5316.02(4148.06-6533.78)	22.08(17.4-27.31)
American Samoa	17.32(14.98-19.98)	48.97(42.12-56.19)	17.95(15.33-21)	48.75(41.55-57.11)
Andorra	32.95(24.87-41.27)	20.96(16.03-26.38)	30.12(21.57-39.02)	17.72(12.5-23.07)
Angola	2173.06(1675.8-2789.45)	29.19(22.52-37.91)	2333.41(1765.73-3063.76)	29.26(22.6-38.2)
Antigua and Barbuda	6.88(6.2-7.63)	8.06(7.28-8.9)	7.16(6.65-7.82)	7.71(7.11-8.53)
Argentina	15619.73(14393.13-16616.21)	27.59(25.47-29.33)	14312.38(13094.54-15431.02)	24.51(22.43-26.4)
Armenia	656.74(586.19-710.74)	15.49(13.82-16.77)	658.74(568.12-752.34)	15.19(13.08-17.33)
Australia	9160.8(7959.05-9949.77)	19.03(16.67-20.6)	9482.63(8304.2-10312.09)	18.33(16.16-19.88)
Austria	3409.34(3015.16-3632.27)	16.78(15.05-17.82)	3268.59(2870.19-3515.65)	15.69(13.94-16.79)
Azerbaijan	1240.9(1002.91-1612.84)	16.49(13.22-21.12)	1217.73(957-1659.4)	15.34(12.08-19.77)
Bahamas	35.32(29.54-41.47)	10.55(8.89-12.35)	36.29(29.59-43.75)	10.34(8.5-12.42)
Bahrain	127.28(109.89-146.18)	34.55(29.54-39.42)	144.7(121.1-168.93)	35.37(30.06-40.52)
Bangladesh	67429.49(54053.92-86502.98)	61.07(48.76-78.27)	71034.02(54260.07-92451.09)	59.65(46.23-77.22)
Barbados	40.07(33.22-46.85)	8.08(6.73-9.42)	39.48(31.3-47.6)	7.52(5.97-9.05)
Belarus	1271.81(1174.27-1375.52)	7.77(7.17-8.42)	1260.92(1045.45-1471.28)	7.72(6.39-9.03)
Belgium	6152.45(5286.9-6627.25)	22.72(19.92-24.28)	5897.83(5006.38-6383.71)	21.42(18.54-22.99)
Belize	52.6(47.43-58.25)	22.02(19.87-24.39)	53.23(45.77-60.47)	20.61(17.68-23.5)
Benin	941.86(720.59-1235.43)	24.37(18.59-31.49)	981.49(741.88-1308.02)	24.12(18.28-31.85)
Bermuda	12.62(10.9-14.68)	8.72(7.56-10.15)	13.42(11.42-16)	8.67(7.39-10.36)
Bhutan	449.22(349.48-582.22)	89.33(69.61-116.6)	464.74(354.1-614.44)	87.61(67.09-116.37)
Bolivia (Plurinational State of)	1907.58(1499.01-2375.73)	27.7(22.01-34.18)	1807.3(1381.89-2333.49)	26.26(20.41-33.45)
Bosnia and Herzegovina	1122.99(971.71-1268.71)	17.3(14.96-19.55)	1057.2(837.8-1279.72)	16.32(12.95-19.8)
Botswana	376.84(302.19-491.77)	36.3(29.34-47.89)	387.7(309-509.34)	35.18(28.01-47.73)
Brazil	62109.09(55669.49-65470.91)	27.62(24.66-29.16)	63673.98(56870.93-67660.39)	26.49(23.59-28.19)
Brunei Darussalam	66.48(57.54-79.23)	38.97(33.42-45.6)	68.8(59.06-81)	33.64(28.55-39.83)
Bulgaria	2288.81(2036.73-2564.84)	15.51(13.87-17.33)	2240.73(1907.53-2605.18)	15.24(13.01-17.69)
Burkina Faso	1328.67(1073.23-1667.73)	18.94(15.37-24.03)	1340.71(1056.73-1678.09)	18.51(14.97-23.72)
Burundi	1251.04(862.57-1612.1)	37.55(26.5-48.45)	1343.2(921.02-1739.88)	37.72(26.31-48.99)
Cabo Verde	68.56(53.23-82.62)	16.85(13.06-20.39)	66.2(49.79-81.09)	16.21(12.25-19.86)
Cambodia	3848.71(3043.6-4707.64)	46.51(37.05-56.06)	4071.36(3184.32-5023.1)	46.08(36.74-55.7)
Cameroon	2331.56(1819.22-3029.38)	25.66(20.3-33.13)	2384.78(1842.26-3129.12)	24.78(19.62-32.22)
Canada	15443.61(13560.98-16581.28)	19.85(17.61-21.24)	15147.24(13099.19-16369.27)	18.46(16.12-19.95)
Central African Republic	839.45(521.89-1223.57)	57.61(34.13-84)	844.76(525.26-1222.66)	55.87(32.76-82.29)
Chad	1357.2(941.34-1767.48)	30.86(21.54-39.48)	1389.02(976.85-1793.69)	30.78(21.93-39.36)
Chile	4325.38(3824.67-4630.41)	17.56(15.53-18.78)	4234.86(3683.07-4567.1)	15.97(13.91-17.2)
China	1200793.29(1020270.99-1414895.54)	74.63(63.54-87.69)	1285433.17(1044727.8-1539819.91)	73.23(59.73-86.85)
Colombia	15941.23(14056.74-16999.95)	29.56(26.31-31.38)	17399.13(14352.79-20325.24)	30.38(25.16-35.39)
Comoros	98.01(68.13-128.84)	26.21(18.29-34.45)	103.11(69.98-136.74)	26.32(18.25-34.84)
Congo	651.8(490.94-862.15)	36.94(28.25-48.83)	674.31(510.7-886.06)	36.18(28.3-47.33)
Cook Islands	5.86(4.87-7.06)	25.41(21.08-30.58)	6.12(4.94-7.55)	24.84(20.09-30.67)
Costa Rica	1116.88(970.87-1202.83)	21.09(18.49-22.64)	1120.83(936.14-1258.99)	19.73(16.61-22.08)
Croatia	1911.01(1715.03-2083.68)	19.2(17.27-20.8)	1947.13(1708.15-2177.53)	19.05(16.72-21.23)
Cuba	4604.96(4285.1-4874.21)	22.99(21.45-24.34)	4669.73(4087.46-5257.03)	22.57(19.78-25.41)
Cyprus	458.02(388.04-528.62)	29.4(25.12-33.9)	460.18(382.78-539.92)	27.02(22.71-31.62)
Czechia	3875.18(3489.23-4213.72)	17.01(15.33-18.54)	3903.45(3355.38-4406.64)	16.6(14.24-18.8)
Cote d'Ivoire	1943.29(1540.62-2453.92)	25.05(20.41-31.11)	2003.76(1563.32-2568.04)	24.43(19.57-30.76)
Democratic People's Republic of Korea	29418.06(21727.2-40845.54)	111.44(82.52-161.22)	30211.24(22332.48-42248.3)	107.68(80.67-156.7)
Democratic Republic of the Congo	11654.56(7256.84-18638.75)	47.31(29.21-79.88)	12256.81(7545.31-19734.58)	47.53(29.56-79.56)
Denmark	4687.15(4136.32-5039.36)	36.37(32.33-38.94)	4821.98(4240.63-5196.95)	35.43(31.37-38.12)
Djibouti	82.56(50.94-121.91)	20.88(13.14-30.19)	87.71(55.22-127.18)	20.48(13.05-29.2)
Dominica	13.56(11.66-16)	17.91(15.39-21.05)	13.91(11.92-16.46)	17.85(15.3-20.95)
Dominican Republic	1164.86(909.43-1630.55)	12.83(10.01-17.96)	1253.51(911.13-1920.48)	13.11(9.55-20.04)
Ecuador	2563.53(2305.56-2820.7)	19.14(17.26-20.98)	2425.05(2005.52-2893.93)	17.43(14.6-20.63)
Egypt	10230.81(9319.31-11256.56)	26.33(23.59-29.17)	10701.81(8682.52-13028.31)	25.87(21.51-31.06)
El Salvador	1218.57(1014.85-1425.91)	17.9(14.98-20.78)	1228.35(962.12-1565.47)	17.68(13.73-22.56)
Equatorial Guinea	88.94(59.16-130.52)	25.84(17.98-37.04)	91.08(61.34-133.52)	25.39(17.97-36.81)
Eritrea	668.13(503.76-867.08)	36.83(28.08-47.34)	707.04(535.24-913.85)	36.51(28.01-46.83)
Estonia	188.45(170.26-202.79)	6.16(5.61-6.63)	192.6(168.09-216.82)	6.13(5.33-6.91)
Eswatini	253.68(186.43-338.81)	59.95(44.22-77.87)	237.48(173.39-314.42)	52.37(38.51-68.74)
Ethiopia	8495.67(6593.88-10149.91)	25.53(20.01-30.34)	8817.41(6883.1-10509.46)	25.4(20.27-30.05)
Fiji	195.17(149.76-242.81)	39.49(31.19-48.55)	200.7(149.17-250.67)	38.5(29.31-47.65)
Finland	1462.13(1285.33-1583.42)	10.21(9.06-10.98)	1503.71(1311.34-1640.96)	9.9(8.74-10.74)
France	16769.32(14319.32-18275.93)	9.37(8.17-10.12)	16593.37(14087.92-18170.9)	8.9(7.7-9.66)
Gabon	203.72(146.5-283.91)	26.96(19.54-38.19)	204.17(146.77-292.27)	26.25(19.14-37.88)
Gambia	242.94(171.31-326.94)	31.04(21.86-42.03)	244.02(168.49-330.75)	30.68(21.08-41.46)
Georgia	714.98(632.54-791.74)	11(9.72-12.15)	700.55(597.21-812.8)	10.97(9.36-12.74)
Germany	40863.67(36295.69-43741.2)	19(17.22-20.16)	40495.89(35692.27-43610.14)	18.28(16.51-19.46)
Ghana	1766.32(1422.6-2176.58)	14.26(11.61-17.46)	1781.76(1402.27-2230.55)	13.85(10.97-17.07)
Greece	4807.64(4163.83-5207.62)	15(13.22-16.11)	4996.07(4291.53-5422.84)	14.98(13.08-16.14)
Greenland	23.84(19.9-28.13)	46.24(37.44-55.42)	23.8(19.69-28.59)	42.98(34.48-52.82)
Grenada	13.54(12.37-14.72)	13.77(12.65-15)	13.88(12.06-15.5)	13.62(11.91-15.08)
Guam	34.67(29.73-39.55)	16.88(14.62-19.22)	29.81(24.93-34.2)	13.64(11.57-15.55)
Guatemala	1715.81(1598.55-1818.93)	21.15(19.63-22.44)	1601.05(1383.44-1813.27)	17.78(15.57-20.01)
Guinea	1369.53(1003.6-1788.14)	29.27(21.4-37.77)	1335.91(985.39-1759.21)	28.71(21.16-37.24)
Guinea-Bissau	195.73(135.21-249.8)	37.48(25.39-47.81)	194.99(133.56-249.47)	36.12(24.36-45.93)
Guyana	78.5(63.35-94.6)	14.67(11.95-17.49)	78.19(61.03-99.32)	14.2(11.19-17.72)
Haiti	1856.78(810.52-2777.72)	36.04(16.42-53.17)	1889.33(834.96-2863.99)	35.24(16.08-53.06)
Honduras	2746.32(2177.18-3423.5)	58.86(46.93-72.94)	2767.08(2171.51-3486.73)	56.63(44.76-70.3)
Hungary	6188.67(5681.72-6605.82)	30.55(28.03-32.66)	5990.33(5208.42-6792.94)	29.25(25.55-33.28)
Iceland	114.85(95.63-128.36)	18.26(15.4-20.28)	113.87(93.62-127.42)	16.87(14.11-18.77)
India	1062605.55(946726.43-1183512.26)	114.37(100.99-127.55)	1066181.25(939670.33-1202912.45)	108.39(94.73-122.39)
Indonesia	80395.86(64852.31-94214.58)	49.76(40.55-57.34)	85259.45(69822.72-101680.42)	49.53(40.95-58.23)
Iran (Islamic Republic of)	11873.27(10466.45-12995.79)	18.79(16.38-20.63)	11440.37(9949.49-12568.57)	17.44(15.06-19.2)
Iraq	1785.3(1384.07-2300.78)	10.34(8.11-13.16)	2006.34(1504.79-2512.34)	11.44(8.67-14.41)
Ireland	2157.58(1856.59-2365.65)	26.92(23.23-29.5)	2003.62(1720.21-2215.53)	23.26(20.03-25.66)
Israel	1657.48(1427.17-1793.3)	12.92(11.19-13.92)	1665.67(1408.03-1821.47)	12.08(10.31-13.15)
Italy	28223.12(23172.83-31015.08)	14.43(12.09-15.73)	28602.39(23503.69-31322.26)	14.18(11.89-15.43)
Jamaica	468.08(376.15-575.04)	14.87(11.93-18.28)	473.55(369.69-592.77)	14.7(11.51-18.47)
Japan	31191.48(25461.72-34086.35)	5.89(4.97-6.37)	32776.95(26625.89-35933.5)	5.84(4.93-6.33)
Jordan	483.78(400.8-591.51)	10.48(8.52-12.8)	562.17(443.08-696.24)	10.73(8.55-13.2)
Kazakhstan	7121.86(6244.43-8065.14)	47.98(42.13-54.05)	7144.87(6148.22-8309.05)	46.68(40.28-54.1)
Kenya	5668.5(3447.56-10018.49)	35.26(20.96-65.39)	5823.53(3524.08-10425.88)	34.58(20.66-64.8)
Kiribati	38.04(27.16-59.18)	84.32(61.6-135.29)	39.56(28.68-62.42)	83.25(62.48-133.85)
Kuwait	50.62(44.31-55.66)	2.72(2.35-3)	56.58(46.4-67.73)	2.74(2.24-3.28)
Kyrgyzstan	1064.42(963.82-1159.96)	29.27(26.34-31.87)	1066.54(896.54-1237.63)	28.25(23.88-32.67)
Lao People's Democratic Republic	1862.35(1344.01-2459.28)	57.31(41.73-74.96)	1945.69(1402.39-2564.42)	56.4(41.34-72.81)
Latvia	303.43(270.56-339.24)	7.01(6.25-7.86)	299.4(254.74-348.52)	6.94(5.9-8.11)
Lebanon	1273.76(1074.13-1488.67)	19.88(16.77-23.21)	1309.43(1074.8-1565.03)	19.65(16.16-23.51)
Lesotho	659.53(444.88-869.35)	74.39(51.34-97.66)	618.2(431.49-818.17)	68.03(48.45-89.97)
Liberia	402.13(296.73-545.02)	26.48(19.59-35.47)	420.66(310.99-576.82)	26.55(19.69-35.93)
Libya	822.15(631.44-1086.3)	20.28(15.55-26.81)	851.02(649.38-1144.31)	19.96(15.43-26.6)
Lithuania	574.1(528.94-616.56)	9.05(8.37-9.69)	569.92(505.94-629.37)	8.9(7.88-9.82)
Luxembourg	229.91(204.47-253.73)	20.42(18.28-22.52)	222.04(194.69-248.85)	18.59(16.43-20.77)
Madagascar	3742.93(2785.48-4988.42)	53.97(40.32-71.18)	3940.52(2948.34-5196.79)	54(40.77-70.83)
Malawi	1642.98(1319.71-2006.73)	29.84(23.82-36.48)	1683.99(1366.71-2085.8)	29.2(23.86-35.62)
Malaysia	6135.56(5352.13-6921.86)	28.55(24.64-32.52)	6779.63(5913.15-7635.36)	28.85(24.9-32.9)
Maldives	85.43(73.29-100.77)	36.44(31.19-43.11)	89.59(73.59-108.47)	35.01(29.07-42.49)
Mali	3080.99(2428.34-3972.4)	45.82(36.51-57.89)	3091.48(2369.81-4071.23)	44.4(35.43-57.08)
Malta	110.77(96.8-122.29)	10.48(9.22-11.58)	110.26(94.94-122.97)	9.74(8.48-10.87)
Marshall Islands	15.01(11.04-19.55)	73.12(54.74-92.34)	15.69(11.66-20.67)	71.32(53.51-90.51)
Mauritania	321.38(249.03-410.29)	19.01(14.78-24.26)	337.38(258.25-430.64)	19.31(14.83-24.42)
Mauritius	255.62(234.94-271.36)	16.75(15.28-17.83)	276.62(252.65-293.91)	16.88(15.32-18.02)
Mexico	29750.24(27800.79-30722.93)	28.4(26.43-29.35)	30707.02(27403.72-33861.86)	27.87(24.9-30.7)
Micronesia (Federated States of)	33.02(25.51-42.18)	68.14(53.5-85.95)	33.9(26.27-44.37)	67.13(53.27-86.18)
Monaco	14.7(11.51-18.02)	12.3(9.8-15.01)	14.67(11.3-18.16)	12.09(9.45-14.93)
Mongolia	320.78(258.09-391.07)	21.28(17.02-25.54)	336.95(268.1-406.93)	20.59(16.43-24.89)
Montenegro	54.34(44.71-64.19)	5.98(4.86-7.11)	52.88(41.69-64.2)	5.87(4.63-7.15)
Morocco	6531.24(4923.24-7997.65)	23.79(18.13-29.02)	6708.77(5244.66-8061.88)	23.52(18.52-28.25)
Mozambique	2358.34(1844.59-2959.23)	29.22(22.42-36.84)	2337.67(1776.18-2918.64)	27.78(21.01-35.02)
Myanmar	38485.28(30409.31-47145.37)	104.65(82.2-127.92)	40096.14(31826.48-48951.32)	104.53(82.46-126.42)
Namibia	561(443.85-707.57)	53.85(42.82-69.66)	559.9(439.92-731.42)	51.42(40.79-68.23)
Nauru	3.1(2.19-4.94)	80.62(56.83-142.82)	3.13(2.18-4.92)	79.33(53.83-140.78)
Nepal	26710.67(21164.81-33199.87)	147.14(116.94-182.44)	27536.39(21511.83-34735.79)	146.13(116.66-182.46)
Netherlands	10337.35(9000.14-11145.67)	27.11(23.78-29.14)	10731.24(9299.26-11612.43)	26.93(23.51-29.07)
New Zealand	1976.97(1727.41-2130.24)	22.88(20.04-24.61)	2032.34(1765.71-2202.43)	21.97(19.19-23.76)
Nicaragua	804.98(688.13-899.3)	21(17.9-23.52)	749.01(618.53-889.09)	18.66(15.18-22.05)
Niger	1617.19(1104.5-2150.94)	28.79(19.44-37.97)	1700.65(1163.36-2280.18)	28.5(18.98-37.47)
Nigeria	12327.09(10275.27-15103.49)	19.1(15.95-22.78)	12570.54(10544.56-15344.33)	18.62(15.79-22.12)
Niue	0.97(0.79-1.19)	48.62(39.21-59.95)	0.97(0.78-1.19)	48.45(39.22-59.64)
North Macedonia	572.95(486.94-682.58)	22.97(19.58-27.34)	559.08(415.95-785.32)	21(16.05-28.34)
Northern Mariana Islands	14.63(12.2-17.01)	46.43(39.55-53.41)	16.28(14.32-18.71)	47.04(40.84-53.5)
Norway	2777.72(2429.12-2961.72)	25.36(22.41-26.94)	2843.89(2475.01-3042.39)	24.65(21.65-26.3)
Oman	183.49(151.55-222.89)	15.36(12.9-18.45)	182.84(147.54-236.7)	14.24(11.62-17.63)
Pakistan	63882.13(51228.97-80375.7)	75.86(61.52-95.5)	64393.12(51864.17-81021.53)	73.8(60.04-94.55)
Palau	9.29(7.68-11.24)	70.67(59.23-83.66)	9.91(8.01-12.09)	70.54(57.96-84.93)
Palestine	265.91(231.56-314.05)	16.1(13.96-19.04)	287.51(243.08-348.24)	16.34(13.91-19.89)
Panama	706.08(618.08-761.36)	16.28(14.3-17.51)	717.13(557.09-856.25)	15.53(12.08-18.57)
Papua New Guinea	4929.67(3918.68-6049.43)	159.04(127.17-193.36)	5186.4(4049.01-6549.73)	156.82(123.55-197.43)
Paraguay	1060.33(877.49-1273.43)	21.09(17.44-25.32)	988.05(787.48-1242.96)	18.7(14.91-23.53)
Peru	3575.07(2675.13-4827.87)	10.68(7.98-14.5)	3158.08(2190.03-4277.52)	9.44(6.51-12.85)
Philippines	21111.98(19108.33-23736.99)	33.72(30.33-38.13)	21865.71(18313.68-25798.62)	32.81(27.69-38.24)
Poland	8884.86(8287.03-9249.29)	11.82(11.07-12.3)	9126.21(8251.25-9904.58)	11.8(10.69-12.81)
Portugal	5646.97(4771.85-6170.32)	18.26(15.57-19.83)	5702.86(4769.37-6270.62)	17.55(14.93-19.18)
Puerto Rico	1498.46(1305.98-1620.77)	17.28(15.41-18.58)	1553.56(1258.09-1821.58)	16.98(14.01-19.81)
Qatar	46.32(36.66-57.9)	10.99(8.6-13.56)	50.9(39.46-65.07)	12.79(9.99-15.94)
Republic of Korea	9744.76(7945.13-12130.84)	11.87(9.67-14.76)	10916.72(8858.9-13478.57)	12.03(9.74-14.78)
Republic of Moldova	694.27(626.59-766.25)	11.7(10.57-12.93)	695(615.5-780.1)	11.54(10.25-12.95)
Romania	6837.9(6325.17-7327.7)	17.51(16.2-18.77)	6865.35(6056.65-7638.59)	17.41(15.34-19.38)
Russian Federation	31954.29(30388.35-32818.37)	13.38(12.73-13.75)	31664.23(29115.3-34367.89)	13.04(12-14.13)
Rwanda	1474.9(1065.05-1937.87)	33.99(24.47-44.75)	1602.66(1091.73-2165.77)	34.47(23.96-46.31)
Saint Kitts and Nevis	8.41(7.39-9.46)	16.63(14.78-18.51)	8.98(7.58-10.31)	16.55(14.25-18.64)
Saint Lucia	51.57(44.6-59.42)	23.45(20.34-26.94)	54.26(44.24-64.22)	23.53(19.22-27.78)
Saint Vincent and the Grenadines	13.9(12.62-15.35)	11.08(10.1-12.21)	14.53(12.84-16.33)	11.15(9.89-12.49)
Samoa	79.52(61.71-98.85)	70.96(54.98-88.16)	80.68(63.62-99.48)	69.57(54.86-85.98)
San Marino	10.09(8.16-12.31)	9.92(8.08-12.17)	6.18(4.33-8.44)	6.05(4.27-8.3)
Sao Tome and Principe	50.11(37.58-61.37)	60.59(44.91-73.33)	50.7(38.3-63.9)	58.43(44.21-72.16)
Saudi Arabia	2622.44(2154.22-3129.4)	23.24(19.35-27.47)	2803.87(2266.24-3415.31)	22.69(18.55-27.31)
Senegal	1584.09(1223.95-2040.82)	26.25(20.45-33.83)	1612.79(1235.86-2068.23)	25.94(20.06-33.05)
Serbia	3472.79(3084.85-3867.54)	20.09(17.83-22.38)	3456.41(2903.89-4065.45)	19.76(16.6-23.28)
Seychelles	24.68(21.65-28)	26.62(23.43-30.23)	23.15(19.9-26.69)	23.68(20.4-27.46)
Sierra Leone	744.71(532.4-1003.55)	25.98(18.71-34.45)	774.58(549.91-1057)	25.64(18.37-34.62)
Singapore	452.96(401.53-486.47)	6.23(5.5-6.69)	488.41(427.77-526.35)	5.93(5.19-6.4)
Slovakia	905.51(793.78-1035.17)	9.68(8.49-11.06)	934.22(784.41-1119.91)	9.62(8.09-11.53)
Slovenia	612.06(541.77-663.6)	11.97(10.66-12.94)	608.81(522.39-671.55)	11.49(9.92-12.67)
Solomon Islands	150.03(118.95-187.4)	67.45(54.67-82.67)	155.98(125.95-193.99)	66.46(54.6-80.63)
Somalia	1822.46(1072-2583.87)	43.72(25.44-61.05)	1848.67(1097.12-2603.03)	41.35(23.88-57.88)
South Africa	13043.42(11996.77-14190.21)	34.52(31.73-37.61)	13322.01(12247.33-14499.68)	34.01(31.2-36.95)
South Sudan	1054.6(699.33-1480.04)	38.13(25.54-52.76)	1069.3(712.78-1493.36)	37.74(25.3-52.04)
Spain	30573.66(26261.85-33363.53)	23.4(20.5-25.41)	29647.46(24925.77-32607.93)	21.98(19-23.95)
Sri Lanka	10300.99(7920.29-13248.44)	49.05(38.04-63.38)	10679.06(7286.32-13972.08)	46.21(31.82-60.72)
Sudan	3910.58(2433.51-5504.49)	25.51(16-35.69)	4013.24(2540.89-5759.74)	25.51(16.28-36.36)
Suriname	98.22(87.41-110.41)	17.61(15.64-19.94)	90.7(69.88-115.62)	15.32(11.86-19.65)
Sweden	3712.14(3262.55-3990.74)	14.53(12.89-15.55)	3601.06(3104.81-3987.76)	13.51(11.69-14.92)
Switzerland	2484.92(2101.37-2728.52)	11.81(10.19-12.91)	2496.43(2092.36-2760.71)	11.31(9.69-12.43)
Syrian Arab Republic	2370.57(1824-2977.67)	26.06(20.02-32.12)	2485.48(1897.51-3161.21)	25.82(20.35-31.73)
Taiwan (Province of China)	7532.97(6733.61-8095.44)	17.78(16.04-19.09)	7796.58(6743.73-8582.28)	17.06(14.87-18.72)
Tajikistan	1157.48(848.45-1572.16)	28.92(20.83-39.11)	1225.5(889.13-1656.75)	29.1(20.8-38.89)
Thailand	19111.64(15658.47-22725.33)	19.26(15.81-22.82)	21391.88(16484.02-27003.5)	19.8(15.22-24.97)
Timor-Leste	335.01(251.79-426.62)	52.23(39.35-65.77)	353.89(262.43-451.61)	51.35(38.52-65.43)
Togo	743.24(553.16-1020.83)	28.26(21.15-38.16)	793.45(584.19-1074.32)	28.22(20.94-38.12)
Tokelau	0.67(0.49-0.95)	45.7(34.17-64.82)	0.67(0.49-0.98)	45.39(33.12-66.2)
Tonga	36.57(30.02-43.67)	49.87(41.04-59.39)	36.64(29.76-44.11)	48.96(39.77-58.69)
Trinidad and Tobago	192.8(168.21-220.86)	10.78(9.42-12.33)	202.95(156.9-251.14)	10.78(8.34-13.35)
Tunisia	1949.43(1335.24-2773.7)	17.84(12.23-25.5)	2021.51(1407.82-2900.02)	17.61(12.17-25.98)
Turkey	33406.55(28638.09-38618.37)	41.96(35.9-48.58)	34044.47(28218.89-40618.51)	40.77(33.86-48.52)
Turkmenistan	373.32(301.94-452.32)	11.09(9.04-13.36)	385.47(301.24-486.52)	11.05(8.76-13.74)
Tuvalu	4.4(3.31-5.68)	56.3(42.51-72.07)	4.47(3.36-5.79)	54.85(41.47-70.2)
Uganda	3161.61(2117.39-4242.42)	29.31(19.78-39.03)	3277.77(2197.48-4445.55)	28.93(19.57-39.04)
Ukraine	7861.49(6751.16-9200.32)	9.97(8.56-11.67)	7471.14(5654.15-9388.64)	9.39(7.1-11.8)
United Arab Emirates	472.4(362-586.19)	38.76(28.9-48.09)	428.24(338.64-532.75)	26.26(18.3-33.46)
United Kingdom	40671.22(36105.45-42843.79)	27.81(24.97-29.16)	39383.25(34801.29-41582.49)	26.22(23.42-27.57)
United Republic of Tanzania	4238.73(3259.05-5325.47)	21.7(16.91-26.99)	4389.73(3347.63-5593.3)	21.64(16.78-27.41)
United States of America	189938.26(166908.35-201549.07)	31.27(27.67-33.06)	198036.53(172625.06-210192.16)	31.32(27.47-33.15)
United States Virgin Islands	15.37(11.92-20.32)	9.09(7.07-11.96)	13.66(10.45-18.16)	7.66(5.88-10.2)
Uruguay	2043.26(1866.91-2162.27)	32.78(30.33-34.61)	2070.56(1890.97-2208.15)	32.36(29.74-34.48)
Uzbekistan	1641.83(1467.72-1826.64)	8.83(7.88-9.86)	1735.94(1475.67-2052.92)	8.75(7.46-10.28)
Vanuatu	95.4(58.51-126.41)	84.85(53.82-114.48)	100.89(63.2-133.23)	83.39(54.05-112.14)
Venezuela (Bolivarian Republic of)	5722.36(4765.47-6977.88)	21.44(17.89-25.99)	6091.31(4713.68-7724.67)	22.12(17.16-27.82)
Viet Nam	34238.71(27792.57-41181.29)	45.08(36.59-54.33)	35336.52(27905.02-43547.67)	43.96(35.11-54.17)
Yemen	3162.8(2299.2-4144.54)	30.7(22.37-40.36)	3373.71(2433.7-4461.99)	31.26(22.66-41.27)
Zambia	1535.71(1182.94-1920.59)	31.23(24.48-38.64)	1535.09(1185.14-1939.56)	29.44(23.12-36.83)
Zimbabwe	1474.68(1141.31-1833.41)	31.21(24.03-38.22)	1453.82(1125.64-1826.89)	28.99(22.37-35.79)

Abbreviations

ASMR, age-standardized mortality rate, COPD: chronic obstructive pulmonary disease.

Table S7 The number of DALYs cases and ASDR of COPD between 2019 and 2021 at the national level.

	2019		2021	
**Location**	**Number**	**ASDR**	**Number**	**ASDR**
Afghanistan	110347.93(83599.97-138541.9)	1164.48(879.59-1479.06)	113688.65(86374.09-144932.17)	1155.17(875.13-1480.07)
Albania	14555.81(12027.51-17927)	349.9(290.94-427.39)	14700.41(11935.5-18668.11)	342.39(280.66-430.36)
Algeria	146017.23(125507.62-169566.96)	489.78(419.91-568.29)	154501.17(131187.84-180952.82)	488.58(415.38-573.16)
American Samoa	417.85(368.01-476.31)	998.71(879.05-1130.87)	430.47(370.97-500.51)	993.97(860.8-1150.4)
Andorra	654.81(525.01-789.78)	448.29(357.73-539.14)	618.2(481.42-766.19)	390.98(303.12-486.99)
Angola	70047.02(56588.59-85875.78)	687.26(555.43-842.26)	75876.13(60578.58-93582.23)	692.53(547.4-863.01)
Antigua and Barbuda	172.11(156.44-189.59)	182.07(165.65-199.94)	181.83(167.49-196.94)	179.35(165.54-194.02)
Argentina	295133.12(276541.33-312152.82)	537.05(504.41-567.89)	276792.75(257234.05-294660.1)	487.71(454.18-519.7)
Armenia	14595.59(13383.37-15835.39)	345.71(317.19-374.16)	14601.17(13080.16-16353.25)	339.94(305.43-379.96)
Australia	169599.48(154328.52-182304.66)	381.06(349.16-409.53)	174320.88(157434.8-186600.77)	365.77(332.29-390.19)
Austria	73537.97(67698.53-79360.18)	404.44(373.66-435.92)	70997.95(64998.03-76794.7)	381.09(350.49-411.09)
Azerbaijan	34337.25(29088.02-43065.19)	391.1(332.69-485.11)	35021.69(28521-47582.76)	374.49(309.17-479.57)
Bahamas	900.35(770.8-1035.04)	243.1(208.43-278.52)	931.93(776.01-1094.21)	239.25(201.04-280.35)
Bahrain	4113.88(3599.75-4594.72)	671.06(589.1-754.37)	4624.6(3987.56-5251.8)	681.46(591.17-765.26)
Bangladesh	1628846.51(1329412.28-1991745.59)	1327.58(1086.93-1630.45)	1713712.75(1376728.77-2133832.83)	1301.68(1050.53-1612.22)
Barbados	889.45(759.61-1030.61)	181.74(155.79-210.38)	885.27(731.51-1039.46)	171.75(142.62-201.16)
Belarus	38384.22(35145.03-41875.44)	245.21(223.98-267.59)	38503.44(33454.47-44553.71)	245.19(212.95-282.99)
Belgium	117975.34(106814.81-126775.94)	498.95(458.09-536.04)	115161.65(103933.99-123893.68)	477.58(437.24-512.88)
Belize	1252.24(1142.48-1380.33)	469.74(427.65-516.45)	1286.07(1136.05-1457.45)	444.57(391.58-501.54)
Benin	30080.17(24705.63-36932.12)	605.88(496.4-748.29)	31985.46(26021.06-39965.07)	604.11(487.65-760.4)
Bermuda	265.24(232.95-303.52)	199.63(174.46-227.74)	278.32(240.64-322.83)	198.74(171.34-230.34)
Bhutan	9265.26(7302.1-11852.41)	1710.29(1357.97-2174.16)	9571.4(7479.21-12335.71)	1678.89(1317.32-2166)
Bolivia (Plurinational State of)	38281.36(31152.79-46961.88)	485.2(394.63-592.63)	37740.33(30140.7-47632.14)	470.77(376.96-590.85)
Bosnia and Herzegovina	26416.9(23680.34-29444.42)	422.02(378.55-469.56)	25257.6(21127.24-29835.25)	403.71(338.66-474.43)
Botswana	10933.07(9216.42-13268.65)	850.78(713.86-1056.04)	11491.82(9596.66-14337.45)	840.65(700.98-1049.95)
Brazil	1322343.33(1233213.75-1390256.67)	568.97(528.63-599.01)	1380988.99(1280462.39-1456315.1)	558.69(517.05-588.99)
Brunei Darussalam	1515.21(1335.45-1744.58)	654(577.4-758.39)	1588.86(1395.68-1833.67)	595.82(519.54-686.73)
Bulgaria	55880.39(50879.69-62056.01)	397.55(361.2-439.49)	54940.8(47895.68-62497.55)	394.48(344.83-447.94)
Burkina Faso	44844.06(38434.77-52790.41)	498.86(429.69-592.17)	46286.21(39032.65-55062.03)	491.7(416.02-586.83)
Burundi	38421.24(28340.3-47032.75)	871.76(642.1-1070.11)	41563.16(30723.08-51254.7)	877.53(646.02-1091.66)
Cabo Verde	1684.69(1403.93-1976.03)	395.58(329.39-465.29)	1713.77(1397.49-2049.25)	388.2(317.27-462.46)
Cambodia	97081.83(79561.02-116521.71)	942.63(778.49-1114.82)	102748.71(83618.21-124864.99)	935.16(771.66-1124.75)
Cameroon	76764.85(62444.15-94475.21)	633.16(517.87-777.55)	80328.01(65856.81-99654.78)	620.09(506.5-770.63)
Canada	302988.15(276966.31-324659.58)	418.28(383.59-448.35)	299697.42(274496.96-323214.33)	393.18(360.94-423.66)
Central African Republic	26480.74(18261.76-36450.37)	1327.62(875.7-1838.15)	27118.88(18600.06-37062.61)	1302.89(863.86-1797.34)
Chad	43095.48(32962.76-53443.86)	761.11(579.03-945.55)	45369.09(34999.83-55823.32)	763.57(583.64-942.36)
Chile	74044.36(67935.34-78842.68)	304.03(279.41-323.66)	73317.53(66461.85-78458.21)	282.02(256.06-301.72)
China	22337597.44(19180507.73-26139106.45)	1251.83(1077.58-1461.13)	23640320.96(19998658.36-27921931.16)	1227.66(1048.45-1442.54)
Colombia	283182.06(259837.01-300733.64)	538.04(496.17-571.44)	312592.99(266418.63-357444.81)	559.45(477.79-640.18)
Comoros	2716.87(2066.74-3391.94)	609.73(465.48-763.58)	2883.65(2133.01-3608.71)	615.46(455.41-774.81)
Congo	19529.75(15334.56-24954.09)	834.46(667.13-1057.79)	20598.91(16342.06-26280.82)	825.18(673.44-1037.16)
Cook Islands	129(110.96-150.25)	544.75(470.48-633.76)	133.52(112.41-157.9)	534.64(449.7-630.68)
Costa Rica	20172.44(18423.09-21735.34)	389.9(357.03-419.52)	20986.03(18547.6-23194.02)	378.84(336.42-418.37)
Croatia	38287.12(35269.27-41381.76)	414.88(383.61-447.01)	38588.4(34658.96-42884.58)	410.22(367.21-456.28)
Cuba	98192.51(92548.86-103943.68)	514.92(485.75-544.47)	98637.54(87754.12-109681.85)	501.11(445.8-556.48)
Cyprus	8642.64(7568.01-9816.01)	490.93(433.19-552.98)	8663.62(7456.21-10028.55)	455.12(393.6-521.74)
Czechia	90265.58(82714.05-97904.87)	422.37(388.23-458.9)	90888.69(81256.94-101422.94)	414.71(371.23-462.55)
Cote d'Ivoire	67267(55211.48-80593.9)	626.49(518.06-756.43)	70462.55(57967.06-86542.61)	617.34(510.65-756.38)
Democratic People's Republic of Korea	592924.72(452719.91-767868.12)	2032.26(1556.85-2690.08)	602745.68(459884.79-787221.04)	1968.2(1500.48-2612.25)
Democratic Republic of the Congo	341225.94(237243.78-488736.76)	1067.33(726.69-1604.67)	363380.85(246025.18-522318.11)	1073.41(720.28-1615.37)
Denmark	84390.03(77145.69-90575.21)	705.41(649.06-754.32)	85639.25(78029.01-91949.74)	682.66(625.85-729.99)
Djibouti	2851.48(2041.93-3901.79)	510.91(363.9-708.4)	3067.49(2235.36-4106.62)	506.44(362.23-680.9)
Dominica	296.38(260.01-346.22)	374.05(329.33-435.82)	305.68(265.32-359.75)	373.21(326.91-438.9)
Dominican Republic	27743.07(22655.72-36359.29)	293.4(239.33-383.79)	29187.99(22808.2-40487.7)	294.88(229.69-409.07)
Ecuador	45915.69(41270.88-50618.82)	315.93(284.91-346.95)	45176.7(38805.05-52748.14)	296.86(256.18-345.1)
Egypt	323335.84(299524.6-353563.17)	606.42(560.67-664.21)	340087.26(288662.45-397678.49)	602.17(511.84-700.27)
El Salvador	23173.65(20366.17-26098.61)	365.21(321.4-410.33)	23560.17(19181.61-28573.61)	361.12(294.3-437.05)
Equatorial Guinea	2854.17(2129.53-3812.97)	616.99(462.08-827.43)	3021.16(2260.61-4048.43)	613.98(458.71-833.3)
Eritrea	21254.92(16871.31-26602.76)	847.68(675.41-1052.86)	22655.8(18134.25-28098.96)	844.47(676.17-1047.84)
Estonia	4721.01(4330.52-5163.45)	175.04(160.16-191.35)	4676.34(4161.74-5224.79)	170.09(151.14-190.77)
Eswatini	7278.58(5521.4-9508.92)	1369.42(1045.24-1753.91)	7013.86(5354.04-9104.71)	1264.8(979.92-1626.08)
Ethiopia	256199.35(204632.44-301591.9)	616.85(496.26-722.76)	270087.37(220925.22-310041.64)	619.18(510.02-708.79)
Fiji	5072.69(4046.34-6186.72)	791.61(639.95-944.55)	5203.44(4084.03-6385.25)	778.59(613.56-940.77)
Finland	33421.17(30465.22-36408.79)	264.58(241.77-287.38)	34286.16(31242.55-37492.78)	259.79(237.73-283.43)
France	318745.96(285228.78-349504.73)	217.28(197.94-237.92)	316491.39(282301.15-347195.19)	208.64(188.85-228.62)
Gabon	5856.12(4535.7-7605.59)	626.63(486-818.28)	6009.93(4669.7-7929.73)	616.69(481.4-822.05)
Gambia	7255.27(5635.48-9223.36)	750.22(583.73-967.21)	7467.01(5740.2-9533.42)	744.46(564.13-962.59)
Georgia	17976.96(16097.76-19708.25)	298.58(267.97-327.19)	17653.9(15566.54-19938.19)	295.55(261.43-333.88)
Germany	874859.46(802421.62-939668.98)	458.14(425.27-490.76)	866758.29(790703.65-929152.32)	442.86(409.16-475.53)
Ghana	68625.36(58616.86-80689.87)	419.26(358.29-488.14)	71052.36(59466.21-83837.69)	411.82(344.86-486)
Greece	99064.81(88741.86-109595.43)	380.8(343.11-423.23)	101175.18(90603.64-112074.91)	380.35(340.27-424.19)
Greenland	590.66(516.08-675.12)	963.7(828.3-1116.56)	589.97(509.27-685.27)	907.57(779.86-1067.9)
Grenada	336.11(306.53-366.64)	313.19(287.82-340.55)	343.63(303.55-383.28)	310.32(274.6-343.63)
Guam	913.35(816.53-1015.26)	459.71(410.88-510.62)	847.15(749.83-943.36)	404.98(359.51-450.33)
Guatemala	36334.84(34069.62-39034.46)	382.37(357.52-409.72)	35918.19(31597.92-40508.65)	348.63(307.48-392.08)
Guinea	40361.82(32273.39-49937.36)	720.19(571.34-890.55)	40917.5(32166-51029.82)	713.48(558.98-891.5)
Guinea-Bissau	6612.76(4929.23-8203.21)	908.57(669.03-1114.21)	6749.07(5046.33-8375.64)	887.63(660.38-1092.06)
Guyana	2091.36(1708.24-2535.81)	342.49(282.57-412.21)	2116.02(1706.98-2641.74)	336.02(271.46-415.63)
Haiti	46800.64(22375-69423.36)	738.9(360.58-1074.16)	48350.73(23389.99-72580.24)	728.06(356.16-1071.15)
Honduras	58926.09(48121.62-72497.83)	1081.06(886.04-1324.31)	60342.94(48626.42-74461.23)	1049.64(846.1-1295.33)
Hungary	143543.36(133116.84-153733.49)	765.05(709.66-819.74)	140484.48(124620.13-157381.12)	744.21(658.45-834.29)
Iceland	2363.5(2108.81-2602.15)	414.05(371.87-453.98)	2409.57(2131.96-2661.81)	397.5(353.63-437.69)
India	23842302.47(21517259.42-26249174.72)	2274.65(2050.21-2504.65)	24018380.31(21633268.17-26798623.96)	2171.16(1953.69-2422.39)
Indonesia	2105801.28(1761996.77-2440811.45)	1044.6(871.88-1197.15)	2235813.86(1869853.33-2637091.6)	1040.41(874.72-1216)
Iran (Islamic Republic of)	317282.38(291980.08-343105.08)	448.55(410.87-485.8)	314576.61(285133.04-342208.92)	424.08(383.16-462.24)
Iraq	66865.14(55453.18-80299.13)	304.36(254.93-365.42)	73090.57(60324.22-86600.25)	320.73(262.89-381.13)
Ireland	38972.65(35218.5-42280.51)	505.94(458.91-548.21)	37059.92(33220.9-40422.08)	451.69(406.89-492.35)
Israel	36711.06(32996.13-39761.06)	308.7(278.66-334.87)	37085.7(33148.79-40842.32)	292.37(263.81-320.7)
Italy	477597.94(419062.42-521216.18)	290.29(258.86-314.95)	481343.52(420946.78-524958.28)	285.38(256.09-309.66)
Jamaica	10229.5(8341.31-12327.68)	337.97(274.79-408.39)	10489.15(8496.03-12940.67)	335.42(271.26-413.45)
Japan	659490.79(577376.75-734340.28)	157.67(140.11-176.13)	678591.72(590707.73-756041.37)	155.76(137.62-174.26)
Jordan	19301.41(16820.51-21926.96)	306.14(267.26-349.77)	22173.88(19008.79-25637.79)	311.01(266.39-360.78)
Kazakhstan	174490.19(154380.64-195729.37)	1052.24(933.78-1180.51)	176122.35(153267.75-202154.27)	1023.13(889.04-1171.56)
Kenya	160151.17(110651.27-251725.6)	778.07(520.74-1270.69)	166807.76(115815.95-257071.4)	766.86(514.09-1250.53)
Kiribati	1064.21(784.67-1541.86)	1708.02(1273.56-2565.18)	1102.82(817.32-1676.57)	1684.31(1265.06-2595.52)
Kuwait	4486.96(3847.58-5200.64)	158.68(137.26-182.8)	5039.7(4286.75-5886.41)	160.46(138.49-185.65)
Kyrgyzstan	27294.7(24977.75-29515.96)	641.26(585.97-692.46)	28170.66(24378.1-32270.68)	627.15(542.74-714.78)
Lao People's Democratic Republic	47289.03(35551.5-61270.28)	1192.81(910-1528.11)	49655.06(37453.62-63797.75)	1176.83(892.84-1502.07)
Latvia	8305.27(7528.99-9265.32)	214.11(194.45-238.06)	8287.59(7252.24-9468.67)	214.69(185.89-243.6)
Lebanon	29184.22(25745.59-33022.28)	481.73(425.27-544.86)	30023(26252.86-34370.41)	477.84(416.89-545.89)
Lesotho	17896.02(12854.84-23141.45)	1695.15(1224.73-2170.59)	17137.39(12499.11-22262.12)	1616.31(1182.32-2071.61)
Liberia	13168.41(10641.95-16812.22)	645.44(513.55-814.95)	14129.41(11201.58-18062.36)	652.08(513.7-830.56)
Libya	24928.53(19801.16-30513.02)	511.5(412.27-629.01)	26120.49(21181.6-32666.35)	504.59(410.08-628.23)
Lithuania	14146.03(13080.64-15316.16)	251.3(233.15-271.05)	14095.09(12524.64-15466.19)	249.92(222.79-275.46)
Luxembourg	4444.2(4074.87-4850.26)	428.2(392.54-467.11)	4397.48(3974.31-4849.88)	399.59(360.41-442.07)
Madagascar	112663.02(88774.95-144504.17)	1145.44(893.31-1481.57)	120101.62(92583.3-153445.58)	1147.8(892.85-1466.97)
Malawi	49933.06(41402.63-59419.59)	711.54(589.88-844.57)	52175.42(43677.18-62489.71)	704.6(592.39-839.1)
Malaysia	155020.87(140079.19-168612.8)	623.06(561.6-681.57)	167980.37(151924.5-184807.48)	628.09(565.4-692.06)
Maldives	1992.94(1754.86-2255.22)	716.86(627.28-806.53)	2125.86(1828.09-2479.23)	696.35(597.42-814.59)
Mali	91060.38(74424.18-116448)	1060.07(872.5-1330.2)	94056.55(75273.83-120846.27)	1042.23(837.23-1324.98)
Malta	2463.55(2222.47-2754.51)	256.52(232.12-286.09)	2474.95(2214.19-2771.39)	244.56(218.62-274.13)
Marshall Islands	451.82(341.61-582.97)	1506.28(1148.28-1903.84)	465.72(359.14-605.35)	1467.49(1126.82-1862.39)
Mauritania	9693.74(8028.28-11437.81)	473.86(390.1-562.3)	10337.01(8481.69-12262.52)	482.86(395.79-574.92)
Mauritius	6292.75(5850.26-6771.61)	386.31(359.36-415.17)	6730.16(6244.76-7258.29)	388.62(361.01-420.1)
Mexico	588219.75(558950.86-614723.43)	523.26(495.62-546.61)	615402.85(555650.05-675742.29)	519.67(469.83-570.36)
Micronesia (Federated States of)	912.2(724.68-1162.2)	1417.15(1135.92-1773.87)	935.2(743.76-1205.61)	1396.63(1121.89-1761.24)
Monaco	298.2(254.31-345.33)	296.51(254.06-340.51)	299.33(249.28-351.2)	292.09(246.1-339.51)
Mongolia	9120.32(7563.95-10718.93)	472.98(393.62-556.27)	9333.9(7792.55-10937.98)	453.68(376.58-532.25)
Montenegro	1801.71(1562.66-2072.37)	192.04(166.9-220.8)	1760.15(1508.46-2053.29)	187.53(161.34-217.71)
Morocco	174573.86(139785.98-205428.35)	560.34(450.73-659.64)	181710.71(149072-210580.28)	557.91(461.15-645.66)
Mozambique	75081.28(61106.62-91371.3)	706.48(577.59-862.95)	76327.73(61074.42-91790.39)	683.39(549.98-823.52)
Myanmar	823001.42(679721.71-994268.66)	1967.94(1608.17-2381.75)	860214.53(707432.67-1037228.73)	1958.92(1604.09-2354)
Namibia	14990.35(12152.83-18523.17)	1203.61(974.72-1484.89)	15260.16(12270.42-18867.61)	1171.77(951.35-1457.16)
Nauru	86.41(63.89-116.14)	1650.29(1211.91-2513.42)	86.79(64.11-116.63)	1614.56(1176.72-2426.49)
Nepal	591557.21(471406.81-724584.79)	2863.99(2307.01-3498.67)	610592.94(484943.13-752714.44)	2836.01(2275.31-3485.04)
Netherlands	198163.06(179920.52-211618.83)	563.46(516.91-600.87)	197388.82(179645.47-212040.88)	537.79(493.18-576.92)
New Zealand	37081.42(33438.74-39488.69)	448.55(406.57-476.16)	37935.6(34308.49-40625.61)	431.82(393.22-462.12)
Nicaragua	17612.4(15961.79-19325.18)	411.17(368.53-452.22)	17250.74(14907.44-19802.55)	381.7(327.58-437.44)
Niger	54081.5(40862.52-67320.17)	705.41(521.88-883.3)	57550.23(43963.25-71564.15)	702.13(524.46-871.29)
Nigeria	424384.22(362855.97-492005.94)	496.8(426.18-577.69)	444788.06(382136.52-518374.1)	490.54(424.15-569.06)
Niue	20.17(16.63-24.48)	979.26(807.19-1189.97)	20.38(16.72-24.68)	985.74(811.57-1191.85)
North Macedonia	13820.75(12101.58-15997.97)	471.88(415.1-542.6)	13878.34(10924.74-18496.81)	454.42(362.35-594.99)
Northern Mariana Islands	385.74(328.97-452.31)	928.18(800.06-1071.65)	419.6(369.71-479.8)	940.6(840.4-1055.79)
Norway	51799.01(47460.13-55209.59)	514.56(475.09-548.72)	52691.26(48028.51-56308.12)	497.3(456.34-532.24)
Oman	7036.2(6187.35-8147.59)	405.68(353.34-469.21)	7342.67(6306.72-8827.19)	380.91(326.15-458.22)
Pakistan	1632527.4(1343227.55-1952716.95)	1593.31(1322.05-1924.74)	1657341.7(1374667.51-1999430.95)	1541.67(1287.52-1855.86)
Palau	232.82(194.51-278.35)	1342.1(1137.84-1581.36)	247.74(202.84-295.83)	1338.13(1120.27-1589.76)
Palestine	8657.04(7764.49-9695.79)	394.03(352.16-442.45)	9335.17(8315-10596.72)	397.17(353.23-454.73)
Panama	13467.04(12336.85-14518.98)	318.93(292.29-343.59)	14074.36(11594.61-16339.78)	313.24(258.1-363.23)
Papua New Guinea	124528.88(99886.71-152259.04)	3050.8(2464.16-3687.77)	131563.96(102978.84-164213.6)	3004.36(2404.29-3732.82)
Paraguay	22022.21(18570.12-25913.5)	415.01(349.99-487.21)	21601.88(17738.49-26397.45)	385.23(315.58-470.01)
Peru	71902.54(58588.3-89871.76)	219.07(177.8-274.36)	67067.86(51968.28-85453.23)	200.04(154.29-255.74)
Philippines	611557.74(560773.52-668654.81)	827.4(757.87-906.5)	632847.53(553583.65-728840.2)	807.05(707.12-923.78)
Poland	231999.69(216258.21-250095.68)	328.88(305.8-355.84)	237264.77(216345.67-259512.32)	327.79(298.61-358.48)
Portugal	94472.56(83995.2-102310.34)	355.03(321.55-383.99)	95187.06(85034.21-103420.94)	344.54(312.27-373.1)
Puerto Rico	26382.46(24210.71-28262.07)	359.06(333.66-383.18)	27122.97(23309.6-31270.67)	354.55(305.58-406.86)
Qatar	3052.24(2581.47-3560.33)	320.24(276.45-368.92)	3225.91(2703.08-3773.31)	336.21(287.54-395.65)
Republic of Korea	251870.4(218053.05-294452.39)	295.03(254.34-344.72)	266056.06(230704.14-309042.98)	289.13(251.75-335.7)
Republic of Moldova	18295.6(16546.51-20133.47)	317.35(287.75-349.26)	18449.73(16631.28-20505.4)	314.75(283.83-348.95)
Romania	166001.74(153819.49-176127.28)	461.33(428.8-488.99)	165985.4(150232.73-181410.83)	459.19(415.1-503.41)
Russian Federation	828271.24(787107.52-869721.81)	357.1(339.46-375.21)	828949.94(770974.48-896492.56)	352.01(328.27-379.88)
Rwanda	44675(34466.54-55614.94)	788.52(609.92-988.03)	48502.91(36946.07-61939.8)	800.01(611.66-1028.73)
Saint Kitts and Nevis	204.64(182-229.94)	346.89(311.29-385.43)	220.2(188.1-251.97)	345.5(297.19-389.88)
Saint Lucia	1092.44(949.81-1250.95)	486.82(424.32-556.64)	1151.43(956.25-1360.8)	487.49(404.87-574.54)
Saint Vincent and the Grenadines	316.95(290.28-348.34)	239.54(219.77-262.37)	331.83(298.82-370.36)	240.14(216.88-267.42)
Samoa	1851.18(1478.35-2269.25)	1421.99(1138.7-1744.7)	1884.73(1526.06-2351.12)	1396.39(1123.6-1732.57)
San Marino	193.7(167.06-223.01)	239(206.27-273.41)	148.42(118.97-181.07)	183.81(148.11-222.92)
Sao Tome and Principe	1315.77(1048.05-1577.65)	1302.05(1023.89-1550.91)	1366.83(1089.97-1689.46)	1269.66(1005.65-1563.85)
Saudi Arabia	96972.11(81986.37-113808.6)	540.67(467.84-623.59)	105295.17(87333.97-125544.82)	533.08(452.57-618.8)
Senegal	47318.44(39101.27-57238.77)	637.47(526.65-772.17)	48849.43(39869.59-58522.43)	632.07(513.66-758.44)
Serbia	79476.68(72285.31-87401.04)	480.31(437.31-529.63)	79310.95(67838.9-91575.57)	475.15(406.91-548.67)
Seychelles	613.49(550.39-679.01)	597.14(537.53-659.84)	586.31(520.17-655.93)	538.59(475.57-600.26)
Sierra Leone	23719.07(18516.86-30273.86)	648.94(503.45-827.33)	25126.53(19799.86-32414.88)	646.88(505.84-831.93)
Singapore	11610.79(10487.09-12791.77)	153.13(138.45-168.62)	12295.81(11019.29-13602.4)	146.48(131.42-161.67)
Slovakia	24981.06(22587.3-27858.41)	273.22(247.14-304.53)	25679.33(22461.25-29827.12)	272.01(238.55-314.65)
Slovenia	13051.05(11846.96-14095.9)	288.5(262.99-312.2)	12947.44(11556.89-14366.26)	277.59(245.36-308.42)
Solomon Islands	4095.89(3317.75-5044.31)	1390.07(1143.06-1677.7)	4272.6(3482.75-5280.66)	1368.16(1142.77-1653.18)
Somalia	62243.96(42527.49-83575.58)	1066.69(702.54-1420.18)	64554.17(44390.96-85201.84)	1040.23(683.92-1374.61)
South Africa	367190.91(339594.8-399797.27)	853.35(791.47-926.75)	382073.36(354297.22-418560.81)	853.41(789.28-935.98)
South Sudan	30286.89(21703.98-40620.18)	860.34(613.48-1153.25)	31764.72(22898.62-42331.97)	863.67(616.81-1149.37)
Spain	475266.46(425131.47-513648.55)	430.3(391.22-465.36)	464294.78(411417.53-504946.94)	410.16(370.27-444.09)
Sri Lanka	212372.76(169355.11-264090.11)	906.45(721.75-1128.03)	219257.46(158404.22-280074.74)	864.19(631.53-1103.11)
Sudan	118905.81(82208.84-158448.42)	623.59(433.32-834.5)	124034.06(87671.12-168666.28)	623.17(442.16-841.65)
Suriname	2326.18(2098.26-2570.28)	394.76(357.45-437.25)	2212.57(1777.93-2703.1)	355.3(284.8-434.64)
Sweden	80548.07(73244.48-87992.77)	360.64(329.71-392.99)	78587.41(69696.62-86779.53)	339.36(304.58-374.57)
Switzerland	54665.4(49059.42-60007.79)	300.03(270.12-330.23)	54985.02(48849.93-60797.14)	289.27(259.16-319.86)
Syrian Arab Republic	69318.99(55912.93-84010.21)	608.04(496.12-726.86)	73016(58644.53-89317.53)	605.91(498.55-740.44)
Taiwan (Province of China)	143661.33(132211.88-155831.42)	355.04(326.74-385.41)	148677.51(134659.01-163582.95)	343.42(311.47-377.54)
Tajikistan	31787.92(25196.95-40629.99)	633.95(501.02-819.35)	34175.52(26488.69-44558.64)	637.12(492.25-826.72)
Thailand	451566.53(392040.86-517002.81)	456.65(397.21-522.28)	487665.25(401670.66-595684.92)	461.07(381.24-559.88)
Timor-Leste	8031.26(6270.46-9860.17)	1058.02(828.17-1294.87)	8459.53(6572.16-10395.08)	1047.94(814.39-1288.81)
Togo	25340.95(20204.92-32096.17)	705.59(563.79-898.8)	27088.28(21352.48-34667.65)	705.07(558.67-907.89)
Tokelau	13.28(10.37-17.22)	913.85(713.84-1184)	13.64(10.38-18.29)	921.21(704.89-1227.57)
Tonga	773.17(641.39-914.33)	1005.14(835.85-1184.64)	776.14(640-914.77)	988.89(814.24-1161.4)
Trinidad and Tobago	4687.63(4142.48-5290.36)	255.71(226.59-288.41)	4914.08(4008.45-5932.02)	256.19(210.35-308.6)
Tunisia	55739.21(44805.69-70287.38)	464.59(371.84-588.8)	58175.82(46751.59-74000.97)	460.89(370.65-585.52)
Turkey	736117.58(655372.96-833883.86)	868.1(772.27-980.72)	761035.41(651582.72-881753.18)	850.19(727.99-983.51)
Turkmenistan	12083.26(10106.05-14272.84)	308.24(259.32-360.7)	12562.38(10325.25-15261.61)	307.79(254.46-369.5)
Tuvalu	108.59(83.86-137.31)	1164.71(906.14-1462.33)	110.33(85.61-139.32)	1138.27(890.67-1437.52)
Uganda	96428.11(70716.05-122514.51)	697.06(510.73-883.47)	101881.68(75092.65-128956.84)	693.4(506.33-880.76)
Ukraine	214908.57(187221.96-248047.1)	285.77(248.67-330.34)	207495.33(169403.81-253607.89)	274.39(225.05-334.21)
United Arab Emirates	24116.17(20189.86-28445.52)	794.24(649.26-942.62)	24096.07(20567.65-27878.77)	594.58(488.75-713.85)
United Kingdom	793286.78(735982.72-833784.2)	601.36(561.05-630.95)	774611.97(716224.9-816863.81)	571.45(532.64-601.96)
United Republic of Tanzania	130885.67(105166.28-159890.79)	541.2(434.67-650.58)	138364.36(113030.63-167939.79)	543.49(445.23-659.63)
United States of America	4471175(4161279.38-4726985.63)	780.32(728.06-823.72)	4646118.12(4302650.44-4904223.76)	777.93(725.17-819.94)
United States Virgin Islands	356.65(295.34-437.6)	207.08(171.51-253.41)	324.35(264.21-398.89)	182.91(149.95-224.07)
Uruguay	36069.88(33822.51-38016.47)	649.6(611.12-683.36)	36505.19(33925.34-38640.38)	642.28(600.48-679.05)
Uzbekistan	54367.68(49032.23-59866.42)	240.01(217.33-264.33)	57699.91(49662.94-65971.16)	238.39(206.48-271.83)
Vanuatu	2613.3(1661.57-3421.03)	1730.58(1129.71-2257.17)	2767.2(1799.96-3606.61)	1709.11(1132.62-2218.1)
Venezuela (Bolivarian Republic of)	124752.65(106208.64-147768.77)	447.95(382.3-528.26)	133543.28(106608.74-166502.8)	462.77(370.7-574.17)
Viet Nam	742678.91(609205.77-872850.54)	880.72(726.48-1033.17)	776752.44(626580.15-929720.03)	863.32(702.77-1024.54)
Yemen	94137.67(73392.08-118620.85)	718.67(557.61-898.3)	101184.24(79276.69-127402.3)	730.21(567.09-920.09)
Zambia	47001.56(37622.56-57301.91)	726.9(586.57-879.33)	48749.15(39118.55-59593.62)	705.51(572.04-851.09)
Zimbabwe	46061.86(37760.53-54756.72)	739.92(603.4-873.68)	47044.42(38407.65-56970.44)	721.09(593.96-856.23)

Abbreviations
DALYs, disability-adjusted life years; ASDR, age-standardized DALYs rate, COPD: chronic obstructive pulmonary disease.

STROBE Statement—checklist of items that should be included in reports of observational studies

	Item No	Recommendation	Page
**Title and abstract**	1	(*a*) Indicate the study's design with a commonly used term in the title or the abstract	1
		(*b*) Provide in the abstract an informative and balanced summary of what was done and what was found	1-2
**Introduction**			
Background/rationale	2	Explain the scientific background and rationale for the investigation being reported	3-4
Objectives	3	State specific objectives, including any prespecified hypotheses	4
**Methods**			
Study design	4	Present key elements of study design early in the paper	4
Setting	5	Describe the setting, locations, and relevant dates, including periods of recruitment, exposure, follow-up, and data collection	4-5
Participants	6	(*a*) *Cohort study*—Give the eligibility criteria, and the sources and methods of selection of participants. Describe methods of follow-up<br>*Case-control study*—Give the eligibility criteria, and the sources and methods of case ascertainment and control selection. Give the rationale for the choice of cases and controls<br>*Cross-sectional study*—Give the eligibility criteria, and the sources and methods of selection of participants	4-5
		(*b*) *Cohort study*—For matched studies, give matching criteria and number of exposed and unexposed<br>*Case-control study*—For matched studies, give matching criteria and the number of controls per case	
Variables	7	Clearly define all outcomes, exposures, predictors, potential confounders, and effect modifiers. Give diagnostic criteria, if applicable	4-6
Data sources/ measurement	8*	For each variable of interest, give sources of data and details of methods of assessment (measurement). Describe comparability of assessment methods if there is more than one group	4-6
Bias	9	Describe any efforts to address potential sources of bias	5-6
Study size	10	Explain how the study size was arrived at	4-5
Quantitative variables	11	Explain how quantitative variables were handled in the analyses. If applicable, describe which groupings were chosen and why	4-5
Statistical methods	12	(*a*) Describe all statistical methods, including those used to control for confounding	5-6
		(*b*) Describe any methods used to examine subgroups and interactions	5-6
		(*c*) Explain how missing data were addressed	5-6
		(*d*) *Cohort study*—If applicable, explain how loss to follow-up was addressed<br>*Case-control study*—If applicable, explain how matching of cases and controls was addressed<br>*Cross-sectional study*—If applicable, describe analytical methods taking account of sampling strategy	5-6
		(*e*) Describe any sensitivity analyses	5-6

Continued on next page

**Results**			**Page**
Participants	13*	(a) Report numbers of individuals at each stage of study—eg numbers potentially eligible, examined for eligibility, confirmed eligible, included in the study, completing follow-up, and analysed	6
		(b) Give reasons for non-participation at each stage	Not Applicable
		(c) Consider use of a flow diagram	Not Applicable
Descriptive data	14*	(a) Give characteristics of study participants (eg demographic, clinical, social) and information on exposures and potential confounders	6
		(b) Indicate number of participants with missing data for each variable of interest	Not Applicable
		(c) *Cohort study*—Summarise follow-up time (eg, average and total amount)	
Outcome data	15*	*Cohort study*—Report numbers of outcome events or summary measures over time	
		Case-control study—Report numbers in each exposure category, or summary measures of exposure	
		Cross-sectional study—Report numbers of outcome events or summary measures	*6-9*
Main results	16	(*a*) Give unadjusted estimates and, if applicable, confounder-adjusted estimates and their precision (eg, 95% confidence interval). Make clear which confounders were adjusted for and why they were included	6-9
		(*b*) Report category boundaries when continuous variables were categorized	6-9
		(*c*) If relevant, consider translating estimates of relative risk into absolute risk for a meaningful time period	6-9
Other analyses	17	Report other analyses done—eg analyses of subgroups and interactions, and sensitivity analyses	6-9
**Discussion**			
Key results	18	Summarise key results with reference to study objectives	10
Limitations	19	Discuss limitations of the study, taking into account sources of potential bias or imprecision. Discuss both direction and magnitude of any potential bias	11
Interpretation	20	Give a cautious overall interpretation of results considering objectives, limitations, multiplicity of analyses, results from similar studies, and other relevant evidence	10-11
Generalisability	21	Discuss the generalisability (external validity) of the study results	10-11
**Other information**			
Funding	22	Give the source of funding and the role of the funders for the present study and, if applicable, for the original study on which the present article is based	13

*Give information separately for cases and controls in case-control studies and, if applicable, for exposed and unexposed groups in cohort and cross-sectional studies.

Note: An Explanation and Elaboration article discusses each checklist item and gives methodological background and published examples of transparent reporting. The STROBE checklist is best used in conjunction with this article (freely available on the Web sites of PLoS Medicine at http://www.plosmedicine.org/, Annals of Internal Medicine at

http://www.annals.org/, and Epidemiology at http://www.epidem.com/). Information on the STROBE Initiative is available at www.strobe-statement.org.

© **2026 Hu S. et al.**
